# Blood lipid-related low-frequency variants in *LDLR* and *PCSK9* are associated with onset age and risk of myocardial infarction in Japanese

**DOI:** 10.1038/s41598-018-26453-x

**Published:** 2018-05-25

**Authors:** Tomoyuki Tajima, Hiroyuki Morita, Kaoru Ito, Tsutomu Yamazaki, Michiaki Kubo, Issei Komuro, Yukihide Momozawa

**Affiliations:** 1 Department of Cardiovascular Medicine, Graduate School of Medicine, The University of Tokyo, Tokyo, Japan; 2 Laboratory for Genotyping Development, RIKEN Center for Integrative Medical Sciences, Kanagawa, Japan; 3 Laboratory for Cardiovascular Disease, RIKEN Center for Integrative Medical Sciences, Kanagawa, Japan; 4 Center for Epidemiology and Preventive Medicine, The University of Tokyo Hospital, Tokyo, Japan

## Abstract

Recent studies have revealed the importance of rare variants in myocardial infarction (MI) susceptibility in European populations. Because genetic architectures vary in different populations, we investigated how they contribute to MI susceptibility in Japanese subjects. We performed targeted sequencing of 36 coronary artery disease risk genes, identified by genome-wide association studies, in 9,956 cases and 8,373 controls. Gene-based association tests identified significant enrichment of rare variants in *LDLR* and *PCSK9* in MI cases. We identified 52 (novel 22) *LDLR* variants predicted to be damaging. Carriers of these variants showed a higher risk of MI (carriers/non-carriers 89/9867 in cases, 17/8356 controls, OR = 4.4, *P* = 7.2 × 10^−10^), higher LDL-cholesterol levels and younger age of onset for MI. With respect to *PCSK9*, E32K carriers showed higher LDL-cholesterol levels and younger age of onset for MI, whereas R93C carriers had lower LDL-cholesterol levels. A significant correlation between LDL-cholesterol levels and onset age of MI was observed in these variant carriers. In good agreement with previous studies in patients with familial hypercholesterolaemia, our study in the Japanese general population showed that rare variants in *LDLR* and *PCSK9* were associated with the onset age of MI by altering LDL-cholesterol levels.

## Introduction

Despite advances in therapeutic strategies, myocardial infarction (MI) remains a leading cause of morbidity and mortality worldwide^1^. To clarify the complex heritability of MI^2^, large-scale genome-wide association studies (GWAS) were performed to identify more than 160 susceptibility loci for coronary artery disease (CAD)^3–8^. Moreover, resequencing analyses^9–13^ have revealed that rare variants in lipid-related genes contribute to the susceptibility for MI.

Although genes associated with diseases are shared among populations, disease-associated rare variants are subject to variation depending on the population. For example, a genomic analysis using an exome array demonstrated that the association of low-frequency variants with blood lipids or CAD was different between participants of European ancestry and African ancestry^13^. The protective effect of *ANGPTL4* low-frequency variant on CAD was reported in a European population^10,11^ but was not observed in a Chinese population^12^. Thus, these uneven distributions of rare variants could be explained by population differences. Therefore, their effects should be evaluated using a large number of samples in each population to successfully develop population-specific precision medicine, in which the most appropriate preventive therapy could be chosen based on a population-specific genetic risk profile.

Additionally, judging from the recent findings^10–13^ that significant rare variants associated with CAD reside in GWAS-identified genes for CAD (e.g., *LDLR*, *PCSK9*, *APOB*), the GWAS-identified genes could be good targets for rare variant discovery. The significance of GWAS-identified genes in rare and functional variant discovery has also been demonstrated in other studies on dyslipidaemia^14,15^. Based on these genetic findings, to detect efficiently the rare variants associated with CAD, we adopted a strategy for performing targeted sequencing of 36 genes from CAD-associated GWAS loci reported up to the beginning of our present study and conducted an association analysis using 9,956 cases and 8,373 controls in the Japanese population. The aim was to better understand the contribution of rare variants to the susceptibility of MI, followed by proposing a possible preventive strategy for Japanese.

## Results

### Summary Of Two-Stage Targeted Sequencing

**Figure 1:**
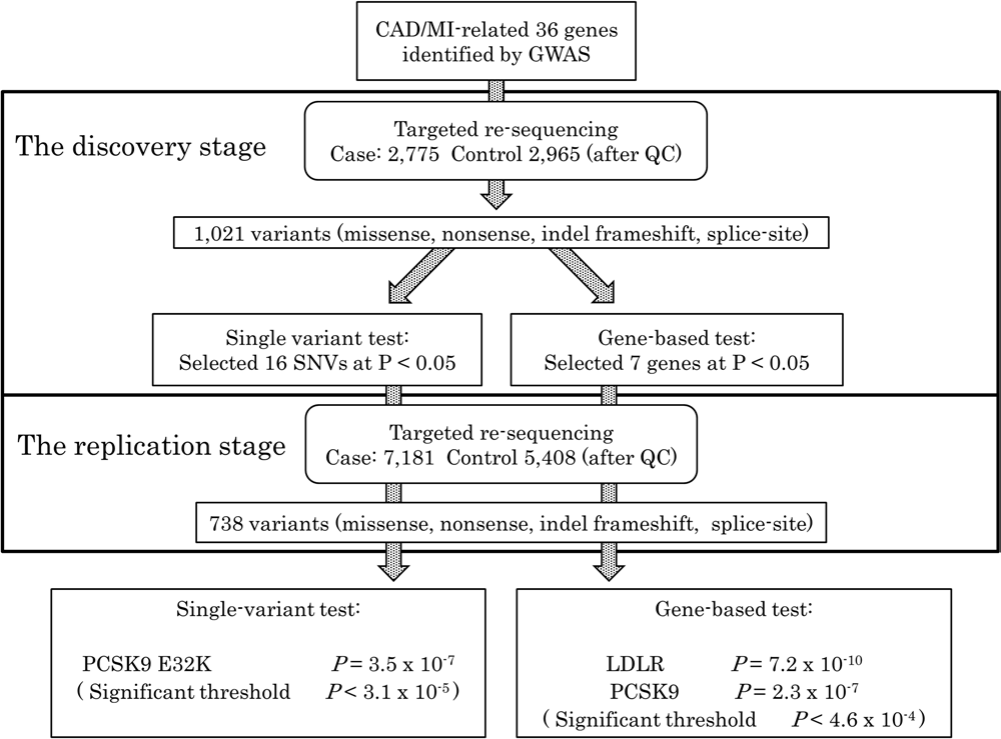
Overall design for the two-stage targeted sequencing study. Missense, nonsense, indel frameshift and splice-site variants with minor allele frequency less than 5% were tested after excluding the synonymous variants. In the single variant test, we set the study-wide significance threshold to *P* = 3.1 × 10^−5^. In the gene-based test, we set the study-wide significance threshold to *P* = 4.6 × 10^−4^.

In the discovery stage, targeted sequencing of 36 genes (90,823 bp) was performed in 2,811 cases and 2,974 controls (Table 1) and covered 98.9% of targeted bases with a minimum of 20-fold depth (DP) (Supplementary Fig. S1). After QC, 1,630 variants (minor allele frequency (MAF) < 0.05) were detected in 2,775 cases and 2,965 controls (Fig. 1) (Supplementary Tables S2 and S3). Of these variants, 1,235 were novel, among which 508 and 465 novel ones were observed only in cases and controls, respectively, and 262 novel ones were identified in both groups. After excluding the synonymous variants, we performed single-variant and gene-based association analyses using 1,021 single nucleotide variant (SNV) of missense and nonsense, indel frameshift and splice-site variants with a minor allele frequency (MAF) < 0.05. Single-variant association analysis identified 16 SNVs that showed *P* < 0.05 in Fisher’s exact test (Supplementary Table S4). Gene-based association was analysed with the Cohort Allelic Sum Test (CAST)^16^ and Sequence Kernel Association Test (SKAT)^17^, and we found 7 genes (*PCSK9*, *GUCY1B3*, *PLG*, *ICA1L*, *NBEAL1*, *TCTN1* and *LDLR*) that showed *P* < 0.05 in at least one of the following three variant categories: (1) all non-synonymous variants; (2) damaging, defined by all disruptive (null) variants and missense variants annotated as deleterious by all five protein function prediction algorithms, PolyPhen-2 HumDiv, Polyphen2-HumVar^18^, SIFT^19^, MutationTaster^20^ and LRT^21^ score; and (3) disruptive (null) variants (nonsense, indel frameshift and splice-site variants) (Supplementary Table S5). In the replication stage, the 11 genes including the 16 SNVs and 7 genes that showed an association in the discovery stage were sequenced (39,944 bp) in 7,316 independent cases and 5,828 independent controls (Table 1), 98.2% of the targeted bases were covered with DP ≥ 20 (Supplementary Fig. S2), and 1,028 variants were identified in 7,181 cases and 5,408 controls after quality control (Fig. 1) (Supplementary Table S6). Of these variants, 791 were novel, among which 356 and 236 novel ones were observed only in cases and controls, respectively, and 199 novel ones were identified in both groups. Among 7 genes targeted in both the discovery and replication stages, 587 novel variants were identified in the discovery stage, and 118 were repeatedly identified in the replication stage. That is, 469 other novel variants were identified only in the discovery stage.

**Table 1:** Baseline characteristics of study participants in the discovery and replication stages.

Stage	Phenotype	N	Mean Age (SD)	Mean onset-age of MI (SD)	Source	Male (%)	Smoking (%)	MeanBMI (SD)	Mean systolic BP (mmHg) (SD)	Mean LDL-C (mg/dL) (SD)	Mean HDL-C (mg/dL) (SD)	Mean TG (mg/dL) (SD)	Mean HbA1c(%)(SD)
Discovery	Cases	2,775	62.7(10.9)	57.6(10.8)	BBJ	84.5	74.2	24.2 (3.4)	126.8 (16.4)	111.8 (31.8)	47.5 (12.6)	156.2 (110.5)	6.63 (1.59)
	Controls	2,965	56.4(12.1)	—	PSC(N = 963)	61.0	NA	NA	NA	NA	NA	NA	NA
					MRC(N = 1,060)	74.4	54.7	23.6 (3.2)	130.9 (17.9)	NA	NA	NA	NA
					Univ. of Tokyo(N = 942)	64.6	41.2	22.4 (3.9)	114.8 (23.4)	125.7 (46.6)	65.6 (17.4)	113.8 (70.4)	5.72 (0.85)
Replication	Cases	7,181	68.0(9.8)	62.2(10.4)	BBJ	80.9	72.0	23.8 (3.3)	129.3 (16.9)	111.6 (31.6)	48.9 (14.6)	147.5 (93.7)	6.40 (1.45)
	Controls	5,408	62.8(9.8)	—	BBJ	53.4	59.6	22.4 (3.8)	128.8 (17.0)	119.2 (34.7)	57.5 (17.0)	132.0 (94.8)	6.16 (1.37)

In controls in the discovery stage, blood test data were available only from University of Tokyo samples. Abbreviations: BBJ, Biobank Japan project; PSC, Pharma SNP consortium; Univ. of Tokyo, The University of Tokyo Hospital; SD, standard deviation; T-Chol, total cholesterol; LDL-C, low-density lipoprotein cholesterol; HDL-C, high-density lipoprotein cholesterol; TG, triglycerides; and NA, blood test data not available.

### Results of association test

In the meta-analysis of single-variant association tests (Table 2), we found one missense variant that showed a study-wide significant association with MI: chr1:g.55505604 G > A (*PCSK9*: E32K, OR = 1.7, *P* = 3.5 × 10^−7^).

**Table 2:** Significant association results of single-variant and gene-based tests.

**Single-variant test**												
Chr	Position (hg19)	Alt	Ref	Gene	Type/Category	AA change	Stage	AA/AG/GG (MAF) in cases	AA/AG/GG (MAF) in controls	OR	(95% CI)	P*
1	55505604	A	G	*PCSK9*	Missense/Non-synonymous	E32K	Discovery	1/86/2685 (0.016)	0/47/2916 (0.0079)	2.0	(1.4, 2.9)	8.6 × 10^−5^
							Replication	4/201/6976 (0.015)	3/97/5307 (0.0095)	1.5	(1.2, 1.9)	3.5 × 10^−4^
							Combined	5/287/9661 (0.015)	3/144/8223 (0.0090)	1.7	(1.4, 2.0)	3.5 × 10^−7^
**Gene-based test**												
**Gene**				**Grouping (test method)**			**Stage**	**Carriers/non-carriers (%Freq) in cases**	**Carriers/non-carriers (%Freq) in controls**	**OR**	**(95% CI)**	***P***
*LDLR*				Damaging (CAST)			Discovery	28/2747 (1.0)	5/2960 (0.17)	6.0	(2.3, 20)	2.6 × 10^−5^
							Replication	61/7120 (0.85)	12/5396 (0.22)	3.9	(2.1, 7.9)	2.1 × 10^−6^
							Combined	89/9867 (0.89)	17/8356 (0.20)	4.4	(2.6, 7.4)	7.2 × 10^−10^
*PCSK9*				All non-synonymous (SKAT)			Discovery	—	—	—	—	3.7 × 10^−5^
							Replication	—	—	—	—	7.5 × 10^−4^
							Combined	—	—	—	—	2.3 × 10^−7^
				Gain-of-function (CAST)			Discovery	159/2616 (5.7)	110/2855 (3.7)	1.5	(1.2, 2.0)	3.5 × 10^−4^
							Replication	394/6787 (5.4)	262/5146 (4.8)	1.2	(1.0, 1.4)	2.4 × 10^−2^
							Combined	553/9403 (5.6)	372/8001 (4.5)	1.3	(1.1, 1.5)	1.0 × 10^−4^
				Loss-of-function (CAST)			Discovery	33/2742 (1.2)	72/2893 (2.4)	0.49	(0.32, 0.76)	7.8 × 10^−4^
							Replication	123/6933 (1.7)	125/5283 (2.3)	0.74	(0.58, 0.97)	2.7 × 10^−2^
							Combined	156/9675 (1.6)	197/8176 (2.4)	0.66	(0.53, 0.81)	1.1 × 10^−4^

Single-variant test:

A result exceeding the pre-defined study-wide significance (*P* < 3.1 × 10^−5^). *P* values were calculated using Fisher’s exact test. Combined *P* values were calculated using the Cochran-Mantel-Haenszel method. Abbreviations: AA, amino acid; MAF, minor allele frequency; OR, odds ratio; 95% CI, 95% confidence interval; Ref, the reference allele based on hg19; Alt, the alternative allele.

Gene-based test:

Significant results exceeding the gene-wide significance (*P* < 4.6 × 10^−4^). Abbreviations: Freq, percentage of cases or controls carrying at least one categorized variant; OR, odds ratio.

In a meta-analysis of gene-based association tests (Table 2), we identified significant associations in *LDLR* and *PCSK9*. In *LDLR*, we found a total of 138 non-synonymous variants, in which 52 damaging variants with 14 disruptive ones were identified. In damaging variants, 22 out of 52 were novel variants, while 6 out of 14 were novel in disruptive variants. All *LDLR* disruptive variants were confirmed by Sanger sequencing. The results of gene-based tests in the three variant categories are shown in Supplementary Table S7. The damaging variants showed significant enrichment (OR = 4.4, *P* = 7.2 × 10^−10^) in MI cases. With respect to the disruptive variants, we found a stronger genetic effect (OR = 15) on MI risk, but the association was weakened (*P* = 5.8 × 10^−7^), potentially due to the small number of samples (36 in cases and 2 in controls).

In *PCSK9*, we found 94 non-synonymous, 18 damaging and 6 disruptive variants. We found a significant association with MI in all non-synonymous *PCSK9* variants by SKAT (*P* = 2.3 × 10^−7^), but that association was not detected by CAST. The damaging and disruptive variants did not show significant associations. Because we observed that E32K was a risk variant for MI whereas R93C was protective in the single-variant test (Supplementary Table S4), *PCSK9* appears to harbour rare variants with opposite effects: deleterious and protective. To clarify the possibility that the co-existence of these opposite-effect variants might offset the association of *PCSK9* variants with MI, we classified variants into a gain-of-function (GoF) group and a loss-of-function (LoF) group based on the Leiden Open (source) Variation Database(LOVD)^22–24^. As a result, we found significant associations in both the GoF group (OR = 1.3, *P* = 1.0 × 10^−4^) and the LoF group (OR = 0.7, *P* = 1.1 × 10^−4^). However, these associations were not significant after excluding E32K or R93C (*P* = 0.95 after excluding E32K in the GoF group and *P* = 0.70 after excluding R93C in the LoF group). These results suggest that E32K and R93C in *PCSK9* have a predominant effect in the gene-based test for *PCSK9*. All tested categories of *PCSK9* variants are shown in Supplementary Table S7.

In 5 MI cases, a damaging *LDLR* variant and *PCSK9* E32K variant were detected, while this coexistence did not occur in any control subject.

### Clinical phenotypes of rare variant carriers of *LDLR* and *PCSK9*

To assess the clinical impact of rare variants in *LDLR* and *PCSK9*, we examined the effects of these variants on serum LDL cholesterol levels and the onset age of MI. We also examined HDL cholesterol levels and triglycerides levels; however, we did not observe any statistically significant relationship with those variants.

When we compared LDL cholesterol levels among the three variant categories of *LDLR* in all subjects (cases and controls) whose prescription data and lipid profile data were both available (Table 3), LDL cholesterol levels in disruptive variant carriers were significantly higher than those in non-carriers, who did not have any *LDLR* or *PCSK9* rare variants (+48.5 mg/dl, *P* = 7.6 × 10^−13^). LDL cholesterol levels were also higher in non-synonymous variant carriers and damaging variant carriers than in non-carriers (+5.0 mg/dl, *P* = 0.012, + 44.4 mg/dl, *P = *6.9 × 10^−17^, respectively). Next, we examined the effect of *LDLR* rare variants on the age of MI onset in cases (Table 4). The onset age in disruptive variant carriers was significantly younger than those in non-carrier MI patients (−11.2 years, *P* = 5.2 × 10^−10^). The onset age in damaging carriers was also younger than that in non-carriers (−4.9 years, *P* = 1.3 × 10^−3^). With respect to *PCSK9*, we compared LDL cholesterol levels and the onset age of MI among the following groups: E32K carriers, R93C carriers and disruptive variant carriers. When we compared LDL cholesterol levels among these groups (Table 3), the E32K carriers showed higher LDL cholesterol levels than the non-carriers, who did not have any *LDLR* or *PCSK9* variants (+18.0 mg/dl, *P* = 1.6 × 10^−14^), whereas R93C carriers had lower LDL cholesterol levels than non-carriers (−12.4 mg/dl, *P* = 5.6 × 10^−5^). In the disruptive variant group, LDL cholesterol levels were significantly lower than those in non-carriers (−38.5 mg/dl, *P* = 0.012). In the analysis of the onset age of MI in cases (Table 4), R93C carriers did not show a significant alteration (+0.22 years, *P* = 0.81). However, E32K carriers showed earlier onset than did non-carrier MI patients (−2.3 years, *P* = 3.7 × 10^−4^), whereas disruptive variant carriers showed later onset of MI (+8.2 years, *P* = 0.043). These results suggested that rare variants in *LDLR* and *PCSK9* had a predominant effect on the onset age of MI in the MI patients. Three subjects who carried both E32K and R93C in *PCSK9* were excluded from the analyses.

**Table 3:** Effects of *LDLR* and *PCSK9* rare variants on LDL cholesterol levels.

Gene	Category	N	LDL-C (mg/dl)	Change from Non-Carriers of Rare *LDLR*/*PCSK9* Variants			
			mean ± SD	Crude (95% CI)	*P*	Adjusted^†^ (95% CI)	*P*
Non-Carriers of Rare *LDLR*/*PCSK9* Variants		5080	112.45 ± 31.61				
*LDLR*	non-synonymous	261	117.16 ± 37.98	+4.71 (+0.73, +8.68)	0.02	+5.00 (+1.12, +8.87)	0.012
*LDLR*	Damaging	34	155.08 ± 37.19	+42.63 (+31.95, +53.31)	5.97 × 10^−15^	+44.42 (+34.03, +54.81)	6.85 × 10^−17^
*LDLR*	Disruptive	21	155.77 ± 33.97	+43.32 (+29.76, +56.87)	4.02 × 10^−10^	+48.49 (+35.26, +61.72)	7.64 × 10^−13^
*PCSK9*	E32K	186	128.74 ± 42.57	+16.29 (+11.59, +20.98)	1.12 × 10^−11^	+17.97 (+13.39, +22.55)	1.64 × 10^−14^
*PCSK9*	R93C	102	102.18 ± 34.91	−10.27 (−16.48, −4.06)	1.2 × 10^−3^	−12.44 (−18.49, −6.39)	5.62 × 10^−5^
*PCSK9*	Disruptive	4	80.70 ± 27.17	−31.75 (−62.75, −0.76)	0.045	−38.49 (−68.64, −8.34)	0.012

Changes from non-carriers and confidence intervals were calculated from linear regression models.

^†^Adjusted for age, gender, BMI, smoking status and cholesterol lowering medications.

Abbreviations: LDL-C, low-density lipoprotein cholesterol; SD, standard deviation; 95% CI, 95% confidence interval.

**Table 4:** Effects of *LDLR* and *PCSK9* rare variants on onset ages of MI.

Gene	Category	N	Onset Age of MI (year old)	Change from Non-Carriers of Rare *LDLR*/*PCSK9* Variants			
			mean ± SD	Crude (95% CI)	*P*	Adjusted^†^ (95% CI)	*P*
Non-Carriers of Rare *LDLR*/*PCSK9* Variants		6091	61.10 ± 10.63				
*LDLR*	non-synonymous	317	60.46 ± 10.58	−0.64 (−1.84, +0.56)	0.29	−0.57 (−1.69, +0.56)	0.32
*LDLR*	Damaging	43	56.12 ± 11.43	−4.98 (−8.18, −1.79)	2.2 × 10^−3^	−4.91 (−7.89, −1.92)	1.30 × 10^−3^
*LDLR*	Disruptive	31	48.58 ± 10.94	−12.52 (−16.27, −8.77)	6.67 × 10^−11^	−11.15 (−14.66, −7,64)	5.17 × 10^−10^
*PCSK9*	E32K	253	58.72 ± 10.82	−2.38 (−3.72, −1.04)	5.0 × 10^−4^	−2.27 (−3.53, −1.02)	3.70 × 10^−4^
*PCSK9*	R93C	112	61.45 ± 10.36	+0.35 (−1.64, +2.33)	0.73	+0.22 (−1.64, +2.08)	0.81
*PCSK9*	Disruptive	6	73.83 ± 10.68	+12.73 (+4.22, +21.24)	3.4 × 10^−3^	+8.21 (+0.25, +16.17)	0.043

Changes from non-carriers and confidence intervals were calculated from linear regression models.

^†^Adjusted for gender, BMI and smoking status and cholesterol lowering medications.

Abbreviations: SD, standard deviation; 95% CI, 95% confidence interval.

Both *LDLR* and *PCSK9* are known to be causative genes for familial hypercholesterolemia (FH), which is a well-known risk for MI. The FH database^23,24^ contains some of the variants detected in this study in *LDLR* and *PCSK9* (59/138 = 43% and 23/94 = 24%, respectively) (Supplementary Tables S8 and S9). To examine the effect of newly identified variants in this study, we removed these previously known variants from our data and explored how these associations changed among the three categories that showed a significant association with MI. As a result, each category showed drastic attenuation of signals (*LDLR* damaging *P* = 0.027, *LDLR* disruptive *P* = 0.13 and *PCSK9* all non-synonymous (SKAT) *P* = 0.58), where statistical power was hampered, potentially due to the decreased number of variant carriers. After subtraction of these variant carriers, we had a limited number of samples (disruptive variant carriers in *LDLR* decreased from 38 to 6, damaging variant carriers in *LDLR* decreased from 106 to 24, and *PCSK9* all non-synonymous variant carriers decreased from 4,748 to 277) (Supplementary Table S10). When we examined the LDL cholesterol levels and the onset age of MI using newly identified variants in *LDLR* and *PCSK9* alone (Supplementary Tables S11 and S16), the effects of newly identified variants exhibited the same trend as those of previously known FH variants, where the statistical power was weakened, potentially due to the decreased number of variant carriers.

Dividing the study population according to lipid-lowering therapy or gender, subgroup analyses on the genetic effects of variants on serum LDL cholesterol levels or the onset age of MI were performed. As described above, the LDL cholesterol level analyses were conducted for all (case and control) subjects whose prescription data and lipid profile data were available, and the onset-age analyses were performed for all cases. In our study, the percentage of subjects treated by cholesterol-lowering medications was much greater in MI cases (47%) than in controls (9.1%), consistent with the standard LDL cholesterol-lowering therapy. Although the statistical power was weakened potentially due to the decreased number of carriers in each variant category, nearly the same trend was observed for the genetic effects of variants on serum LDL cholesterol levels in all subgroups (Supplementary Tables S12–15). However, for the genetic effects of variants on the onset age of MI, nearly the same trend was observed in subjects with cholesterol-lowering drugs (Supplementary Table S18) and male patients (Supplementary Table S19), whereas the trend was not observed in subjects without cholesterol-lowering drugs (Supplementary Table S17) and female patients (Supplementary Table S20).

**Figure 2:**
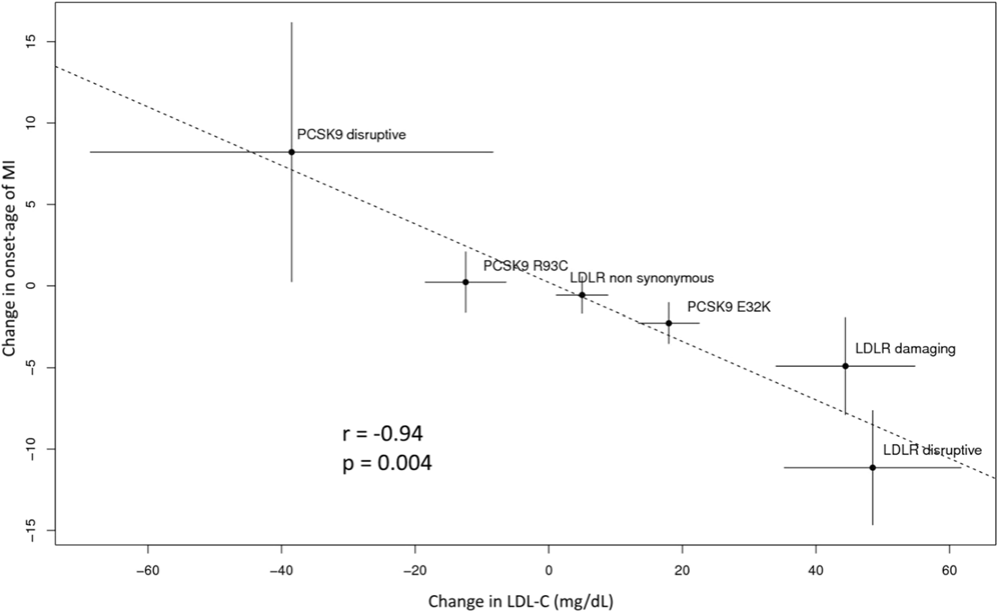
Effects of *LDLR* and *PCSK9* rare variants on LDL-C levels and onset age of MI. Dots represent the change from non-carriers of *LDLR*/*PCSK9* rare variants for each group and lines indicate the 95% confidence interval. Effects were estimated using multiple linear regression models adjusted for age, gender, BMI, smoking status and cholesterol lowering medications in assessing LDL cholesterol levels, and controlling for the same parameters except age in assessing onset age of MI. Abbreviations: r: Pearson’s correlation coefficient.

To clarify the relationship between LDL cholesterol levels and MI onset in the association of *LDLR* and *PCSK9* rare variants, we compared the changes in LDL cholesterol levels and changes in the onset age of MI estimated in the evaluation of the clinical phenotypes (Fig. 2). We found a significant linear correlation between these two factors in all variant categories (r = −0.94, *P* = 0.004). Moreover, a similar trend was observed in patients without previously known FH variants (r = −0.95, *P* = 0.054) (Supplementary Fig. S3). Dividing the study population according to lipid-lowering therapy or gender, subgroup analyses of this relationship were performed. A significant linear correlation was demonstrated in patients without cholesterol-lowering drugs (r = −0.96, *P* = 0.003), and a similar trend was observed in patients with cholesterol-lowering drugs (r = −0.85, *P* = 0.068) (Supplementary Fig. S4). In male patients, a significant linear correlation in all variant categories was observed (r = −0.91, *P* = 0.013), and a similar trend was observed in female patients (r = −0.79, *P* = 0.062) (Supplementary Fig. S5). Collectively, rare variants in these genes exert their effect on the onset age of MI in large part by altering LDL cholesterol levels.

## Discussion

This study is the first to address the relationship between rare variants and MI in a large Japanese population. We identified a significant association of rare variants in *LDLR* and *PCSK9* with MI. Our analysis revealed that rare variants in *LDLR* and *PCKS9* affect onset age of MI by altering serum LDL cholesterol levels.

These dyslipidaemia-related genes are found to be susceptibility genes for MI in Japanese, which is consistent with observations made for different ethnic populations^25–27^. However, the contents of rare variants in these genes are different. When we examined the individual variants identified in the present study, 55 *LDLR* and 43 *PCSK9* variants were not in the ExAC or FH databases, implying that they are novel and unique in the Japanese population (Supplementary Tables S8 and S9). In the Japanese population, judging from CAST and SKAT results, we observed a unidirectional effect of *LDLR* variants composed of loss-of-function variants with deleterious consequences for MI and a bidirectional effect of *PCSK9* variants: *PCSK9* E32K, which is well known as a FH-causing variant, is a gain-of-function variant with a deleterious effect for MI, while loss-of-function *PCSK9* disruptive variants have a protective effect on the onset of MI. Indeed, the bidirectional effect of *PCSK9* variants on both LDL cholesterol levels and MI risk were previously reported in studies of Mendelian dyslipidaemia^28,29^. Additionally, this bidirectional effect of *PCSK9* variants on LDL cholesterol levels was observed in the general population^30^, but the relationship between the bidirectional effect and MI was not discussed. Hence, we are the first to present the bidirectional effect of *PCSK9* rare variants on MI risk via LDL cholesterol levels in a population-based study.

We demonstrated a linear correlation between the changes in LDL cholesterol levels and changes in onset age of MI in carriers of *LDLR* or *PCSK9* rare variants, which implies that rare variants in *LDLR* and *PCSK9* influence the onset age of MI potentially by altering serum LDL cholesterol levels. This finding supports the idea that normalizing LDL cholesterol levels in carriers of *LDLR* or *PCSK9* rare variants should be effective in preventing MI onset. A long-term cohort study provided supportive evidence indicating that statin therapy for normalizing LDL cholesterol levels in FH patients lowered the risk of MI onset to the same level as that in non-FH patients^31^. Therefore, given that carriers of *LDLR* or *PCSK9* rare variants continue to be exposed to genetic effects after birth and accumulate the risk of MI, we propose that we should check *LDLR* and *PCSK9* rare variants in patients with juvenile-onset hyper-LDL cholesterolaemia, whether a diagnosis of FH is made, and that a preemptive therapy for normalizing LDL cholesterol levels should be undertaken to prevent MI as long as patients have *LDLR* rare variants or *PCSK9* gain-of-function variants. Notably, judging from our findings that the genetic effects of rare variants on LDL cholesterol level and onset age of MI were observed even in patients with cholesterol-lowering therapy, the ongoing lipid-lowering therapy in clinical practice might be insufficient to cancel the rare variants-associated hyper-LDL cholesterolaemia and MI.

Even with our careful curation of candidate genes for targeted sequencing, potential limitations remain. First, a study in a European population showed that rare alleles in *APOA5* contributed to the risk for early onset MI^25^. However, we did not employ *APOA5* because it did not meet our prespecified criteria. Second, although our gene selection was mainly based on the loci provided by the CARDIoGRAMplusC4D Consortium^7^. We performed the gene selection in 2013, after which the latest studies^8,32^ expanded the spectrum of GWAS loci for CAD and increased candidate genes for MI susceptibility. An additional analysis of rare variants in newly identified genes might be needed. Third, the classification into damaging variants and disruptive (null) ones was decided by prediction algorithms and not verified by experimental data. A similar classification was used in a previous study^25^ in which “deleterious (strict)” variants corresponded to our “damaging” variants. Fourth, we did not check and exclude subjects with FH, although our list for targeted sequencing included previously known FH genes. In our study, 43% of *LDLR* and 24% of *PCSK9* rare variants were previously reported FH mutations. However, it is natural to find subjects with known FH mutations in the general population because other population-based studies have also identified variants previously described as causing FH^25,27^.

Despite these limitations, our analyses shed light on the Japanese-specific genetic architecture of MI risk driven by rare and low-frequency variants and elucidate a correlative link between LDL cholesterol level and onset age of MI in the presence of *LDLR* or *PCSK9* rare variants. Recent parent-child genetic screening^33^ for previously documented 48 FH mutations (including 46 *LDLR* mutations, 1 *APOB* one, and 1 *PCSK9* one) revealed a relatively low prevalence (0.8%) of such carriers. However, our targeted sequencing demonstrated that carriers of rare variants in dyslipidaemia-related genes are more prevalent in the general population. Compared with panel screening of previously reported FH mutations, wide screening using targeted sequencing might be more valuable because it could identify rare variants associated with LDL cholesterol levels and MI risk even if each variant has milder effects than those of known FH mutations. Given that dyslipidaemia-related genes could contribute to the pathogenesis of MI even under lipid-lowering therapy, more potent treatment of dyslipidaemia than ever should be recommended as a promising tool for prevention of MI. Identification of rare and low-frequency variants will provide a clue for clarifying the complex genetic architecture of MI risk as well as a rationale for the appropriate treatment against MI, which emphasizes the usefulness of targeted sequencing of candidate genes.

## Methods

### Study design

All methods were performed in accordance with the relevant guidelines and regulations.

A targeted sequencing was performed in two stages as shown in Fig. 1. In the discovery stage, we performed targeted sequencing in coding regions of 36 genes using 2,775 MI cases and 2,965 controls. We performed single-variant and gene-based association analysis and selected variants or genes that showed a *P* value of less than 0.05 in the discovery stage. These variants or genes were examined using 7,181 independent cases and 5,408 independent controls in the replication stage. After the two stages of sequencing, a meta-analysis was performed. We only used variants annotated as missense, nonsense, indel frameshift or splice-site with a minor allele frequency (MAF) of less than 5% throughout this study.

### Study samples

All MI cases including discovery and replication stages were obtained from the BioBank Japan project^34,35^, which constructed a patient-oriented biobank that collected DNA samples from 200,000 patients suffering from at least one of 47 target diseases including MI between 2003 and 2008. As previously described^36^, cases in both stages were selected based on medical records and confirmed to satisfy both of the following criteria: (1) left ventricular wall motion abnormalities on echocardiography and (2) one or more coronary artery occlusion on angiography. If patients had experienced multiple MI events, the first episode was considered “onset of MI” in the onset-age analysis.

Controls in the discovery stage were collected from three different sites: Pharma SNP Consortium (PSC), Osaka-Midousuji Rotary Club (MRC) and the University of Tokyo Hospital. MRC and PSC samples were self-reported healthy volunteers. Controls from the University of Tokyo Hospital were examinees who underwent a health check-up, and individuals with a history of CAD were excluded. Controls for the replication stage were a mixture of cases registered in the Biobank Japan that had been used as GWAS controls in previous reports^36,37^. These control subjects consisted of patients with 5 diseases (cerebral aneurysm, oesophageal cancer, endometrial cancer, chronic obstructive pulmonary disease and glaucoma). Individuals with CAD were excluded from controls. All individuals were of Japanese ancestry and provided written informed consent to participate in this study. This study was approved by the ethics committees of the University of Tokyo and RIKEN Center for Integrative Medical Sciences.

### Gene selection

We selected target genes based on the GWAS of the CARDIoGRAMplusC4D Consortium^7^, which identified 47 CAD loci including 63 genes. We added 3 genes located at two East Asian specific loci from Han-Chinese GWAS^6^. In addition, we selected 9 genes in linkage disequilibrium (r^2^ > 0.5) with top SNPs in these GWASs according to the 1000 Genomes Projects^38^ Phase 3 data in studied populations. Furthermore, we added 8 genes that might have a relationship with the susceptibility to atherosclerosis based on an expression quantitative trait locus analysis of mouse and human vascular cells^39^. Consequently, we selected 83 genes located at 49 loci.

To search for variants with clinical implications, we selected genes satisfying one of the following criteria: (1) there was an established assay for measurement of encoded proteins or genes had been known to be “druggable”^40^ and (2) the gene-deficient mouse models recapitulated CAD/MI-related phenotypes. Finally, we selected 36 genes located at 19 CAD susceptibility loci (Supplementary Table S1).

### Library preparation

We performed multiplex PCR-based targeted sequencing^41^. Coding DNA sequences (CDS) for 36 targeted genes were defined according to the Consensus CDS (CCDS) database release 15^42^. Long CDS were divided into 180 base pairs (bps) fragments. We designed, tested and optimized PCR primers for a total of 690 short fragments. Targeted regions were then amplified by the parallel multiplex PCR using a Platinum Multiplex PCR Master Mix (Life Technologies, Carlsbad, CA, USA). After PCR reaction, adaptors with embedded unique 8 base index sequences were ligated to PCR products using KAPA Library Amplification Kit (KAPA Biosystems, Wilmington, MA, USA). Libraries were purified with magnetic beads (AMPure XP), and quantified using a 2100 Bioanalyzer (Agilent, Santa Clara, CA, USA) and qPCR assay (KAPA Biosystems). We used the HiSeq 2500 v2 cluster chemistry (Illumina, San Diego, CA, USA) as a sequencing platform.

### Read mapping and variant analysis

Sequencing data were processed with bcl2fastq (version 1.8.4) and converted to fastq files. PCR primer sequences were removed with cutadapt^43^ (version 1.8). Next, the sequences were aligned to a human genome reference (hg19) using a Burrows-Wheeler Aligner^44^ (BWA, version 0.7.5). Aligned read files (Sequence Alignment/Map format: sam) were binarized (bam), indexed with SAMTools^45^ (version 0.1.19), and processed using a Genome Analysis ToolKit^46^ (GATK version 3.2.2). Reads were locally realigned by GATK IndelRealigner, and variant detection was performed by both a UnifiedGenotyper and HaplotypeCaller, separately. In the HaplotypeCaller process, multiple bam files from the same sample were called simultaneously using “-ERC GVCF” mode, and then all files were jointly genotyped by GATK GenotypeGVCFs. In the UnifiedGenotyper process, a hard filter was applied by GATK VariantFiltration using the following filter parameters: FisherStrand >40.0, QualByDepth <2.0, RMSMappingQuality <40.0 and MappingQualityRankSumTest <−4.0. Finally, two outputs from different callers were merged into one variant call format (VCF) file, and the original bam files were then genotyped again using this merged VCF file by UnifiedGenotyper in GENOTYPE_GIVEN_ALLELES mode.

### Quality Control

In addition to the hard filtration mentioned above, variants were excluded if the DP for any base was less than 20 or had a missing rate of >0.05 or a Hardy-Weinberg equilibrium (HWE) *P* value of <1 × 10^−5^ in controls. To filter out further false-positive variants, we created histograms of non-reference allele frequency for all called variants and checked by visual inspection whether each histogram consisted of 3 distinct clusters with its peaks at 0, 0.5 and 1.0. Variants that showed abnormal histogram patterns (e.g., continuous distribution) were excluded. Sample-level quality control measures were also performed. Samples with a ratio of mapped reads to total reads less than 0.6 or samples that could not achieve a minimum of 20-fold coverage for at least 95% of the targeted bases were excluded.

To estimate the accuracy of our method, we sequenced 76 Hapmap Japanese-Han Chinese samples. Comparing the variant calls with the 1000 Genomes Project^38^ Phase 3 data (1000 g), we found 188 variants, while 1000 g samples had 185 in the same targeted region. Among these, 184 were called in both, 1 was found only in 1000 g, and 4 were found only in our sample. This finding implies that our analysis has a sensitivity of 99.5% (184/185) and a positive predictive value of 97.9% (184/188). Furthermore, we assessed the concordance of genotyped data using 2,360 overlapping samples genotyped by Illumina Human610-Quad Beadchips in our previous GWAS^36^. The concordance between our analysis and the chip data using 187 shared variants was 99.94% (440,765/441,051).

### Variant annotation

Variants were annotated with SnpEff^47^ using the GRCh37.75 database. For variants with different annotations due to multiple transcripts of the gene, the highest impact effect for each variant was selected.

### Statistical analyses

We performed single-variant association analysis by 2 × 2 Fisher’s exact test using Plink software^48^ (Version 1.07). A meta-analysis for the two stages was performed with the Cochran-Mantel-Haenszel (CMH) method. We set the study-wide significance threshold for single-variant association test to 3.1 × 10^−5^, based on a Bonferroni correction for 1,630 QC passed variants in the discovery stage.

For gene-based association analysis, we employed SKAT^17^ and CAST^16^. We categorized variants into three sets of variants based on multiple protein function prediction algorithms: (1) all non-synonymous variants; (2) damaging, defined by all disruptive variants and missense variants annotated as deleterious by all five protein function prediction algorithms, PolyPhen-2 HumDiv, Polyphen2-HumVar, SIFT, MutationTaster and LRT score; and (3) disruptive (null) variants. Although both gain-of-function and loss-of-function variants exist, protein function prediction algorithms were essentially better at predicting loss-of-function variants than gain-of-function variants^49^. Thus, we employed a two-sided SKAT, which is good at detecting bidirectional effects, in the analysis of variants for the analysis of the 1^st^ category (all non-synonymous variants). We applied a one-sided CAST to the analyses of variants in the 2^nd^ and 3^rd^ categories (damaging and disruptive). Meta-analyses of the two stages were performed using the MetaSKAT R package for SKAT results and the Metafor R package for CAST results. We set the study-wide significance threshold to 4.6 × 10^−4^, based on a Bonferroni correction for the targeted 36 genes and 3 categories.

To determine the clinical impact of variants in *LDLR* and *PCSK9*, we examined LDL cholesterol levels and onset age of MI in the following groups: carriers of *LDLR* rare variants in each category (all non-synonymous, damaging and disruptive) and carriers of *PCSK9* E32K, R93C and disruptive variants. Effects were estimated using multiple linear regression models adjusted for age, gender, BMI, smoking status and cholesterol lowering medications in assessing LDL cholesterol levels, controlling for the same parameters except for age in assessing the onset age of MI. These statistical calculations were performed using R software.

### Data availability

All data generated or analysed during this study are included in this article (and its Supplementary Information file).

## Electronic supplementary material

Supplementary Information
