# Supplementary material for: Blood lipid-related low-frequency variants in LDLR and PCSK9 are associated with onset age and risk of myocardial infarction in Japanese
Source: Sci Rep. 2018 May 25;8:8107. doi: 10.1038/s41598-018-26453-x (PMC5970143; doi:10.1038/s41598-018-26453-x)
Supplement: Supplementary file 1 — Supplementary Information [file 41598_2018_26453_MOESM1_ESM.docx]

**Supplementary Information**

“Blood lipid-related low-frequency variants in *LDLR* and *PCSK9* are associated with onset age and risk of myocardial infarction in Japanese”

by Tomoyuki Tajima, Hiroyuki Morita, Kaoru Ito, Tsutomu Yamazaki, Michiaki Kubo, Issei Komuro, Yukihide Momozawa

**This file contains 5 Figures and 20 Tables.**

**Figure S1. Gene-based depth of coverage in the discovery stage**

**
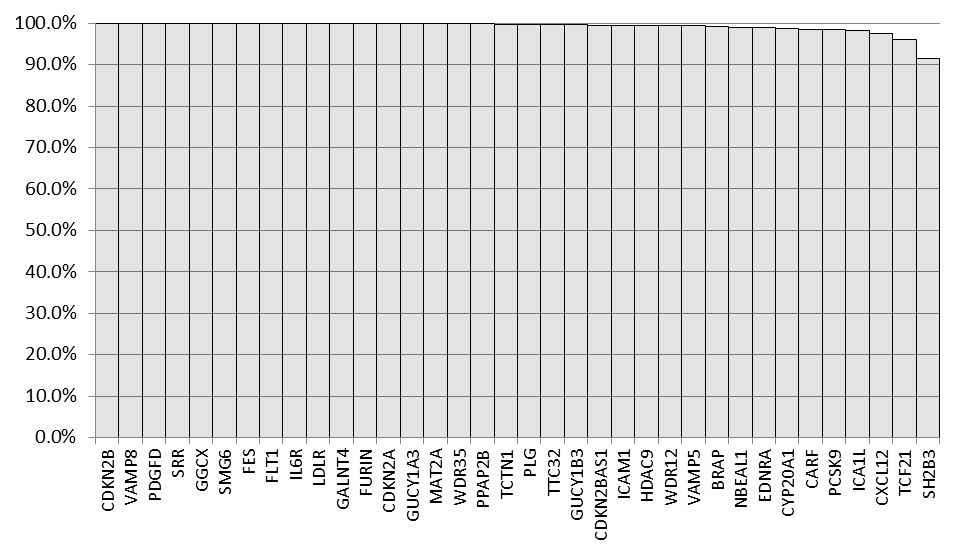
**

**Figure S1: Legend.**

At minimum of 20-fold depth of coverage, 98.9 % of initially targeted bases were covered.

**Figure S2. Gene-based depth of coverage in the replication stage**

**Figure S2: Legend.**

A total of 11 genes were targeted in the replication stage (Stage 2).

At minimum of 20-fold depth of coverage, 98.2 % of targeted bases were covered.

**Figure S3. The effects of *LDLR* and *PCSK9* rare variants without previously-known FH ones on LDL-C levels and onset age of MI.**


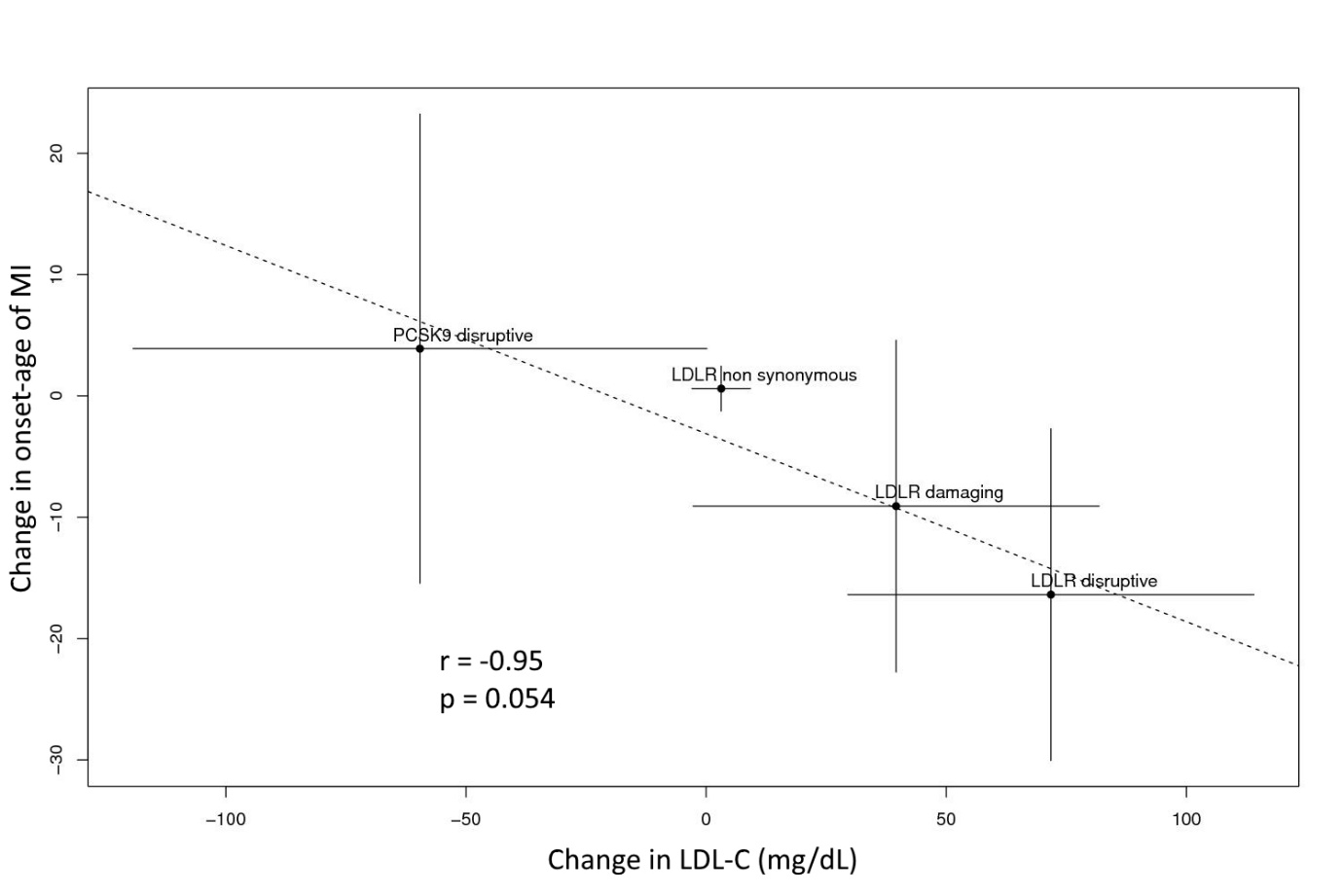


**Figure S3: Legend.**

Dots represent the change from non-carriers of *LDLR* / *PCSK9* rare variants for each group and lines indicate the 95% confidence interval. Abbreviations: r, Pearson’s correlation coefficient.

**Figure S4. The effects of *LDLR* and *PCSK9* rare variants by the administration of cholesterol-lowering drugs on LDL-C levels and onset age of MI.**

**
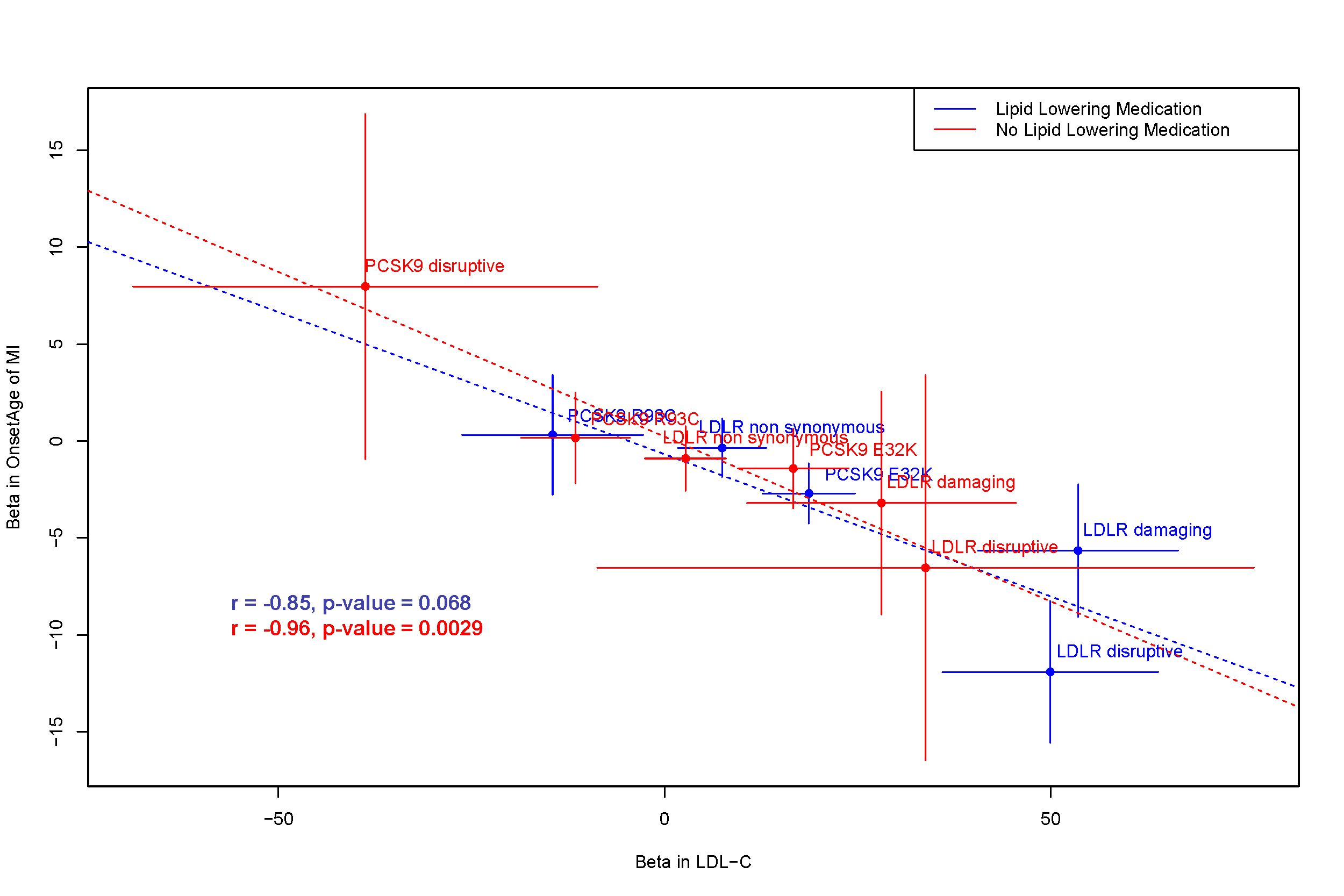
**

**Figure S4: Legend.**

Dots represent the change from non-carriers of *LDLR* / *PCSK9* rare variants for each group and lines indicate the 95% confidence interval. Abbreviations: r, Pearson’s correlation coefficient.

**Figure S5. The effects of *LDLR* and *PCSK9* rare variants by gender on LDL-C levels and onset age of MI.**

**
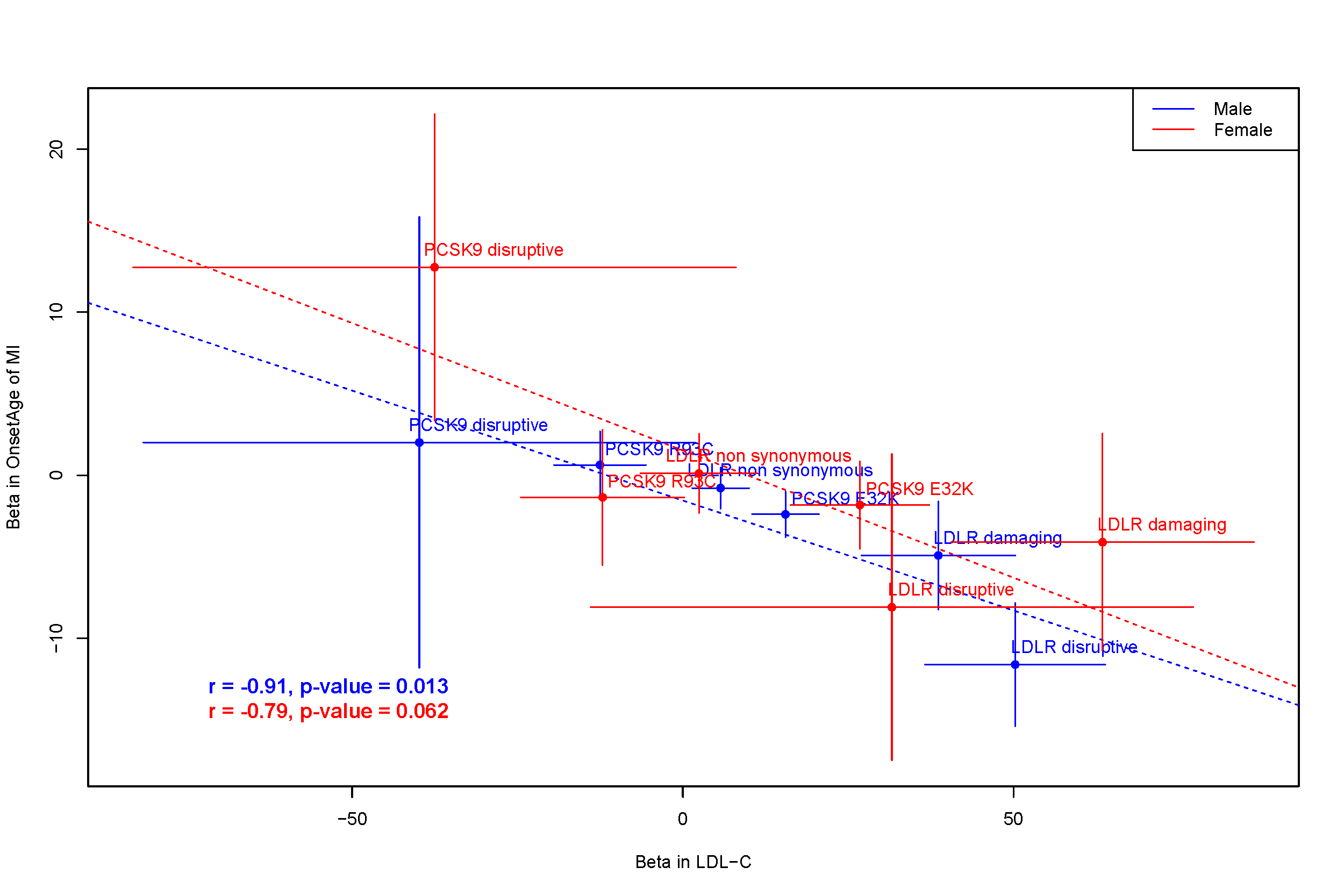
**

**Figure S5: Legend.**

Dots represent the change from non-carriers of *LDLR* / *PCSK9* rare variants for each group and lines indicate the 95% confidence interval. Abbreviations: r, Pearson’s correlation coefficient.

**Table S1. Targeted 36 genes and gene selection criteria.**

| Chr | GWAS SNP | reported genes | Other genes in LD† | eQTL genes‡ | Criteria1 | Criteria2 |
| --- | --- | --- | --- | --- | --- | --- |
| 1 | rs17114036 | *PPAP2B* |  | *PPAP2B* |  | *PPAP2B* |
| 1 | rs4845625 | *IL6R* |  |  |  | *IL6R* |
| 1 | rs11206510 | *PCSK9* |  |  | *PCSK9* |  |
| 2 | rs1561198 | *VAMP5, VAMP8, GGCX* | *MAT2A* |  | *GGCX, MAT2A* | *GGCX, VAMP8* |
| 2 | rs6725887 | *WDR12* | *ICA1L, NBEAL1, CYP20A1, CARF* |  |  | *ICA1L* |
| 2 | rs2123536* | *TTC32, WDR35* |  |  |  | *WDR35* |
| 4 | rs7692387 | *GUCY1A3* | *GUCY1B3* |  | *GUCY1A3, GUCY1B3* | *GUCY1A3* |
| 4 | rs1878406 | *EDNRA* |  |  | *EDNRA* |  |
| 6 | rs4252120 | *PLG* |  |  | *PLG* | *PLG* |
| 6 | rs12190287 | *TCF21* |  |  |  | *TCF21* |
| 7 | rs2023938 | *HDAC9* |  |  | *HDAC9* |  |
| 9 | rs1333049 | *CDKN2BAS1* | *CDKN2A, CDKN2B* |  | *CDKN2A* |  |
| 10 | rs501120 | *CXCL12* |  |  |  | *CXCL12* |
| 11 | rs974819 | *PDGFD* |  |  | *PDGFD* |  |
| 12 | rs3184504 | *SH2B3* | *BRAP* | *MAPKAPK5, TCTN1* | *SH2B3* |  |
| 13 | rs9319428 | *FLT1* |  |  | *FLT1* |  |
| 15 | rs17514846 | *FURIN, FES* |  |  | *FURIN* |  |
| 17 | rs2281727 | *SMG6* |  | *SRR* | *SRR* |  |
| 19 | rs1122608 | *LDLR* |  | *ICAM1* | *ICAM1, LDLR* |  |

^*^The SNP was reported in the genome-wide association study of coronary artery disease in Han Chinese population^7^. Other SNPs were reported by the CARDIOGRAMplusC4D consortium^8^. The loci that scored 0 were not displayed in the table.

† Genes in linkage disequilibrium (*r*^2^ > 0.5) with top SNPs.

‡ Genes which are thought to be causative of CAD according to the studies of eQTL in human vascular endothelial cells^39^.

Criteria 1: there was an established bioassay for measurement of encoded proteins or genes had been known to be “druggable”^40^.

Criteria 2: the gene-deficient mouse models recapitulated CAD/MI-related phenotypes.

Abbreviations: Chr, chromosome; GWAS, genome-wide association study; CAD, coronary artery disease; LD, linkage disequilibrium.

**Table S2. Previously-reported variants identified in the discovery stage**

| CHR | BP | A1 | A2 | Replication | Damaging | Disruptive | dbSNP* | case/control† | LDL-C(SD) | medication(%)‡ | gene |
| --- | --- | --- | --- | --- | --- | --- | --- | --- | --- | --- | --- |
| 1 | 55505520 | A | G | Yes |  |  | rs186669805 | both | 111.9(34.7) | 33.1 | PCSK9 |
| 1 | 55505552 | ACTG | A | Yes |  |  | rs113330492 | both | 110.6(32.5) | 36.1 | PCSK9 |
| 1 | 55505668 | T | C | Yes |  |  | rs11583680 | both | 110.5(32.5) | 36.1 | PCSK9 |
| 1 | 55509585 | T | C | Yes |  |  | rs151193009 | both | 105.3(52.5) | 20.6 | PCSK9 |
| 1 | 55509594 | T | C | Yes |  |  | rs185392267 | case | 85.8(0.0) | 50 | PCSK9 |
| 1 | 55509644 | A | G |  |  |  | rs79805678 | both | 109.4(30.9) | 36 | PCSK9 |
| 1 | 55518036 | T | C |  |  |  | rs200856421 | both | 119.5(34.7) | 25.9 | PCSK9 |
| 1 | 55518370 | T | C |  |  |  | rs7552471 | control | NA | NA | PCSK9 |
| 1 | 55518374 | T | C | Yes |  |  | rs148195424 | control | 135.8(0.0) | NA | PCSK9 |
| 1 | 55518452 | A | G | Yes |  |  | rs200146448 | both | 109.6(35.5) | 37.5 | PCSK9 |
| 1 | 55518456 | T | C | Yes |  |  | rs201789841 | both | 118.2(35.2) | 32 | PCSK9 |
| 1 | 55518467 | G | A |  |  |  | rs2495477 | both | 112.3(32.9) | 40.1 | PCSK9 |
| 1 | 55523033 | A | G |  |  |  | rs509504 | case | NA | NA | PCSK9 |
| 1 | 55523126 | T | C |  |  |  | rs139683719 | control | 118.4(21.5) | 40 | PCSK9 |
| 1 | 55523755 | T | C |  |  |  | rs146924245 | control | NA | NA | PCSK9 |
| 1 | 55524197 | A | G |  |  |  | rs540796 | both | NA | NA | PCSK9 |
| 1 | 55524237 | G | A |  |  |  | rs562556 | both | NA | NA | PCSK9 |
| 1 | 55524303 | T | C |  |  |  | rs374603772 | both | 143.6(0.0) | 100 | PCSK9 |
| 1 | 55524304 | A | G |  |  |  | rs139669564 | case | NA | 100 | PCSK9 |
| 1 | 55524312 | T | C | Yes |  |  | rs201395805 | control | 90.2(9.1) | 14.3 | PCSK9 |
| 1 | 55527093 | T | C | Yes |  |  | rs72646525 | both | 118.1(47.8) | 28.6 | PCSK9 |
| 1 | 55527131 | A | G |  |  |  | rs372586224 | both | 146.3(47.2) | NA | PCSK9 |
| 1 | 55527158 | A | G | Yes |  |  | rs367606156 | both | 95.1(28.8) | 20 | PCSK9 |
| 1 | 55529056 | T | C |  |  |  | rs199815786 | both | 116.5(43.6) | 36.1 | PCSK9 |
| 1 | 55529108 | A | G | Yes |  |  | rs143291739 | both | 118.1(29.6) | 36.8 | PCSK9 |
| 1 | 55529187 | G | A |  |  |  | rs505151 | both | NA | NA | PCSK9 |
| 1 | 56977819 | A | G |  |  |  | rs3738570 | both | 114.1(35.3) | 54.4 | PPAP2B |
| 1 | 56990180 | T | C |  |  |  | rs200383278 | control | NA | NA | PPAP2B |
| 1 | 56990219 | A | G |  |  |  | rs142369156 | both | 108.0(48.9) | 66.7 | PPAP2B |
| 1 | 57002627 | T | C |  |  |  | rs1136164 | both | 109.4(31.2) | 63.2 | PPAP2B |
| 1 | 154401679 | A | G |  |  |  | rs2228144 | both | 112.6(33.9) | 36.4 | IL6R |
| 1 | 154401712 | T | C |  |  |  | rs34099703 | both | 110.8(22.2) | 40 | IL6R |
| 1 | 154407101 | G | A |  |  |  | rs143779412 | both | 98.7(30.0) | 33.3 | IL6R |
| 1 | 154407515 | T | C |  |  |  | rs148521372 | control | NA | NA | IL6R |
| 1 | 154408489 | T | C |  |  |  | rs199999057 | case | 138.1(37.6) | 20 | IL6R |
| 1 | 154426964 | C | T |  |  |  | rs113751503 | both | 127.0(0.0) | NA | IL6R |
| 1 | 154426970 | C | A |  |  |  | rs2228145 | both | 112.5(34.2) | 43.6 | IL6R |
| 1 | 154427026 | A | G |  |  |  | rs201215537 | both | 116.7(24.5) | 34.8 | IL6R |
| 1 | 154427050 | A | G |  |  |  | rs2228146 | control | NA | NA | IL6R |
| 1 | 154437666 | T | C |  |  |  | rs150127972 | control | 139.0(0.0) | NA | IL6R |
| 1 | 154437741 | T | C |  |  |  | rs199661599 | both | 106.4(29.8) | 32.9 | IL6R |
| 2 | 20097748 | C | T |  |  |  | rs74857316 | both | 108.2(30.4) | 54.2 | TTC32 |
| 2 | 20101487 | A | G |  |  |  | rs2304589 | both | 110.1(33.0) | 74.8 | TTC32 |
| 2 | 20113444 | T | C |  |  |  | rs202223893 | control | NA | NA | WDR35 |
| 2 | 20131079 | C | T |  |  |  | rs1191778 | both | 111.5(36.8) | 55.9 | WDR35 |
| 2 | 20132090 | C | T |  | Yes |  | rs75602337 | both | 99.2(9.5) | 50 | WDR35 |
| 2 | 20133221 | T | C |  |  |  | rs2293669 | both | 112.2(46.8) | 39.1 | WDR35 |
| 2 | 20135283 | T | C |  |  |  | rs6741091 | both | NA | NA | WDR35 |
| 2 | 20135333 | A | G |  | Yes |  | rs374027943 | case | 179.6(0.0) | 100 | WDR35 |
| 2 | 20141585 | G | A |  |  |  | rs199882056 | both | 99.2(70.1) | 100 | WDR35 |
| 2 | 20153664 | T | C |  |  |  | rs200385344 | both | 80.4(28.3) | 50 | WDR35 |
| 2 | 20166621 | G | C |  |  |  | rs76623454 | both | 105.8(41.2) | 35.7 | WDR35 |
| 2 | 20166626 | G | A |  |  |  | rs74385826 | both | 114.1(25.7) | 71.4 | WDR35 |
| 2 | 20173339 | A | G |  |  |  | rs185888517 | control | NA | NA | WDR35 |
| 2 | 20173441 | A | G |  |  |  | rs117255034 | both | 108.7(30.8) | 53.2 | WDR35 |
| 2 | 20174240 | C | T |  |  |  | rs139543775 | both | 123.0(0.0) | 40 | WDR35 |
| 2 | 20175312 | A | G |  |  |  | rs34169020 | both | 110.2(31.5) | 60.5 | WDR35 |
| 2 | 20189015 | C | T |  |  |  | rs1060742 | both | 110.9(31.9) | 56.9 | WDR35 |
| 2 | 20189025 | G | C |  |  |  | rs145536838 | both | 109.9(35.5) | 62.9 | WDR35 |
| 2 | 85769047 | G | A |  |  |  | rs75623534 | control | NA | NA | MAT2A |
| 2 | 85769426 | T | C |  |  |  | rs373600407 | control | NA | NA | MAT2A |
| 2 | 85769732 | C | T |  | Yes |  | rs149459768 | both | 123.6(47.4) | 52.2 | MAT2A |
| 2 | 85779552 | A | G |  | Yes |  | rs121909681 | control | NA | NA | GGCX |
| 2 | 85779600 | T | C |  |  |  | rs149078813 | both | 113.7(33.6) | 58.1 | GGCX |
| 2 | 85780107 | A | G |  |  |  | rs10179904 | both | 107.9(32.7) | 55.6 | GGCX |
| 2 | 85780131 | A | G |  |  |  | rs2592551 | both | 111.7(32.8) | 62.2 | GGCX |
| 2 | 85780434 | T | C |  | Yes |  | rs200774203 | control | NA | NA | GGCX |
| 2 | 85780536 | T | C |  |  |  | rs699664 | both | 111.8(32.9) | 62.9 | GGCX |
| 2 | 85780606 | T | C |  |  |  | rs373222495 | both | 97.0(0.0) | NA | GGCX |
| 2 | 85781318 | T | G |  |  |  | rs1254896 | case | NA | NA | GGCX |
| 2 | 85786074 | A | G |  |  |  | rs6751560 | both | 102.2(22.9) | 48 | GGCX |
| 2 | 85788528 | T | C |  |  |  | rs371622780 | case | 158.0(0.0) | NA | GGCX |
| 2 | 85788537 | A | G |  |  |  | rs199867398 | both | 103.2(26.3) | 45.8 | GGCX |
| 2 | 85806238 | A | G |  |  |  | rs368631664 | both | NA | NA | VAMP8 |
| 2 | 85806266 | T | C |  |  |  | rs3731828 | both | 113.3(34.9) | 40.8 | VAMP8 |
| 2 | 85808737 | G | A |  |  |  | rs1009 | both | 112.9(34.5) | 43.4 | VAMP8 |
| 2 | 85818883 | A | G |  |  |  | rs117717889 | both | 109.7(30.4) | 40 | VAMP5 |
| 2 | 85818886 | T | C |  |  |  | rs14976 | both | 108.9(33.8) | 55 | VAMP5 |
| 2 | 85818938 | A | G |  |  |  | rs199574961 | control | NA | NA | VAMP5 |
| 2 | 85818968 | A | G |  |  |  | rs201112273 | control | NA | NA | VAMP5 |
| 2 | 85820146 | A | G |  |  |  | rs201201551 | control | NA | NA | VAMP5 |
| 2 | 85820239 | T | C |  |  |  | rs375941183 | control | NA | NA | VAMP5 |
| 2 | 203747476 | C | A |  |  |  | rs192575551 | both | 102.2(38.2) | 33.3 | WDR12 |
| 2 | 203760910 | C | T |  |  |  | rs148961607 | case | NA | 50 | WDR12 |
| 2 | 203762056 | T | C |  |  |  | rs202163039 | both | NA | NA | WDR12 |
| 2 | 203764337 | A | G |  |  |  | rs147165380 | both | 112.3(24.0) | 50 | WDR12 |
| 2 | 203765754 | T | G |  |  |  | rs199990478 | control | NA | NA | WDR12 |
| 2 | 203765756 | C | T |  |  |  | rs35212307 | both | 106.7(37.0) | 57.9 | WDR12 |
| 2 | 203765784 | C | G |  |  |  | rs201894334 | both | 119.0(0.0) | 66.7 | WDR12 |
| 2 | 203772676 | A | G |  |  |  | rs145877750 | case | 79.0(0.0) | 100 | WDR12 |
| 2 | 203807539 | A | G |  |  |  | rs371549950 | control | NA | NA | CARF |
| 2 | 203818855 | T | C |  |  |  | rs116945957 | both | 109.9(26.4) | 58.3 | CARF |
| 2 | 203818856 | A | G |  |  |  | rs201808511 | control | NA | NA | CARF |
| 2 | 203820439 | C | T |  |  |  | rs146242609 | both | 110.0(37.1) | 56 | CARF |
| 2 | 203825979 | A | G |  | Yes |  | rs375198785 | case | 118.0(0.0) | 100 | CARF |
| 2 | 203836384 | C | T |  |  |  | rs150010762 | both | 125.0(44.7) | 37.5 | CARF |
| 2 | 203839087 | T | C |  |  |  | rs78815956 | control | NA | NA | CARF |
| 2 | 203846817 | T | A |  |  |  | rs72932557 | both | 106.4(37.6) | 62.9 | CARF |
| 2 | 203846865 | T | C |  |  |  | rs75249727 | both | 110.6(29.3) | 58.9 | CARF |
| 2 | 203848307 | CA | C |  | Yes | Yes | rs201520695 | control | NA | NA | CARF |
| 2 | 203974966 | A | G |  |  |  | rs199945717 | both | 128.3(31.6) | 36.7 | NBEAL1 |
| 2 | 203977840 | T | C |  |  |  | rs369275951 | case | 147.0(0.0) | 66.7 | NBEAL1 |
| 2 | 203990773 | C | T | Yes |  |  | rs78750758 | both | 104.3(28.4) | 35.6 | NBEAL1 |
| 2 | 203990789 | G | T |  |  |  | rs72934556 | control | NA | NA | NBEAL1 |
| 2 | 203992579 | T | A |  |  |  | rs185067409 | both | 136.5(89.8) | 36.7 | NBEAL1 |
| 2 | 203995146 | A | G | Yes |  |  | rs376644554 | both | 102.3(33.8) | 33.3 | NBEAL1 |
| 2 | 203996734 | A | G |  |  |  | rs150511473 | control | NA | NA | NBEAL1 |
| 2 | 203996775 | T | C |  |  |  | rs139437459 | control | NA | NA | NBEAL1 |
| 2 | 204000408 | G | A |  |  |  | rs189175469 | both | 94.3(31.0) | 25 | NBEAL1 |
| 2 | 204000571 | G | A |  |  |  | rs138630576 | case | 77.6(0.0) | NA | NBEAL1 |
| 2 | 204000661 | C | A | Yes |  |  | rs201246207 | both | 113.0(32.0) | 52.6 | NBEAL1 |
| 2 | 204000759 | G | A |  |  |  | rs199629992 | case | 109.6(43.0) | 50 | NBEAL1 |
| 2 | 204001377 | A | G |  |  |  | rs201308096 | control | NA | NA | NBEAL1 |
| 2 | 204003431 | C | T | Yes |  |  | rs141142182 | both | 113.6(35.8) | 35.5 | NBEAL1 |
| 2 | 204009369 | G | A | Yes |  |  | rs150123071 | both | 97.8(33.8) | 24.3 | NBEAL1 |
| 2 | 204013747 | T | C |  |  |  | rs201667823 | control | NA | NA | NBEAL1 |
| 2 | 204022478 | T | G | Yes |  |  | rs76455076 | both | 113.7(35.5) | 35 | NBEAL1 |
| 2 | 204048025 | G | A |  |  |  | rs201697451 | control | NA | NA | NBEAL1 |
| 2 | 204062061 | A | G |  |  |  | rs4675323 | both | NA | NA | NBEAL1 |
| 2 | 204066437 | A | G | Yes |  |  | rs201986939 | both | 111.0(28.0) | 35 | NBEAL1 |
| 2 | 204073411 | A | C | Yes | Yes |  | rs200435235 | both | 92.2(0.0) | 33.3 | NBEAL1 |
| 2 | 204073414 | G | A | Yes |  |  | rs140112414 | control | 149.6(0.0) | NA | NBEAL1 |
| 2 | 204073430 | C | G | Yes |  |  | rs369316763 | case | 72.0(0.0) | 50 | NBEAL1 |
| 2 | 204074020 | A | G | Yes |  |  | rs145550746 | both | 110.5(23.4) | 31 | NBEAL1 |
| 2 | 204075770 | T | C |  |  |  | rs370994169 | case | 127.7(18.8) | 66.7 | NBEAL1 |
| 2 | 204075771 | A | G | Yes |  |  | rs200542429 | control | 67.2(0.0) | 100 | NBEAL1 |
| 2 | 204111603 | A | C | Yes |  |  | rs182035689 | both | 111.6(36.7) | 38.8 | CYP20A1 |
| 2 | 204116690 | C | T |  |  |  | rs2043449 | both | NA | NA | CYP20A1 |
| 2 | 204116730 | A | T |  | Yes | Yes | rs201173639 | both | 111.2(29.5) | 38.8 | CYP20A1 |
| 2 | 204116764 | G | A | Yes |  |  | rs200681711 | case | 103.6(0.0) | 20 | CYP20A1 |
| 2 | 204131321 | G | A | Yes |  |  | rs199882728 | both | 122.7(47.1) | NA | CYP20A1 |
| 2 | 204154552 | T | C | Yes |  |  | rs1048013 | both | 113.0(33.6) | 40.6 | CYP20A1 |
| 4 | 148407181 | T | C |  |  |  | rs200498759 | both | 83.6(39.8) | 33.3 | EDNRA |
| 4 | 148441045 | A | G |  |  |  | rs200206289 | control | NA | NA | EDNRA |
| 4 | 148461037 | C | T |  |  |  | rs5333 | both | 111.0(33.4) | 59.8 | EDNRA |
| 4 | 148461073 | A | G |  |  |  | rs5334 | both | 111.0(33.4) | 59.2 | EDNRA |
| 4 | 148461596 | A | G |  | Yes |  | rs201117684 | control | NA | NA | EDNRA |
| 4 | 148461604 | C | T |  |  |  | rs2292764 | both | 114.5(35.4) | 43.5 | EDNRA |
| 4 | 156618149 | A | G |  |  |  | rs17852541 | both | 122.0(0.0) | 100 | GUCY1A3 |
| 4 | 156618222 | A | G |  |  |  | rs148621325 | case | 122.2(0.0) | 100 | GUCY1A3 |
| 4 | 156618228 | G | A |  |  |  | rs372560637 | control | NA | NA | GUCY1A3 |
| 4 | 156631865 | C | G |  |  |  | rs201185726 | both | 98.1(18.1) | 60 | GUCY1A3 |
| 4 | 156632391 | A | G |  |  |  | rs11944673 | case | NA | NA | GUCY1A3 |
| 4 | 156634298 | G | A |  |  |  | rs372081493 | both | 155.8(0.0) | 100 | GUCY1A3 |
| 4 | 156634350 | A | G |  |  |  | rs377442806 | control | NA | NA | GUCY1A3 |
| 4 | 156634422 | A | G |  |  |  | rs139449524 | both | 108.0(0.0) | 50 | GUCY1A3 |
| 4 | 156638325 | T | C |  |  |  | rs112384014 | control | NA | NA | GUCY1A3 |
| 4 | 156643250 | T | C |  | Yes |  | rs377038861 | control | NA | NA | GUCY1A3 |
| 4 | 156651206 | T | C |  |  |  | rs184271525 | both | 97.1(24.2) | 50 | GUCY1A3 |
| 4 | 156651207 | A | G |  |  |  | rs111666522 | control | NA | NA | GUCY1A3 |
| 4 | 156651348 | A | G |  |  |  | rs201558687 | both | 110.4(29.6) | 61.9 | GUCY1A3 |
| 4 | 156696147 | G | A |  |  |  | rs75622942 | both | 112.7(30.9) | 34.8 | GUCY1B3 |
| 4 | 156715173 | G | A | Yes |  |  | rs140646673 | control | 80.0(0.0) | 50 | GUCY1B3 |
| 4 | 156716531 | T | G |  |  |  | rs193112561 | both | 111.9(34.8) | 40.7 | GUCY1B3 |
| 4 | 156721098 | A | G |  |  |  | rs17854507 | both | 114.9(40.2) | 36.6 | GUCY1B3 |
| 4 | 156721101 | T | C |  |  |  | rs201839794 | both | 115.8(32.1) | 32 | GUCY1B3 |
| 4 | 156721198 | T | C |  |  |  | rs2229202 | both | 113.8(36.0) | 35.4 | GUCY1B3 |
| 4 | 156723656 | T | C |  |  |  | rs146343051 | both | 111.5(32.1) | 31.8 | GUCY1B3 |
| 4 | 156723683 | T | C |  |  |  | rs139612573 | both | 113.1(40.1) | 35.3 | GUCY1B3 |
| 6 | 134210598 | G | C |  |  |  | rs61729591 | both | 123.4(15.8) | 50 | TCF21 |
| 6 | 134210600 | T | G |  |  |  | rs56412384 | both | 81.4(8.8) | 50 | TCF21 |
| 6 | 134210629 | C | G |  |  |  | rs74899812 | case | NA | NA | TCF21 |
| 6 | 134210805 | T | C |  | Yes |  | rs3777890 | both | 101.6(15.4) | 58.8 | TCF21 |
| 6 | 161132157 | T | C | Yes |  |  | rs139357983 | case | 69.0(0.0) | 50 | PLG |
| 6 | 161134086 | T | C | Yes |  |  | rs201792453 | both | 113.3(31.1) | 35.9 | PLG |
| 6 | 161135859 | T | A | Yes |  |  | rs150072546 | both | 119.0(27.7) | 28.2 | PLG |
| 6 | 161137719 | T | C |  |  |  | rs200226472 | both | 98.1(27.0) | 66.7 | PLG |
| 6 | 161137779 | C | T |  |  |  | rs14224 | both | 113.2(34.4) | 47.2 | PLG |
| 6 | 161139480 | T | C |  |  |  | rs1130656 | both | 113.2(34.3) | 47 | PLG |
| 6 | 161139798 | A | G | Yes |  |  | rs199771790 | both | 112.4(58.2) | 27.5 | PLG |
| 6 | 161139857 | G | A |  |  |  | rs13231 | control | NA | NA | PLG |
| 6 | 161143463 | T | G | Yes |  |  | rs121918028 | both | 111.5(23.2) | 23.5 | PLG |
| 6 | 161152819 | T | C | Yes |  |  | rs4252128 | both | 107.9(30.7) | 32.5 | PLG |
| 6 | 161152837 | T | C | Yes |  |  | rs140970354 | both | 118.4(40.2) | 40 | PLG |
| 6 | 161155024 | T | C |  |  |  | rs193059713 | both | 114.9(35.0) | 29.6 | PLG |
| 6 | 161159625 | A | G | Yes |  |  | rs121918027 | both | 115.2(32.6) | 38.1 | PLG |
| 6 | 161162406 | C | T |  |  |  | rs4252170 | both | 112.4(32.2) | 36.2 | PLG |
| 6 | 161162407 | A | G | Yes |  |  | rs181030365 | both | 125.4(38.6) | 27.8 | PLG |
| 6 | 161173272 | A | G | Yes |  |  | rs121918033 | both | 109.4(23.9) | 33.3 | PLG |
| 6 | 161173946 | T | G |  |  |  | rs11060 | both | NA | NA | PLG |
| 7 | 18535896 | A | G |  |  |  | rs79608746 | both | 121.4(10.5) | 50 | HDAC9 |
| 7 | 18625016 | G | A |  |  |  | rs199979206 | both | 120.0(23.6) | 25 | HDAC9 |
| 7 | 18625094 | A | G |  |  |  | rs187797966 | both | 124.1(4.1) | 100 | HDAC9 |
| 7 | 18629980 | G | A |  |  |  | rs200990910 | case | 85.7(10.2) | 33.3 | HDAC9 |
| 7 | 18633655 | G | A |  |  |  | rs377204593 | case | 172.6(0.0) | NA | HDAC9 |
| 7 | 18674362 | T | C |  |  |  | rs372266256 | case | NA | 100 | HDAC9 |
| 7 | 18687499 | T | C |  |  |  | rs368603324 | control | NA | NA | HDAC9 |
| 7 | 18767343 | A | G |  |  |  | rs1178127 | both | NA | NA | HDAC9 |
| 7 | 18767359 | T | C |  |  |  | rs199704186 | both | 86.5(26.5) | 50 | HDAC9 |
| 7 | 18993870 | C | T |  |  |  | rs2389998 | both | NA | NA | HDAC9 |
| 9 | 21968727 | T | C |  |  |  | rs189127161 | both | 109.2(28.0) | 38 | CDKN2A |
| 9 | 21968732 | A | G | Yes |  |  | rs181044510 | both | 113.2(16.9) | 31 | CDKN2A |
| 9 | 21970942 | T | C |  |  |  | rs149937815 | both | NA | 25 | CDKN2A |
| 9 | 22008736 | C | G |  |  |  | rs200266212 | both | 114.2(23.4) | 56.5 | CDKN2B |
| 9 | 22029547 | C | T |  | Yes |  | rs564398 | both | 112.8(34.3) | 36.1 | CDKN2BAS1 |
| 9 | 22032685 | G | A |  |  |  | rs144235766 | both | 114.6(34.4) | 39.9 | CDKN2BAS1 |
| 9 | 22032728 | C | T |  |  |  | rs188942730 | both | 112.4(30.8) | 31.9 | CDKN2BAS1 |
| 9 | 22032793 | C | T |  |  |  | rs76521274 | both | 111.9(32.2) | 47.7 | CDKN2BAS1 |
| 9 | 22032841 | C | T |  |  |  | rs80184777 | control | NA | NA | CDKN2BAS1 |
| 9 | 22046870 | T | C |  |  |  | rs76184305 | both | 113.2(32.9) | 37.8 | CDKN2BAS1 |
| 9 | 22049130 | C | G |  |  |  | rs10738605 | both | NA | NA | CDKN2BAS1 |
| 9 | 22056295 | T | C |  | Yes |  | rs7853090 | both | NA | NA | CDKN2BAS1 |
| 9 | 22058581 | G | A |  |  |  | rs78509876 | case | 111.7(15.1) | 50 | CDKN2BAS1 |
| 9 | 22058824 | A | G |  |  |  | rs139035493 | both | 116.0(32.5) | 35.9 | CDKN2BAS1 |
| 9 | 22058911 | T | C |  |  |  | rs374796792 | both | 97.2(25.5) | 33.3 | CDKN2BAS1 |
| 9 | 22058918 | T | C |  |  |  | rs13299593 | control | NA | NA | CDKN2BAS1 |
| 9 | 22059054 | T | G |  |  | Yes | rs117761422 | both | 113.3(33.0) | 38.2 | CDKN2BAS1 |
| 9 | 22063984 | A | G |  |  |  | rs187484661 | case | NA | NA | CDKN2BAS1 |
| 9 | 22063999 | G | C |  |  |  | rs77467777 | both | 113.3(33.0) | 38.1 | CDKN2BAS1 |
| 9 | 22066354 | C | T |  | Yes | Yes | rs117869160 | both | 112.9(32.8) | 34.5 | CDKN2BAS1 |
| 9 | 22096417 | C | T |  |  |  | rs142666561 | both | 106.3(28.7) | 32.7 | CDKN2BAS1 |
| 9 | 22112364 | T | C |  |  |  | rs150657786 | both | 110.8(33.3) | 27.8 | CDKN2BAS1 |
| 9 | 22120236 | G | T |  |  |  | rs138758421 | control | NA | 50 | CDKN2BAS1 |
| 9 | 22120371 | A | G |  |  |  | rs78766516 | both | 113.3(32.7) | 37.9 | CDKN2BAS1 |
| 9 | 22120786 | TATC | T |  |  |  | rs59052189 | both | NA | NA | CDKN2BAS1 |
| 9 | 22120828 | C | A |  |  |  | rs72654278 | both | 113.1(29.6) | 37.5 | CDKN2BAS1 |
| 10 | 44873247 | A | G |  |  |  | rs200184810 | case | 160.3(0.0) | 100 | CXCL12 |
| 10 | 44873344 | A | G |  |  |  | rs375721431 | both | 153.9(24.0) | 57.1 | CXCL12 |
| 10 | 44876240 | A | G |  |  |  | rs201376778 | both | 103.2(0.0) | 50 | CXCL12 |
| 11 | 103780454 | T | C |  |  |  | rs146343067 | case | 68.0(0.0) | 100 | PDGFD |
| 11 | 103780455 | G | A |  |  |  | rs10791649 | both | 112.9(33.6) | 45.7 | PDGFD |
| 11 | 103814191 | T | C | Yes |  |  | rs147536650 | control | 130.0(0.0) | 50 | PDGFD |
| 11 | 103818395 | C | T | Yes |  |  | rs35045740 | both | 115.8(22.4) | 27.3 | PDGFD |
| 12 | 89916811 | T | C |  |  |  | rs2230283 | both | 110.9(31.7) | 59.6 | GALNT4 |
| 12 | 89917037 | A | G |  |  |  | rs2230282 | both | 122.6(37.1) | 54.5 | GALNT4 |
| 12 | 89917349 | T | C |  |  |  | rs200228838 | control | NA | NA | GALNT4 |
| 12 | 89917381 | T | C |  |  |  | rs141839725 | both | 143.3(39.7) | 50 | GALNT4 |
| 12 | 89917437 | T | C |  |  |  | rs184795006 | both | 122.6(0.0) | NA | GALNT4 |
| 12 | 89917518 | A | G |  |  |  | rs2230281 | both | NA | NA | GALNT4 |
| 12 | 89917741 | T | C |  |  |  | rs192512807 | control | NA | NA | GALNT4 |
| 12 | 111057747 | G | A |  |  |  | rs140230455 | both | 112.8(31.5) | 30.8 | TCTN1 |
| 12 | 111066587 | A | C | Yes |  |  | rs117896500 | both | 117.2(32.7) | 33.3 | TCTN1 |
| 12 | 111072476 | A | G |  |  |  | rs200574241 | control | NA | NA | TCTN1 |
| 12 | 111078224 | T | C |  |  |  | rs371899538 | both | 104.9(32.4) | 36.4 | TCTN1 |
| 12 | 111078234 | T | C |  |  |  | rs200460700 | both | 123.2(0.0) | NA | TCTN1 |
| 12 | 111078304 | T | C |  |  |  | rs145970332 | control | 105.0(48.9) | 50 | TCTN1 |
| 12 | 111082823 | A | G |  |  |  | rs377488330 | case | 82.0(0.0) | NA | TCTN1 |
| 12 | 111082836 | T | G | Yes |  |  | rs118096349 | both | 108.3(34.2) | 28.2 | TCTN1 |
| 12 | 111856673 | T | C |  | Yes |  | rs78894077 | both | 112.6(30.6) | 33 | SH2B3 |
| 12 | 111884608 | T | C |  |  |  | rs3184504 | control | NA | NA | SH2B3 |
| 12 | 111885300 | A | G |  |  |  | rs142133709 | control | NA | NA | SH2B3 |
| 12 | 111885507 | T | C |  |  |  | rs143341384 | both | 108.7(29.4) | 33.8 | SH2B3 |
| 12 | 111885526 | A | G |  |  |  | rs199694759 | both | 89.4(11.8) | NA | SH2B3 |
| 12 | 111885615 | T | C |  |  |  | rs371042608 | control | NA | NA | SH2B3 |
| 12 | 111885616 | A | G |  |  |  | rs374482426 | control | NA | NA | SH2B3 |
| 12 | 111885970 | T | C |  |  |  | rs371681526 | both | 109.2(25.7) | 66.7 | SH2B3 |
| 12 | 111885984 | A | G |  |  |  | rs140649197 | both | 113.4(31.8) | 28.2 | SH2B3 |
| 12 | 111886029 | T | C |  |  |  | rs376914049 | both | 102.2(34.5) | 27.3 | SH2B3 |
| 12 | 111886081 | C | T |  | Yes |  | rs199803113 | both | 116.2(50.1) | 24.7 | SH2B3 |
| 12 | 112082176 | T | C |  |  |  | rs376787096 | control | 129.1(31.6) | 50 | BRAP |
| 12 | 112087743 | C | T |  |  |  | rs118186470 | both | 111.6(32.1) | 30.4 | BRAP |
| 12 | 112087805 | T | C |  |  |  | rs61739319 | control | NA | NA | BRAP |
| 12 | 112097057 | T | C |  |  |  | rs151101195 | case | NA | 100 | BRAP |
| 12 | 112097099 | A | G |  |  |  | rs3742002 | both | 113.6(34.4) | 34.3 | BRAP |
| 12 | 112103589 | A | G |  |  |  | rs61999323 | control | NA | NA | BRAP |
| 12 | 112110474 | C | T |  |  |  | rs200808361 | both | 114.7(23.2) | 29.6 | BRAP |
| 12 | 112110489 | C | T |  |  |  | rs3782886 | both | 113.5(32.9) | 41 | BRAP |
| 12 | 112116963 | A | G |  |  |  | rs76800978 | control | 97.2(32.3) | 44.4 | BRAP |
| 12 | 112117009 | G | A |  |  |  | rs61308437 | control | NA | NA | BRAP |
| 12 | 112121068 | A | G |  |  |  | rs80229432 | control | NA | NA | BRAP |
| 13 | 28883061 | G | A |  |  |  | rs7993418 | control | NA | NA | FLT1 |
| 13 | 28891686 | T | C |  | Yes |  | rs143726778 | case | NA | NA | FLT1 |
| 13 | 28893591 | A | G |  |  |  | rs202073140 | control | NA | NA | FLT1 |
| 13 | 28893642 | G | A |  |  |  | rs2296189 | both | 110.8(33.2) | 58.5 | FLT1 |
| 13 | 28895702 | C | T |  |  |  | rs2296191 | both | 104.5(27.5) | 100 | FLT1 |
| 13 | 28896979 | T | C |  |  |  | rs56314249 | both | 107.1(37.4) | 54.1 | FLT1 |
| 13 | 28908193 | T | C |  |  |  | rs61731337 | case | 128.6(0.0) | 100 | FLT1 |
| 13 | 28942715 | T | C |  |  |  | rs183413900 | both | 136.6(38.7) | 25 | FLT1 |
| 13 | 28959130 | C | G |  |  |  | rs115349395 | both | 107.9(56.7) | 50 | FLT1 |
| 13 | 28964198 | T | C |  |  |  | rs17537350 | both | 110.1(30.0) | 54.5 | FLT1 |
| 13 | 29004198 | T | C |  |  |  | rs145266667 | control | NA | NA | FLT1 |
| 13 | 29005429 | T | C |  |  |  | rs372354806 | control | NA | NA | FLT1 |
| 13 | 29012388 | T | C |  |  |  | rs116486684 | both | 192.5(68.6) | NA | FLT1 |
| 13 | 29012441 | T | C |  |  |  | rs55974987 | both | 109.5(26.8) | 40 | FLT1 |
| 13 | 29012466 | A | G |  |  |  | rs2387856 | both | 109.7(35.9) | 56.9 | FLT1 |
| 13 | 29041087 | T | G |  |  |  | rs148901240 | both | 94.6(27.5) | 16.7 | FLT1 |
| 13 | 29041690 | T | C |  |  |  | rs192104849 | both | NA | NA | FLT1 |
| 15 | 91419079 | T | C |  |  |  | rs201172453 | case | 48.8(0.0) | 100 | FURIN |
| 15 | 91419098 | T | C |  |  |  | rs16944971 | both | 112.7(29.0) | 14.3 | FURIN |
| 15 | 91419548 | T | C |  |  |  | rs148110342 | both | 108.3(19.4) | 66.7 | FURIN |
| 15 | 91419553 | A | G |  |  |  | rs372383658 | case | 98.0(0.0) | 100 | FURIN |
| 15 | 91419740 | A | G |  |  |  | rs116359616 | control | NA | NA | FURIN |
| 15 | 91421429 | T | C |  |  |  | rs191128549 | both | 118.4(41.2) | 50 | FURIN |
| 15 | 91421438 | T | C |  |  |  | rs183632050 | both | 106.4(37.3) | 52.4 | FURIN |
| 15 | 91422966 | G | A |  |  |  | rs147904503 | case | NA | NA | FURIN |
| 15 | 91423337 | T | C |  |  |  | rs150925934 | case | 84.0(61.7) | 100 | FURIN |
| 15 | 91423338 | A | G |  |  |  | rs377009422 | case | 171.0(0.0) | 100 | FURIN |
| 15 | 91423381 | T | C |  |  |  | rs117131791 | both | 98.3(9.5) | 80 | FURIN |
| 15 | 91423405 | T | C |  |  |  | rs376410361 | control | NA | NA | FURIN |
| 15 | 91423423 | T | C |  |  |  | rs74543508 | case | 198.1(0.0) | 100 | FURIN |
| 15 | 91423937 | A | G |  |  |  | rs202096241 | case | 77.0(0.0) | 100 | FURIN |
| 15 | 91424029 | T | C |  |  |  | rs142489043 | both | 140.6(3.7) | 50 | FURIN |
| 15 | 91424215 | T | C |  |  |  | rs146566561 | case | 113.6(0.0) | 50 | FURIN |
| 15 | 91424255 | G | A |  |  |  | rs199904269 | both | 96.2(43.4) | 35 | FURIN |
| 15 | 91424574 | C | G |  |  |  | rs6226 | both | 111.5(33.6) | 76.5 | FURIN |
| 15 | 91424658 | T | C |  |  |  | rs373456882 | case | NA | NA | FURIN |
| 15 | 91424833 | T | C |  | Yes |  | rs148060996 | both | 165.2(0.0) | NA | FURIN |
| 15 | 91424896 | G | A |  |  |  | rs202152215 | both | 81.2(6.8) | 100 | FURIN |
| 15 | 91424929 | T | C |  |  |  | rs201142022 | both | 82.8(0.0) | 100 | FURIN |
| 15 | 91424957 | A | G |  |  |  | rs35641241 | control | NA | NA | FURIN |
| 15 | 91425038 | T | C |  |  |  | rs146218928 | both | 105.5(23.9) | 100 | FURIN |
| 15 | 91428290 | C | T |  |  |  | rs11539637 | both | NA | NA | FES |
| 15 | 91428681 | T | C |  |  |  | rs56041861 | case | 125.2(0.0) | NA | FES |
| 15 | 91430208 | C | T |  |  |  | rs187616533 | control | NA | NA | FES |
| 15 | 91430436 | T | C |  |  |  | rs2071383 | both | NA | NA | FES |
| 15 | 91432603 | T | C |  |  |  | rs141047785 | both | 104.3(54.2) | 66.7 | FES |
| 15 | 91433123 | A | G |  |  |  | rs144946287 | both | NA | 50 | FES |
| 15 | 91433190 | A | G |  |  |  | rs183173261 | both | 117.3(27.2) | 44.8 | FES |
| 15 | 91433663 | A | G |  |  |  | rs143824045 | both | 140.8(0.0) | 50 | FES |
| 15 | 91433701 | A | G |  |  |  | rs139750858 | control | NA | NA | FES |
| 15 | 91434277 | T | C |  |  |  | rs2229074 | control | NA | NA | FES |
| 15 | 91436360 | T | C |  |  |  | rs147098559 | both | 117.0(22.7) | 42.9 | FES |
| 15 | 91436965 | T | C |  |  |  | rs2227989 | control | NA | NA | FES |
| 15 | 91438783 | T | C |  |  |  | rs202150302 | case | 154.8(0.0) | NA | FES |
| 17 | 1964859 | T | C | Yes | Yes |  | rs369323853 | control | 122.9(14.2) | NA | SMG6 |
| 17 | 1968967 | C | T | Yes |  |  | rs2232487 | control | 119.4(22.1) | 100 | SMG6 |
| 17 | 1972116 | T | C | Yes |  |  | rs2232483 | case | 84.4(16.1) | 100 | SMG6 |
| 17 | 1972209 | C | T | Yes |  |  | rs2273980 | both | 112.5(32.8) | 34.2 | SMG6 |
| 17 | 1985205 | T | C | Yes |  |  | rs376160722 | both | 140.2(26.3) | 25 | SMG6 |
| 17 | 1989027 | G | C | Yes |  |  | rs187319098 | both | 108.3(28.1) | 36.4 | SMG6 |
| 17 | 2076064 | A | G |  |  |  | rs370985738 | control | NA | NA | SMG6 |
| 17 | 2076084 | T | C |  |  |  | rs191617249 | both | 105.9(26.2) | 29.4 | SMG6 |
| 17 | 2091721 | A | G |  |  |  | rs200765278 | both | 108.5(35.2) | 34.4 | SMG6 |
| 17 | 2091765 | T | C | Yes |  |  | rs903160 | both | 112.0(34.6) | 40.5 | SMG6 |
| 17 | 2139843 | A | G | Yes | Yes |  | rs376512734 | case | 114.8(7.6) | 33.3 | SMG6 |
| 17 | 2147998 | C | T | Yes |  |  | rs148043002 | both | 118.9(31.2) | 30 | SMG6 |
| 17 | 2185956 | T | G | Yes |  |  | rs191133452 | both | 94.1(35.0) | 33.3 | SMG6 |
| 17 | 2186100 | T | C |  |  |  | rs749240 | both | 112.0(34.3) | 39.4 | SMG6 |
| 17 | 2186125 | A | T | Yes |  |  | rs142250229 | both | 108.7(24.7) | 50 | SMG6 |
| 17 | 2186947 | G | A | Yes | Yes |  | rs372239404 | both | 126.4(52.6) | 25 | SMG6 |
| 17 | 2187015 | A | G |  |  |  | rs142112792 | both | 104.0(27.1) | 26.7 | SMG6 |
| 17 | 2202240 | A | G |  | Yes |  | rs139239231 | control | NA | NA | SMG6 |
| 17 | 2202635 | A | G | Yes |  |  | rs145169086 | both | 94.5(33.4) | 60 | SMG6 |
| 17 | 2202766 | A | G |  |  |  | rs139116937 | both | 113.0(38.8) | 36.4 | SMG6 |
| 17 | 2202943 | T | C |  |  |  | rs216196 | both | NA | NA | SMG6 |
| 17 | 2203025 | G | T | Yes |  |  | rs1885987 | both | 113.2(34.7) | 36.9 | SMG6 |
| 17 | 2203057 | C | G | Yes |  |  | rs200020527 | both | 113.0(34.1) | 37.5 | SMG6 |
| 17 | 2203071 | T | G | Yes |  |  | rs149069908 | both | 108.0(31.1) | 39.1 | SMG6 |
| 17 | 2203167 | T | G |  |  |  | rs216195 | both | NA | NA | SMG6 |
| 17 | 2203175 | G | C | Yes |  |  | rs1885986 | both | 113.4(34.0) | 36.3 | SMG6 |
| 17 | 2203209 | A | G |  |  |  | rs371345349 | control | NA | NA | SMG6 |
| 17 | 2203252 | A | G |  |  |  | rs201836751 | both | 107.3(31.0) | 38.8 | SMG6 |
| 17 | 2203348 | T | C |  |  |  | rs216194 | control | NA | NA | SMG6 |
| 17 | 2203349 | A | G | Yes |  |  | rs151096972 | control | 124.0(0.0) | NA | SMG6 |
| 17 | 2203453 | A | G |  |  |  | rs216193 | both | NA | NA | SMG6 |
| 17 | 2203563 | A | G |  |  |  | rs200691961 | control | NA | NA | SMG6 |
| 17 | 2203795 | A | G |  |  |  | rs202139783 | both | 115.4(31.9) | 36 | SMG6 |
| 17 | 2203845 | A | G | Yes |  |  | rs202048947 | both | 101.0(62.9) | 62.5 | SMG6 |
| 17 | 2203875 | A | G |  | Yes |  | rs374509840 | case | 122.2(0.0) | 100 | SMG6 |
| 17 | 2227081 | G | A |  | Yes |  | rs200376855 | control | NA | NA | SRR |
| 19 | 10385599 | T | C |  |  |  | rs146780348 | both | 49.0(0.0) | 50 | ICAM1 |
| 19 | 10394763 | T | C |  |  |  | rs375023616 | control | NA | NA | ICAM1 |
| 19 | 10395248 | T | C |  |  |  | rs13306429 | both | 113.8(31.5) | 55.2 | ICAM1 |
| 19 | 10395252 | T | C |  |  |  | rs139178890 | control | NA | NA | ICAM1 |
| 19 | 10395308 | T | C |  |  |  | rs2071441 | both | 87.6(46.9) | 80 | ICAM1 |
| 19 | 10395468 | A | G |  |  |  | rs5497 | case | 158.6(0.0) | NA | ICAM1 |
| 19 | 10395489 | T | C |  |  |  | rs112301533 | control | NA | NA | ICAM1 |
| 19 | 10395501 | T | C |  |  |  | rs371463487 | both | 88.2(0.0) | 100 | ICAM1 |
| 19 | 10395520 | A | G |  |  |  | rs199931094 | both | 92.5(41.7) | 66.7 | ICAM1 |
| 19 | 10395624 | A | G |  |  |  | rs13306430 | both | 128.3(47.4) | 50 | ICAM1 |
| 19 | 10395683 | G | A |  |  |  | rs5498 | both | 111.4(32.5) | 69.7 | ICAM1 |
| 19 | 10395883 | T | C |  |  |  | rs202205823 | control | NA | NA | ICAM1 |
| 19 | 11200282 | A | G |  |  |  | rs147509697 | control | NA | NA | LDLR |
| 19 | 11210912 | T | C |  |  |  | rs2228671 | both | 104.2(26.5) | 49.1 | LDLR |
| 19 | 11210921 | T | C |  |  |  | rs72658855 | control | NA | NA | LDLR |
| 19 | 11210978 | T | C |  |  |  | rs138078086 | case | NA | NA | LDLR |
| 19 | 11215926 | A | G | Yes |  |  | rs201102461 | both | 113.4(35.6) | 32.6 | LDLR |
| 19 | 11216233 | C | T |  |  |  | rs201374693 | control | NA | NA | LDLR |
| 19 | 11218157 | T | C | Yes |  |  | rs151207122 | control | 184.8(0.0) | 100 | LDLR |
| 19 | 11218189 | T | C |  |  |  | rs13306512 | both | 105.3(37.5) | 34 | LDLR |
| 19 | 11221356 | T | C |  |  |  | rs199622547 | both | 109.8(32.5) | 44.4 | LDLR |
| 19 | 11221357 | A | G | Yes |  |  | rs72658860 | control | 95.2(0.0) | NA | LDLR |
| 19 | 11221443 | T | C |  |  |  | rs13306515 | both | 115.2(33.4) | 37.3 | LDLR |
| 19 | 11223961 | T | C |  |  |  | rs13306498 | both | 113.0(35.4) | 34.7 | LDLR |
| 19 | 11224233 | A | G | Yes |  |  | rs193922568 | control | 116.0(0.0) | NA | LDLR |
| 19 | 11224265 | A | G |  |  |  | rs5930 | both | NA | NA | LDLR |
| 19 | 11224326 | A | G |  | Yes |  | rs373646964 | case | NA | 100 | LDLR |
| 19 | 11224368 | A | G | Yes |  |  | rs373848925 | case | 119.5(12.6) | 50 | LDLR |
| 19 | 11224382 | A | G |  |  |  | rs367655096 | both | 118.2(29.1) | 18.8 | LDLR |
| 19 | 11224398 | A | G | Yes |  |  | rs141673997 | case | 117.1(30.2) | 75 | LDLR |
| 19 | 11226800 | T | C |  |  |  | rs5929 | both | 112.9(33.9) | 41 | LDLR |
| 19 | 11227554 | T | C |  |  |  | rs1799898 | both | 116.3(34.3) | 33 | LDLR |
| 19 | 11227602 | T | C |  |  |  | rs688 | both | 114.7(32.8) | 38 | LDLR |
| 19 | 11227612 | T | C | Yes | Yes |  | rs373371572 | case | 134.9(24.4) | 42.9 | LDLR |
| 19 | 11230842 | T | C |  |  |  | rs5926 | control | NA | NA | LDLR |
| 19 | 11230881 | C | T |  |  |  | rs5925 | both | 113.9(34.2) | 38.7 | LDLR |
| 19 | 11231112 | T | C | Yes | Yes |  | rs28942084 | case | 250.4(0.0) | 33.3 | LDLR |
| 19 | 11233941 | A | G |  |  |  | rs5927 | both | NA | NA | LDLR |
| 19 | 11238691 | T | C |  |  |  | rs377563758 | control | 77.9(64.3) | 100 | LDLR |
| 19 | 11238695 | A | G | Yes |  |  | rs199766976 | control | 136.6(0.0) | NA | LDLR |
| 19 | 11238730 | T | C |  |  |  | rs183255090 | both | 125.1(33.6) | 39.4 | LDLR |
| 19 | 11238731 | A | G |  |  |  | rs143771219 | control | NA | NA | LDLR |
| 19 | 11240240 | A | G |  |  |  | rs5928 | case | 77.8(0.0) | NA | LDLR |
| 19 | 11240328 | T | C |  |  |  | rs374635484 | case | NA | 100 | LDLR |
| 19 | 11241988 | T | C | Yes |  |  | rs13306505 | both | 104.6(33.3) | 29.2 | LDLR |

We listed 395 previously-known variants identified in the discovery stage. *Recorded in dbSNP (138) †Variants were case-specific, control-specific or identified in both groups. ‡A percentage of the administration of cholesterol lowering medications among variants carriers. Abbreviations: Chr, chromosome; A1, minor allele; A2, major allele; NA, data not available

**Table S3. Novel variants identified in the discovery stage.**

| CHR | BP | A1 | A2 | Replication* | Damaging | Disruptive | Confirmation† | case/control‡ | LDL-C(SD) | medication(%)§ | gene |
| --- | --- | --- | --- | --- | --- | --- | --- | --- | --- | --- | --- |
| 1 | 55505555 | C | G |  |  |  |  | control | 96.4(16.5) | NA | PCSK9 |
| 1 | 55505604 | A | G | Yes |  |  |  | both | 125.4(41.4) | 49.2 | PCSK9 |
| 1 | 55505671 | C | A |  |  |  |  | both | 96.2(0.0) | 33.3 | PCSK9 |
| 1 | 55505712 | A | G |  |  |  |  | both | 97.6(27.0) | 40 | PCSK9 |
| 1 | 55509520 | T | C | Yes |  |  |  | control | NA | NA | PCSK9 |
| 1 | 55509543 | A | G | Yes |  |  |  | control | NA | NA | PCSK9 |
| 1 | 55509561 | A | G | Yes |  |  |  | case | 118.7(11.1) | 60 | PCSK9 |
| 1 | 55509606 | C | G |  |  |  |  | control | NA | NA | PCSK9 |
| 1 | 55509630 | T | C |  |  |  |  | case | 173.4(0.0) | 100 | PCSK9 |
| 1 | 55509704 | C | G |  |  |  |  | case | 100.0(32.5) | 50 | PCSK9 |
| 1 | 55512299 | T | C | Yes |  |  |  | both | 140.0(34.3) | 11.1 | PCSK9 |
| 1 | 55518016 | A | G |  |  |  |  | control | NA | NA | PCSK9 |
| 1 | 55518029 | G | T |  |  |  |  | control | NA | NA | PCSK9 |
| 1 | 55518063 | T | C |  |  |  |  | both | 112.3(36.7) | 33.3 | PCSK9 |
| 1 | 55518070 | T | C |  | Yes |  | IGV snapshot | both | NA | NA | PCSK9 |
| 1 | 55518082 | G | C | Yes |  |  |  | both | 100.7(55.8) | 20 | PCSK9 |
| 1 | 55518381 | A | C |  | Yes |  | IGV snapshot | control | NA | NA | PCSK9 |
| 1 | 55518383 | A | G |  | Yes |  | IGV snapshot | control | NA | NA | PCSK9 |
| 1 | 55518386 | C | G |  | Yes |  | IGV snapshot | control | NA | NA | PCSK9 |
| 1 | 55518407 | T | C |  |  |  |  | control | NA | NA | PCSK9 |
| 1 | 55521783 | T | G |  |  |  |  | control | NA | NA | PCSK9 |
| 1 | 55523188 | A | G |  | Yes | Yes | IGV snapshot | case | 4.8(0.0) | NA | PCSK9 |
| 1 | 55523798 | G | A | Yes |  |  |  | both | 106.3(29.4) | 24.6 | PCSK9 |
| 1 | 55523808 | A | C |  |  |  |  | case | 131.8(0.0) | NA | PCSK9 |
| 1 | 55523812 | A | G |  | Yes | Yes | IGV snapshot | both | 91.6(22.5) | 20 | PCSK9 |
| 1 | 55523866 | A | C |  |  |  |  | case | NA | NA | PCSK9 |
| 1 | 55524246 | A | T |  | Yes |  | IGV snapshot | case | 126.8(0.0) | NA | PCSK9 |
| 1 | 55524300 | T | C | Yes |  |  |  | control | NA | NA | PCSK9 |
| 1 | 55525195 | A | G | Yes |  |  |  | case | 104.5(12.9) | 55.6 | PCSK9 |
| 1 | 55525219 | A | G |  | Yes |  | IGV snapshot | case | 125.2(57.1) | 16.7 | PCSK9 |
| 1 | 55527059 | T | C |  |  |  |  | both | 218.0(0.0) | NA | PCSK9 |
| 1 | 55527204 | C | A |  |  |  |  | control | NA | NA | PCSK9 |
| 1 | 55529064 | A | G | Yes | Yes |  | IGV snapshot | both | 123.7(47.0) | 23.1 | PCSK9 |
| 1 | 55529125 | T | C |  |  |  |  | control | 118.9(29.8) | 33.3 | PCSK9 |
| 1 | 55529153 | G | C | Yes |  |  |  | both | 102.7(36.4) | 37.5 | PCSK9 |
| 1 | 55529164 | T | C |  |  |  |  | case | NA | NA | PCSK9 |
| 1 | 55529182 | A | C | Yes |  |  |  | both | 114.2(32.8) | 70 | PCSK9 |
| 1 | 55529217 | A | G |  |  |  |  | control | NA | NA | PCSK9 |
| 1 | 56962228 | G | T |  |  |  |  | case | 112.0(0.0) | NA | PPAP2B |
| 1 | 56962281 | C | T |  |  |  |  | case | 69.0(0.0) | 100 | PPAP2B |
| 1 | 56962284 | T | C |  |  |  |  | case | 90.8(0.0) | 100 | PPAP2B |
| 1 | 56962296 | C | G |  |  |  |  | case | 112.0(0.0) | 100 | PPAP2B |
| 1 | 56977789 | C | G |  |  |  |  | control | NA | NA | PPAP2B |
| 1 | 56977821 | G | A |  | Yes |  |  | both | 121.4(0.0) | NA | PPAP2B |
| 1 | 56989506 | T | G |  |  |  |  | both | 84.4(19.2) | NA | PPAP2B |
| 1 | 56990007 | T | C |  |  |  |  | control | NA | NA | PPAP2B |
| 1 | 56990157 | T | C |  |  |  |  | case | NA | NA | PPAP2B |
| 1 | 56990168 | C | T |  |  |  |  | case | NA | NA | PPAP2B |
| 1 | 56990192 | G | A |  |  |  |  | control | NA | NA | PPAP2B |
| 1 | 57002630 | A | G |  |  |  |  | both | 66.0(0.0) | 100 | PPAP2B |
| 1 | 57002641 | C | T |  |  |  |  | both | 138.8(0.0) | 50 | PPAP2B |
| 1 | 57002719 | C | T |  |  |  |  | control | NA | NA | PPAP2B |
| 1 | 57002777 | A | G |  |  |  |  | both | 175.0(0.0) | 100 | PPAP2B |
| 1 | 57044612 | T | C |  |  |  |  | case | 184.0(0.0) | 100 | PPAP2B |
| 1 | 57044639 | A | G |  |  |  |  | control | NA | NA | PPAP2B |
| 1 | 57044656 | A | G |  |  |  |  | case | 95.4(0.0) | NA | PPAP2B |
| 1 | 154378155 | C | G |  |  |  |  | case | NA | 100 | IL6R |
| 1 | 154378156 | G | A |  |  |  |  | control | NA | NA | IL6R |
| 1 | 154401686 | A | G |  |  |  |  | case | 89.3(31.2) | 33.3 | IL6R |
| 1 | 154401713 | A | G |  |  |  |  | case | 96.2(16.7) | 60 | IL6R |
| 1 | 154401736 | C | A |  |  |  |  | control | NA | NA | IL6R |
| 1 | 154401804 | C | G |  |  |  |  | both | 140.5(43.1) | 50 | IL6R |
| 1 | 154401840 | T | C |  |  |  |  | both | 108.3(26.7) | 33.3 | IL6R |
| 1 | 154401879 | C | G |  |  |  |  | both | 87.4(0.0) | 100 | IL6R |
| 1 | 154401883 | T | C |  |  |  |  | case | 140.9(31.4) | NA | IL6R |
| 1 | 154401897 | C | G |  |  |  |  | case | 140.8(49.2) | 25 | IL6R |
| 1 | 154406996 | T | C |  | Yes | Yes |  | control | NA | NA | IL6R |
| 1 | 154407004 | A | T |  |  |  |  | control | NA | NA | IL6R |
| 1 | 154407010 | T | C |  |  |  |  | both | 50.3(43.5) | 40 | IL6R |
| 1 | 154407081 | C | A |  |  |  |  | both | 109.3(48.9) | 42.9 | IL6R |
| 1 | 154407127 | C | T |  |  |  |  | case | 71.0(0.0) | 100 | IL6R |
| 1 | 154407154 | G | A |  |  |  |  | case | 71.0(0.0) | 100 | IL6R |
| 1 | 154407483 | GC | G |  | Yes | Yes |  | case | 151.1(51.6) | 20 | IL6R |
| 1 | 154407503 | T | C |  |  |  |  | both | NA | NA | IL6R |
| 1 | 154407593 | G | A |  |  |  |  | both | 107.5(33.0) | 38.5 | IL6R |
| 1 | 154407597 | T | C |  |  |  |  | case | 107.0(32.2) | NA | IL6R |
| 1 | 154407620 | T | C |  |  |  |  | control | NA | NA | IL6R |
| 1 | 154408450 | A | G |  |  |  |  | control | 147.7(43.2) | 75 | IL6R |
| 1 | 154408477 | T | C |  |  |  |  | control | NA | NA | IL6R |
| 1 | 154408507 | T | G |  |  |  |  | case | 135.5(35.0) | 66.7 | IL6R |
| 1 | 154408515 | A | G |  | Yes |  |  | both | 142.7(34.8) | 40 | IL6R |
| 1 | 154408569 | C | T |  |  |  |  | both | 113.6(31.1) | 26.9 | IL6R |
| 1 | 154408576 | A | G |  |  |  |  | case | 107.6(0.0) | NA | IL6R |
| 1 | 154420644 | A | G |  |  |  |  | control | NA | NA | IL6R |
| 1 | 154422407 | T | C |  |  |  |  | case | NA | 100 | IL6R |
| 1 | 154437628 | A | G |  |  |  |  | control | 115.2(0.0) | 100 | IL6R |
| 1 | 154437635 | A | G |  |  |  |  | control | NA | NA | IL6R |
| 1 | 154437711 | T | C |  |  |  |  | both | 138.2(32.6) | 45.5 | IL6R |
| 1 | 154437789 | A | G |  |  |  |  | case | 88.3(0.0) | 100 | IL6R |
| 2 | 20096749 | G | C |  | Yes | Yes |  | both | 119.8(0.0) | 100 | TTC32 |
| 2 | 20096773 | C | CTTT |  |  |  |  | control | NA | NA | TTC32 |
| 2 | 20096852 | C | CCTT |  |  |  |  | case | 151.0(0.0) | 100 | TTC32 |
| 2 | 20096863 | C | T |  | Yes |  |  | case | 104.9(26.3) | NA | TTC32 |
| 2 | 20097664 | C | T |  |  |  |  | both | 96.0(0.0) | 100 | TTC32 |
| 2 | 20097732 | T | C |  |  |  |  | control | NA | NA | TTC32 |
| 2 | 20097754 | T | C |  |  |  |  | case | 97.0(2.8) | NA | TTC32 |
| 2 | 20097769 | T | A |  |  |  |  | control | NA | NA | TTC32 |
| 2 | 20101477 | G | T |  |  |  |  | control | NA | NA | TTC32 |
| 2 | 20101543 | G | C |  |  |  |  | case | 99.0(0.0) | 100 | TTC32 |
| 2 | 20113400 | A | G |  |  |  |  | case | 218.2(0.0) | 100 | WDR35 |
| 2 | 20113426 | C | T |  |  |  |  | case | 161.2(0.0) | NA | WDR35 |
| 2 | 20113796 | A | ACC |  | Yes | Yes |  | case | 91.2(0.0) | NA | WDR35 |
| 2 | 20113835 | A | C |  | Yes |  |  | control | NA | NA | WDR35 |
| 2 | 20113844 | C | T |  |  |  |  | control | NA | NA | WDR35 |
| 2 | 20113979 | T | C |  |  |  |  | case | NA | 100 | WDR35 |
| 2 | 20114030 | T | G |  | Yes |  |  | case | 81.0(0.0) | NA | WDR35 |
| 2 | 20130294 | G | C |  |  |  |  | control | NA | NA | WDR35 |
| 2 | 20131094 | G | A |  |  |  |  | control | NA | NA | WDR35 |
| 2 | 20131132 | C | T |  |  |  |  | control | NA | NA | WDR35 |
| 2 | 20131170 | C | T |  |  |  |  | case | NA | 100 | WDR35 |
| 2 | 20132045 | G | T |  |  |  |  | control | NA | NA | WDR35 |
| 2 | 20132084 | C | T |  |  |  |  | control | NA | NA | WDR35 |
| 2 | 20132177 | C | T |  |  |  |  | control | NA | NA | WDR35 |
| 2 | 20133177 | A | G |  |  |  |  | control | NA | NA | WDR35 |
| 2 | 20133195 | T | C |  |  |  |  | control | NA | NA | WDR35 |
| 2 | 20133247 | G | A |  |  |  |  | control | NA | NA | WDR35 |
| 2 | 20133266 | T | C |  |  |  |  | case | NA | NA | WDR35 |
| 2 | 20135246 | A | G |  |  |  |  | control | NA | NA | WDR35 |
| 2 | 20135997 | G | A |  |  |  |  | control | NA | NA | WDR35 |
| 2 | 20136014 | T | C |  |  |  |  | case | 157.0(0.0) | NA | WDR35 |
| 2 | 20137504 | T | C |  |  |  |  | control | NA | NA | WDR35 |
| 2 | 20137515 | T | C |  |  |  |  | control | NA | NA | WDR35 |
| 2 | 20137518 | A | G |  |  |  |  | control | NA | NA | WDR35 |
| 2 | 20137524 | A | C |  |  |  |  | both | 133.7(28.7) | 100 | WDR35 |
| 2 | 20137548 | A | G |  |  |  |  | control | NA | NA | WDR35 |
| 2 | 20137550 | T | C |  |  |  |  | case | 164.4(0.0) | NA | WDR35 |
| 2 | 20137567 | C | T |  | Yes |  |  | case | 81.8(0.0) | 100 | WDR35 |
| 2 | 20137578 | A | C |  |  |  |  | both | 141.6(45.0) | 100 | WDR35 |
| 2 | 20137643 | A | G |  |  |  |  | case | NA | 100 | WDR35 |
| 2 | 20137666 | C | T |  |  |  |  | control | NA | NA | WDR35 |
| 2 | 20137669 | A | AG |  | Yes | Yes |  | both | NA | NA | WDR35 |
| 2 | 20137679 | C | T |  |  |  |  | control | NA | NA | WDR35 |
| 2 | 20138054 | C | T |  |  |  |  | case | NA | 50 | WDR35 |
| 2 | 20138102 | C | T |  |  |  |  | both | 95.7(32.9) | 60 | WDR35 |
| 2 | 20138117 | A | G |  | Yes |  |  | case | 152.8(0.0) | 100 | WDR35 |
| 2 | 20141539 | T | A |  |  |  |  | both | 109.3(36.5) | 66.7 | WDR35 |
| 2 | 20145712 | A | G |  |  |  |  | case | NA | NA | WDR35 |
| 2 | 20146328 | GACCCT | G |  | Yes | Yes |  | case | NA | 100 | WDR35 |
| 2 | 20147954 | C | T |  |  |  |  | both | 118.4(40.7) | 50 | WDR35 |
| 2 | 20151219 | A | G |  |  |  |  | control | NA | NA | WDR35 |
| 2 | 20153620 | A | G |  |  |  |  | control | NA | NA | WDR35 |
| 2 | 20153667 | C | T |  | Yes |  |  | control | NA | NA | WDR35 |
| 2 | 20153693 | T | C |  |  |  |  | control | NA | NA | WDR35 |
| 2 | 20153735 | C | T |  |  |  |  | case | 81.8(0.0) | 100 | WDR35 |
| 2 | 20160318 | C | T |  |  |  |  | control | NA | NA | WDR35 |
| 2 | 20160351 | C | T |  |  |  |  | both | 11.0(0.0) | 50 | WDR35 |
| 2 | 20160370 | C | T |  |  |  |  | control | NA | NA | WDR35 |
| 2 | 20160373 | C | A |  |  |  |  | control | NA | NA | WDR35 |
| 2 | 20166501 | A | T |  |  |  |  | case | 89.8(0.0) | 100 | WDR35 |
| 2 | 20166625 | T | C |  |  |  |  | case | 67.8(0.0) | NA | WDR35 |
| 2 | 20169239 | A | AC |  | Yes | Yes |  | control | NA | NA | WDR35 |
| 2 | 20169307 | G | A |  |  |  |  | control | NA | NA | WDR35 |
| 2 | 20169322 | C | T |  |  |  |  | control | NA | NA | WDR35 |
| 2 | 20173334 | A | G |  |  |  |  | case | 160.6(0.0) | NA | WDR35 |
| 2 | 20173411 | A | G |  |  |  |  | control | NA | NA | WDR35 |
| 2 | 20174266 | G | A |  |  |  |  | control | 105.0(0.0) | NA | WDR35 |
| 2 | 20174294 | T | C |  |  |  |  | case | NA | 100 | WDR35 |
| 2 | 20174394 | A | T |  |  |  |  | case | 79.0(0.0) | 100 | WDR35 |
| 2 | 20178529 | G | A |  | Yes |  |  | control | NA | NA | WDR35 |
| 2 | 20178590 | C | T |  |  |  |  | control | NA | NA | WDR35 |
| 2 | 20178613 | C | T |  |  |  |  | case | NA | NA | WDR35 |
| 2 | 20180522 | G | C |  | Yes |  |  | control | NA | NA | WDR35 |
| 2 | 20180541 | T | G |  |  |  |  | control | NA | NA | WDR35 |
| 2 | 20182253 | A | G |  | Yes |  |  | case | NA | 100 | WDR35 |
| 2 | 20188993 | C | T |  |  |  |  | control | NA | NA | WDR35 |
| 2 | 20189009 | G | A |  |  |  |  | control | NA | NA | WDR35 |
| 2 | 20189010 | T | C |  |  |  |  | control | NA | NA | WDR35 |
| 2 | 85766427 | G | A |  |  |  |  | case | NA | 100 | MAT2A |
| 2 | 85766437 | G | C |  |  |  |  | control | NA | NA | MAT2A |
| 2 | 85768211 | G | A |  |  |  |  | control | NA | NA | MAT2A |
| 2 | 85768383 | A | G |  |  |  |  | case | 111.4(0.0) | 100 | MAT2A |
| 2 | 85768799 | G | A |  |  |  |  | control | NA | NA | MAT2A |
| 2 | 85768830 | T | C |  |  |  |  | control | NA | NA | MAT2A |
| 2 | 85769098 | G | A |  |  |  |  | control | NA | NA | MAT2A |
| 2 | 85769357 | G | A |  |  |  |  | case | 89.8(0.0) | 100 | MAT2A |
| 2 | 85769834 | C | T |  | Yes |  |  | control | NA | NA | MAT2A |
| 2 | 85770056 | G | A |  |  |  |  | both | 102.4(0.0) | NA | MAT2A |
| 2 | 85770107 | G | A |  |  |  |  | case | 98.0(0.0) | NA | MAT2A |
| 2 | 85777181 | T | TGG |  | Yes | Yes |  | case | 73.0(0.0) | 100 | GGCX |
| 2 | 85777183 | C | G |  |  |  |  | both | NA | 100 | GGCX |
| 2 | 85777676 | A | AC |  | Yes | Yes |  | case | 94.8(0.0) | 100 | GGCX |
| 2 | 85777691 | C | CAT |  | Yes | Yes |  | both | 99.8(0.0) | 33.3 | GGCX |
| 2 | 85777788 | C | G |  |  |  |  | both | 96.0(0.0) | 100 | GGCX |
| 2 | 85778653 | A | G |  |  |  |  | control | NA | NA | GGCX |
| 2 | 85778666 | C | T |  |  |  |  | control | NA | NA | GGCX |
| 2 | 85778691 | G | GTGT |  |  |  |  | case | NA | NA | GGCX |
| 2 | 85779107 | C | T |  |  |  |  | case | 130.6(0.0) | NA | GGCX |
| 2 | 85779621 | A | G |  |  |  |  | control | NA | NA | GGCX |
| 2 | 85779628 | A | G |  |  |  |  | case | 94.2(0.0) | NA | GGCX |
| 2 | 85780087 | T | C |  | Yes |  |  | case | NA | NA | GGCX |
| 2 | 85780185 | T | G |  | Yes |  |  | control | NA | NA | GGCX |
| 2 | 85780189 | C | T |  | Yes |  |  | case | NA | NA | GGCX |
| 2 | 85780450 | A | G |  | Yes | Yes |  | control | NA | NA | GGCX |
| 2 | 85780464 | G | C |  |  |  |  | case | 190.0(0.0) | 100 | GGCX |
| 2 | 85780491 | T | C |  |  |  |  | control | NA | NA | GGCX |
| 2 | 85780516 | T | G |  |  |  |  | both | NA | 100 | GGCX |
| 2 | 85780545 | C | T |  |  |  |  | case | 116.6(0.0) | NA | GGCX |
| 2 | 85780594 | C | T |  |  |  |  | both | 168.4(0.0) | 100 | GGCX |
| 2 | 85780607 | A | G |  |  |  |  | both | 110.8(0.0) | 100 | GGCX |
| 2 | 85781310 | C | A |  | Yes |  |  | both | 186.2(0.0) | NA | GGCX |
| 2 | 85781395 | T | C |  |  |  |  | control | NA | NA | GGCX |
| 2 | 85785589 | C | T |  |  |  |  | control | NA | NA | GGCX |
| 2 | 85785603 | G | A |  |  |  |  | control | NA | NA | GGCX |
| 2 | 85785675 | C | G |  |  |  |  | case | NA | 100 | GGCX |
| 2 | 85786044 | G | A |  |  |  |  | case | NA | NA | GGCX |
| 2 | 85786057 | C | T |  | Yes |  |  | case | 83.6(0.0) | NA | GGCX |
| 2 | 85786091 | A | G |  |  |  |  | both | 103.0(0.0) | 100 | GGCX |
| 2 | 85786145 | A | G |  |  |  |  | case | 76.2(0.0) | 100 | GGCX |
| 2 | 85786174 | A | G |  |  |  |  | case | 140.4(0.0) | NA | GGCX |
| 2 | 85787986 | G | T |  |  |  |  | both | 122.6(42.4) | 100 | GGCX |
| 2 | 85788071 | T | C |  |  |  |  | control | NA | NA | GGCX |
| 2 | 85788510 | C | T |  |  |  |  | control | NA | NA | GGCX |
| 2 | 85788546 | G | C |  |  |  |  | control | NA | NA | GGCX |
| 2 | 85806169 | A | G |  |  |  |  | case | 127.0(0.0) | NA | VAMP8 |
| 2 | 85806245 | G | A |  |  |  |  | both | 148.0(0.0) | 100 | VAMP8 |
| 2 | 85806262 | A | G |  |  |  |  | case | NA | NA | VAMP8 |
| 2 | 85806289 | T | C |  |  |  |  | both | 116.1(42.5) | 16.7 | VAMP8 |
| 2 | 85808724 | T | A |  |  |  |  | case | NA | NA | VAMP8 |
| 2 | 85808815 | T | C |  |  |  |  | case | NA | NA | VAMP8 |
| 2 | 85818846 | G | A |  | Yes | Yes |  | control | NA | NA | VAMP5 |
| 2 | 85820099 | C | A |  |  |  |  | control | NA | NA | VAMP5 |
| 2 | 85820137 | T | C |  |  |  |  | both | 156.9(0.0) | 100 | VAMP5 |
| 2 | 85820139 | A | G |  |  |  |  | control | NA | NA | VAMP5 |
| 2 | 85820187 | A | T |  |  |  |  | control | NA | NA | VAMP5 |
| 2 | 85820224 | G | A |  |  |  |  | case | 104.0(0.0) | NA | VAMP5 |
| 2 | 85820243 | T | C |  |  |  |  | case | 91.6(0.0) | 100 | VAMP5 |
| 2 | 85820259 | C | T |  |  |  |  | control | NA | NA | VAMP5 |
| 2 | 203644319 | A | G |  |  |  |  | case | 89.8(0.0) | 100 | ICA1L |
| 2 | 203644359 | A | C |  |  |  |  | control | NA | NA | ICA1L |
| 2 | 203653562 | T | C |  |  |  |  | both | 100.2(55.6) | 42.9 | ICA1L |
| 2 | 203653569 | A | C |  |  |  |  | control | 94.2(14.7) | 50 | ICA1L |
| 2 | 203653622 | T | C | Yes |  |  |  | both | 99.4(43.1) | 41.7 | ICA1L |
| 2 | 203653802 | A | C |  |  |  |  | case | NA | NA | ICA1L |
| 2 | 203661645 | G | A |  |  |  |  | case | 137.2(0.0) | NA | ICA1L |
| 2 | 203679471 | C | T |  |  |  |  | control | NA | NA | ICA1L |
| 2 | 203679483 | G | T |  |  |  |  | control | NA | NA | ICA1L |
| 2 | 203680706 | C | T |  |  |  |  | control | NA | NA | ICA1L |
| 2 | 203682151 | C | G |  |  |  |  | case | 122.2(0.0) | 100 | ICA1L |
| 2 | 203684462 | A | C |  | Yes |  |  | both | 115.6(39.5) | 40 | ICA1L |
| 2 | 203684617 | A | G | Yes |  |  |  | both | 118.2(0.0) | 33.3 | ICA1L |
| 2 | 203686100 | G | A |  |  |  |  | control | NA | NA | ICA1L |
| 2 | 203693601 | T | C |  |  |  |  | control | NA | NA | ICA1L |
| 2 | 203693629 | T | C |  | Yes |  |  | case | 108.4(0.0) | 100 | ICA1L |
| 2 | 203745639 | G | C |  | Yes |  |  | case | 158.8(0.0) | NA | WDR12 |
| 2 | 203745658 | C | T |  |  |  |  | control | NA | NA | WDR12 |
| 2 | 203747433 | T | C |  | Yes | Yes |  | case | NA | 100 | WDR12 |
| 2 | 203747463 | T | C |  |  |  |  | case | 95.2(0.0) | NA | WDR12 |
| 2 | 203747493 | C | G |  |  |  |  | both | 110.0(10.5) | 33.3 | WDR12 |
| 2 | 203748354 | T | C |  |  |  |  | control | NA | NA | WDR12 |
| 2 | 203748460 | C | A |  |  |  |  | case | 135.0(0.0) | NA | WDR12 |
| 2 | 203748950 | C | T |  |  |  |  | both | 81.0(0.0) | 50 | WDR12 |
| 2 | 203748969 | G | A |  | Yes |  |  | control | NA | NA | WDR12 |
| 2 | 203748970 | C | T |  |  |  |  | case | 90.8(0.0) | 100 | WDR12 |
| 2 | 203748972 | T | C |  | Yes |  |  | case | 60.2(0.0) | 100 | WDR12 |
| 2 | 203748990 | A | G |  |  |  |  | case | NA | 100 | WDR12 |
| 2 | 203749158 | T | C |  |  |  |  | control | NA | NA | WDR12 |
| 2 | 203749162 | G | T |  |  |  |  | case | 114.0(0.0) | 100 | WDR12 |
| 2 | 203749186 | G | A |  |  |  |  | control | NA | NA | WDR12 |
| 2 | 203749199 | G | A |  |  |  |  | both | 76.8(0.0) | 100 | WDR12 |
| 2 | 203749217 | T | G |  |  |  |  | case | 113.0(0.0) | NA | WDR12 |
| 2 | 203760814 | C | T |  |  |  |  | case | 157.8(0.0) | NA | WDR12 |
| 2 | 203760891 | A | AAG |  | Yes | Yes |  | control | NA | NA | WDR12 |
| 2 | 203760913 | C | A |  |  |  |  | case | NA | 100 | WDR12 |
| 2 | 203760933 | A | G |  |  |  |  | control | NA | NA | WDR12 |
| 2 | 203762037 | A | G |  |  |  |  | control | NA | NA | WDR12 |
| 2 | 203762120 | G | A |  |  |  |  | case | 94.8(0.0) | 100 | WDR12 |
| 2 | 203764257 | C | T |  |  |  |  | case | NA | 100 | WDR12 |
| 2 | 203764267 | A | C |  |  |  |  | case | NA | 100 | WDR12 |
| 2 | 203764321 | T | C |  |  |  |  | case | NA | 100 | WDR12 |
| 2 | 203772631 | A | G |  |  |  |  | case | NA | 100 | WDR12 |
| 2 | 203772679 | G | A |  |  |  |  | case | 180.0(0.0) | NA | WDR12 |
| 2 | 203776202 | A | C |  |  |  |  | control | NA | NA | WDR12 |
| 2 | 203806656 | G | A |  |  |  |  | both | 128.8(4.8) | 100 | CARF |
| 2 | 203806667 | T | C |  |  |  |  | control | NA | NA | CARF |
| 2 | 203806680 | G | A |  |  |  |  | case | NA | NA | CARF |
| 2 | 203806686 | G | A |  |  |  |  | both | 127.6(0.0) | NA | CARF |
| 2 | 203807473 | A | G |  |  |  |  | case | NA | 100 | CARF |
| 2 | 203807511 | C | T |  |  |  |  | case | 147.6(0.0) | NA | CARF |
| 2 | 203807517 | G | A |  |  |  |  | case | 94.0(0.0) | 100 | CARF |
| 2 | 203807601 | C | CT |  | Yes | Yes |  | control | NA | NA | CARF |
| 2 | 203807612 | A | G |  |  |  |  | case | NA | NA | CARF |
| 2 | 203817288 | C | G |  |  |  |  | case | 83.8(0.0) | NA | CARF |
| 2 | 203817298 | T | C |  |  |  |  | case | NA | NA | CARF |
| 2 | 203817305 | G | A |  |  |  |  | control | NA | NA | CARF |
| 2 | 203817366 | A | C |  | Yes |  |  | control | NA | NA | CARF |
| 2 | 203818769 | C | G |  |  |  |  | both | 113.8(0.0) | 33.3 | CARF |
| 2 | 203818810 | A | T |  |  |  |  | control | NA | NA | CARF |
| 2 | 203818818 | T | C |  |  |  |  | control | NA | NA | CARF |
| 2 | 203820476 | C | T |  | Yes |  |  | case | 141.5(0.0) | NA | CARF |
| 2 | 203825963 | T | A |  | Yes |  |  | control | NA | NA | CARF |
| 2 | 203826059 | T | C |  | Yes |  |  | case | 80.4(0.0) | NA | CARF |
| 2 | 203831794 | G | A |  |  |  |  | control | NA | NA | CARF |
| 2 | 203836246 | C | T |  |  |  |  | case | 124.9(0.0) | NA | CARF |
| 2 | 203836408 | G | A |  |  |  |  | case | NA | 100 | CARF |
| 2 | 203839108 | C | T |  |  |  |  | case | 135.0(0.0) | 100 | CARF |
| 2 | 203846286 | T | C |  |  |  |  | control | NA | NA | CARF |
| 2 | 203846313 | A | G |  |  |  |  | control | NA | NA | CARF |
| 2 | 203846950 | G | C |  |  |  |  | control | NA | NA | CARF |
| 2 | 203846955 | G | A |  |  |  |  | control | NA | NA | CARF |
| 2 | 203847017 | T | C |  |  |  |  | case | 106.6(0.0) | 100 | CARF |
| 2 | 203847032 | C | G |  |  |  |  | both | 149.2(0.0) | 100 | CARF |
| 2 | 203847163 | G | T |  |  |  |  | case | NA | 100 | CARF |
| 2 | 203848278 | G | A |  |  |  |  | both | 103.4(0.0) | 100 | CARF |
| 2 | 203848300 | G | A |  |  |  |  | case | 85.8(0.0) | 100 | CARF |
| 2 | 203848325 | G | A |  |  |  |  | control | NA | NA | CARF |
| 2 | 203881148 | G | A |  |  |  |  | case | 95.2(0.0) | NA | NBEAL1 |
| 2 | 203906553 | T | C |  |  |  |  | both | 99.0(22.3) | 27.3 | NBEAL1 |
| 2 | 203906577 | G | C |  |  |  |  | control | NA | NA | NBEAL1 |
| 2 | 203914553 | T | C |  |  |  |  | control | NA | NA | NBEAL1 |
| 2 | 203914652 | T | C |  | Yes | Yes |  | both | 127.8(77.0) | 33.3 | NBEAL1 |
| 2 | 203914694 | C | T |  |  |  |  | case | 149.9(23.4) | 16.7 | NBEAL1 |
| 2 | 203922114 | T | C |  | Yes |  |  | case | NA | NA | NBEAL1 |
| 2 | 203933117 | T | C |  | Yes | Yes |  | control | NA | NA | NBEAL1 |
| 2 | 203933183 | A | G |  |  |  |  | control | NA | NA | NBEAL1 |
| 2 | 203942542 | T | C |  |  |  |  | case | 118.2(0.0) | NA | NBEAL1 |
| 2 | 203942552 | A | G |  |  |  |  | control | NA | NA | NBEAL1 |
| 2 | 203947955 | G | A |  |  |  |  | case | 77.0(0.0) | 50 | NBEAL1 |
| 2 | 203947991 | A | G |  |  |  |  | case | 56.7(0.0) | 50 | NBEAL1 |
| 2 | 203948044 | G | A |  |  |  |  | both | 122.2(0.0) | NA | NBEAL1 |
| 2 | 203948050 | A | G |  |  |  |  | control | 94.2(0.0) | 100 | NBEAL1 |
| 2 | 203948110 | T | G |  |  |  |  | case | NA | 100 | NBEAL1 |
| 2 | 203948147 | G | A |  |  |  |  | both | 122.2(34.5) | 26.5 | NBEAL1 |
| 2 | 203948218 | T | C |  | Yes | Yes |  | case | 89.8(0.0) | 50 | NBEAL1 |
| 2 | 203948224 | G | A |  |  |  |  | case | 147.0(0.0) | NA | NBEAL1 |
| 2 | 203948245 | T | C |  |  |  |  | case | 94.0(0.0) | 100 | NBEAL1 |
| 2 | 203949204 | G | T |  | Yes | Yes |  | both | 142.2(25.5) | 33.3 | NBEAL1 |
| 2 | 203964355 | C | T | Yes |  |  |  | both | 114.2(31.3) | 29.2 | NBEAL1 |
| 2 | 203964359 | G | A |  |  |  |  | case | 101.6(0.0) | 100 | NBEAL1 |
| 2 | 203972384 | G | A |  |  |  |  | control | 128.8(25.7) | NA | NBEAL1 |
| 2 | 203972385 | G | C |  |  |  |  | both | 73.1(29.8) | 40 | NBEAL1 |
| 2 | 203972564 | G | A |  |  |  |  | case | 83.0(0.0) | 100 | NBEAL1 |
| 2 | 203972637 | T | C |  | Yes | Yes |  | case | 140.6(0.0) | NA | NBEAL1 |
| 2 | 203972696 | T | C |  |  |  |  | control | 89.0(0.0) | 100 | NBEAL1 |
| 2 | 203972703 | T | C |  | Yes | Yes |  | both | 109.2(14.8) | 44.4 | NBEAL1 |
| 2 | 203972761 | G | A | Yes |  |  |  | control | 71.8(0.0) | 100 | NBEAL1 |
| 2 | 203972872 | T | G |  |  |  |  | case | 151.0(0.0) | NA | NBEAL1 |
| 2 | 203974930 | T | G |  |  |  |  | case | 78.4(0.0) | NA | NBEAL1 |
| 2 | 203976818 | G | T |  |  |  |  | control | NA | NA | NBEAL1 |
| 2 | 203976819 | T | G |  | Yes | Yes |  | control | NA | NA | NBEAL1 |
| 2 | 203977902 | G | A |  |  |  |  | control | NA | NA | NBEAL1 |
| 2 | 203977918 | G | A |  |  |  |  | control | 78.6(0.0) | 100 | NBEAL1 |
| 2 | 203977930 | G | A |  |  |  |  | case | 113.2(0.0) | NA | NBEAL1 |
| 2 | 203977932 | A | C |  |  |  |  | both | 53.0(0.0) | 33.3 | NBEAL1 |
| 2 | 203977966 | C | G |  |  |  |  | case | 153.8(7.9) | 50 | NBEAL1 |
| 2 | 203977980 | T | A |  |  |  |  | control | NA | NA | NBEAL1 |
| 2 | 203977991 | C | T |  |  |  |  | both | 113.1(9.4) | 66.7 | NBEAL1 |
| 2 | 203978008 | T | C |  | Yes | Yes |  | control | 37.0(0.0) | 50 | NBEAL1 |
| 2 | 203987011 | T | C |  |  |  |  | case | NA | 100 | NBEAL1 |
| 2 | 203987035 | G | C |  |  |  |  | case | 80.6(0.0) | 100 | NBEAL1 |
| 2 | 203987059 | T | A |  |  |  |  | case | 123.0(41.0) | 33.3 | NBEAL1 |
| 2 | 203990094 | A | G | Yes | Yes |  |  | both | 119.4(44.1) | 38.5 | NBEAL1 |
| 2 | 203990157 | T | C |  |  |  |  | case | 94.6(0.0) | 33.3 | NBEAL1 |
| 2 | 203990246 | G | A |  | Yes |  |  | control | NA | NA | NBEAL1 |
| 2 | 203991287 | A | G |  |  |  |  | both | 121.2(35.9) | 42.1 | NBEAL1 |
| 2 | 203991361 | A | C |  |  |  |  | both | 120.9(37.5) | 38.7 | NBEAL1 |
| 2 | 203991404 | C | G |  |  |  |  | both | 135.0(21.8) | 100 | NBEAL1 |
| 2 | 203992564 | T | G |  |  |  |  | control | NA | NA | NBEAL1 |
| 2 | 203995082 | T | C |  |  |  |  | control | NA | NA | NBEAL1 |
| 2 | 203995111 | A | T |  |  |  |  | control | NA | NA | NBEAL1 |
| 2 | 203995127 | C | A |  |  |  |  | case | 121.0(0.0) | NA | NBEAL1 |
| 2 | 203995148 | C | A |  |  |  |  | both | 102.6(16.9) | 57.1 | NBEAL1 |
| 2 | 203996726 | A | G | Yes |  |  |  | control | 59.0(0.0) | 100 | NBEAL1 |
| 2 | 203996741 | C | A | Yes |  |  |  | both | 115.8(38.1) | 36.3 | NBEAL1 |
| 2 | 203996753 | A | C |  |  |  |  | control | NA | NA | NBEAL1 |
| 2 | 203996792 | G | C |  |  |  |  | both | 95.1(13.4) | 30 | NBEAL1 |
| 2 | 203997831 | G | A |  | Yes |  |  | control | NA | NA | NBEAL1 |
| 2 | 204000477 | C | T |  |  |  |  | control | NA | NA | NBEAL1 |
| 2 | 204000547 | G | A |  |  |  |  | control | NA | NA | NBEAL1 |
| 2 | 204000584 | G | A | Yes |  |  |  | case | 106.8(15.6) | 50 | NBEAL1 |
| 2 | 204000645 | A | G |  |  |  |  | control | NA | NA | NBEAL1 |
| 2 | 204000670 | T | G |  |  |  |  | case | 130.9(43.7) | 55.6 | NBEAL1 |
| 2 | 204000849 | T | C |  |  |  |  | control | NA | NA | NBEAL1 |
| 2 | 204000893 | A | T | Yes |  |  |  | both | 104.6(35.2) | 36.4 | NBEAL1 |
| 2 | 204002960 | T | C |  | Yes |  |  | control | NA | NA | NBEAL1 |
| 2 | 204002989 | G | A |  |  |  |  | control | NA | NA | NBEAL1 |
| 2 | 204003033 | T | G |  | Yes | Yes |  | control | NA | NA | NBEAL1 |
| 2 | 204003381 | C | A |  |  |  |  | both | 114.2(33.8) | 30.9 | NBEAL1 |
| 2 | 204003465 | G | T |  |  |  |  | case | 84.8(30.8) | 50 | NBEAL1 |
| 2 | 204009332 | G | A | Yes |  |  |  | both | 145.0(0.0) | NA | NBEAL1 |
| 2 | 204009432 | C | A |  |  |  |  | control | NA | NA | NBEAL1 |
| 2 | 204009475 | T | C |  |  |  |  | case | 73.3(32.4) | 100 | NBEAL1 |
| 2 | 204009790 | T | C |  |  |  |  | case | NA | 50 | NBEAL1 |
| 2 | 204009856 | AGG | A |  | Yes | Yes |  | case | 130.2(0.0) | NA | NBEAL1 |
| 2 | 204009881 | T | C |  | Yes | Yes |  | control | 96.4(17.4) | 75 | NBEAL1 |
| 2 | 204013719 | A | G |  |  |  |  | control | NA | NA | NBEAL1 |
| 2 | 204013807 | G | A |  |  |  |  | case | 129.8(9.9) | NA | NBEAL1 |
| 2 | 204013814 | A | G |  |  |  |  | control | NA | NA | NBEAL1 |
| 2 | 204013844 | A | C |  |  |  |  | case | NA | NA | NBEAL1 |
| 2 | 204013852 | AG | A |  | Yes | Yes |  | control | NA | NA | NBEAL1 |
| 2 | 204016221 | C | T |  |  |  |  | both | 118.4(31.7) | 35.3 | NBEAL1 |
| 2 | 204016251 | A | G |  |  |  |  | control | NA | NA | NBEAL1 |
| 2 | 204016277 | T | C |  |  |  |  | case | 190.0(0.0) | 100 | NBEAL1 |
| 2 | 204022445 | G | A |  |  |  |  | case | 103.4(0.0) | 100 | NBEAL1 |
| 2 | 204022450 | C | T |  |  |  |  | control | NA | NA | NBEAL1 |
| 2 | 204022498 | A | G |  |  |  |  | both | 126.8(25.1) | 35.7 | NBEAL1 |
| 2 | 204022523 | C | G |  |  |  |  | control | 99.9(41.4) | 33.3 | NBEAL1 |
| 2 | 204030932 | T | C |  |  |  |  | control | NA | NA | NBEAL1 |
| 2 | 204030933 | G | A |  |  |  |  | both | 136.3(24.7) | 50 | NBEAL1 |
| 2 | 204030936 | G | A |  |  |  |  | control | NA | NA | NBEAL1 |
| 2 | 204030946 | G | A |  |  |  |  | both | 125.6(33.7) | 28.6 | NBEAL1 |
| 2 | 204030975 | G | A |  |  |  |  | case | 136.6(0.0) | NA | NBEAL1 |
| 2 | 204032004 | A | G |  | Yes |  |  | both | 101.8(0.0) | 100 | NBEAL1 |
| 2 | 204032011 | T | C |  |  |  |  | both | 107.8(29.3) | 37.5 | NBEAL1 |
| 2 | 204032018 | T | A |  | Yes | Yes |  | case | NA | NA | NBEAL1 |
| 2 | 204032045 | A | G |  |  |  |  | case | 122.5(26.7) | 25 | NBEAL1 |
| 2 | 204034492 | A | G | Yes |  |  |  | case | 100.3(0.0) | NA | NBEAL1 |
| 2 | 204034502 | G | A |  |  |  |  | both | 110.7(28.2) | 42.4 | NBEAL1 |
| 2 | 204034523 | C | T |  |  |  |  | both | 105.2(0.0) | NA | NBEAL1 |
| 2 | 204034542 | T | C |  | Yes | Yes |  | both | 90.1(26.4) | 50 | NBEAL1 |
| 2 | 204037480 | T | C |  | Yes |  |  | case | 146.1(4.1) | 33.3 | NBEAL1 |
| 2 | 204037527 | C | A |  | Yes |  |  | control | 94.0(0.0) | NA | NBEAL1 |
| 2 | 204040003 | G | A |  |  |  |  | case | 115.6(0.0) | 100 | NBEAL1 |
| 2 | 204045161 | C | T |  |  |  |  | control | NA | NA | NBEAL1 |
| 2 | 204045229 | A | C |  |  |  |  | case | 123.6(0.0) | 100 | NBEAL1 |
| 2 | 204048052 | G | A |  |  |  |  | control | NA | NA | NBEAL1 |
| 2 | 204048059 | T | C |  | Yes |  |  | both | 118.2(6.2) | 12.5 | NBEAL1 |
| 2 | 204053289 | A | T | Yes | Yes |  |  | control | 113.8(0.0) | 100 | NBEAL1 |
| 2 | 204058588 | C | T |  |  |  |  | case | NA | 50 | NBEAL1 |
| 2 | 204058614 | G | C |  |  |  |  | case | 115.7(30.4) | 37.5 | NBEAL1 |
| 2 | 204062039 | C | T |  |  |  |  | case | 121.4(0.0) | NA | NBEAL1 |
| 2 | 204064099 | G | A |  |  |  |  | case | NA | 100 | NBEAL1 |
| 2 | 204066402 | G | A |  |  |  |  | both | 129.3(39.1) | NA | NBEAL1 |
| 2 | 204066416 | G | C | Yes |  |  |  | both | 124.9(40.9) | 35 | NBEAL1 |
| 2 | 204067474 | C | T |  |  |  |  | case | 100.2(0.0) | NA | NBEAL1 |
| 2 | 204073472 | T | C |  |  |  |  | both | 105.1(3.4) | 66.7 | NBEAL1 |
| 2 | 204073473 | G | A |  |  |  |  | control | 88.4(0.0) | NA | NBEAL1 |
| 2 | 204073891 | T | C | Yes |  |  |  | control | 104.8(0.0) | NA | NBEAL1 |
| 2 | 204073939 | A | G | Yes |  |  |  | case | NA | 33.3 | NBEAL1 |
| 2 | 204073953 | C | A |  |  |  |  | case | 111.7(10.3) | 100 | NBEAL1 |
| 2 | 204078272 | T | C |  | Yes | Yes |  | case | 95.0(0.0) | 100 | NBEAL1 |
| 2 | 204078278 | T | C | Yes |  |  |  | both | 113.3(12.2) | 10 | NBEAL1 |
| 2 | 204081984 | G | A |  |  |  |  | case | 50.2(0.0) | 100 | NBEAL1 |
| 2 | 204110618 | G | A |  |  |  |  | case | 162.8(71.0) | 66.7 | CYP20A1 |
| 2 | 204111624 | G | A |  |  |  |  | case | 120.0(0.0) | NA | CYP20A1 |
| 2 | 204116760 | A | C | Yes |  |  |  | control | 99.3(2.4) | NA | CYP20A1 |
| 2 | 204131261 | C | T |  | Yes |  |  | case | 131.9(13.5) | 33.3 | CYP20A1 |
| 2 | 204131287 | T | C |  |  |  |  | control | 130.1(16.0) | 18.2 | CYP20A1 |
| 2 | 204131309 | T | C |  |  |  |  | control | NA | NA | CYP20A1 |
| 2 | 204131313 | A | G |  |  |  |  | control | NA | NA | CYP20A1 |
| 2 | 204131355 | T | C | Yes |  |  |  | control | NA | 100 | CYP20A1 |
| 2 | 204131371 | C | G | Yes |  |  |  | control | 106.6(38.6) | 26.3 | CYP20A1 |
| 2 | 204131388 | G | A |  |  |  |  | case | 135.2(74.7) | 27.3 | CYP20A1 |
| 2 | 204131404 | G | A |  |  |  |  | both | 85.4(0.0) | NA | CYP20A1 |
| 2 | 204154508 | A | G | Yes |  |  |  | control | NA | NA | CYP20A1 |
| 2 | 204154550 | G | A |  |  |  |  | control | NA | NA | CYP20A1 |
| 2 | 204154580 | A | G |  |  |  |  | control | NA | NA | CYP20A1 |
| 2 | 204161284 | A | G |  |  |  |  | control | 97.5(18.2) | 57.1 | CYP20A1 |
| 2 | 204161503 | A | G | Yes |  |  |  | both | 113.1(41.8) | 36.8 | CYP20A1 |
| 2 | 204161513 | T | G |  |  |  |  | both | 129.5(40.2) | 23.1 | CYP20A1 |
| 2 | 204161517 | G | A |  |  |  |  | control | NA | NA | CYP20A1 |
| 4 | 148406876 | A | G |  |  |  |  | case | NA | NA | EDNRA |
| 4 | 148406920 | C | T |  |  |  |  | control | NA | NA | EDNRA |
| 4 | 148407060 | T | C |  |  |  |  | both | 138.8(22.6) | 66.7 | EDNRA |
| 4 | 148407091 | T | A |  |  |  |  | both | 57.2(0.0) | 100 | EDNRA |
| 4 | 148407139 | G | C |  |  |  |  | case | 92.2(0.0) | NA | EDNRA |
| 4 | 148441072 | C | T |  |  |  |  | case | 48.8(0.0) | 100 | EDNRA |
| 4 | 148453782 | A | G |  |  |  |  | control | NA | NA | EDNRA |
| 4 | 148453801 | G | A |  | Yes |  |  | control | NA | NA | EDNRA |
| 4 | 148457082 | T | C |  |  |  |  | control | NA | NA | EDNRA |
| 4 | 148461071 | A | G |  |  |  |  | control | NA | NA | EDNRA |
| 4 | 148461086 | A | C |  |  |  |  | case | 82.8(0.0) | 100 | EDNRA |
| 4 | 148461608 | T | G |  |  |  |  | control | NA | NA | EDNRA |
| 4 | 148463677 | T | G |  |  |  |  | control | NA | NA | EDNRA |
| 4 | 156618095 | A | C |  |  |  |  | case | 90.0(0.0) | NA | GUCY1A3 |
| 4 | 156618180 | G | A |  |  |  |  | case | 76.2(0.0) | 100 | GUCY1A3 |
| 4 | 156618196 | C | T |  |  |  |  | control | NA | NA | GUCY1A3 |
| 4 | 156618216 | A | G |  |  |  |  | control | NA | NA | GUCY1A3 |
| 4 | 156618258 | G | A |  |  |  |  | control | NA | NA | GUCY1A3 |
| 4 | 156625118 | C | T |  |  |  |  | control | NA | NA | GUCY1A3 |
| 4 | 156629398 | C | G |  |  |  |  | case | NA | NA | GUCY1A3 |
| 4 | 156631705 | T | G |  | Yes | Yes |  | case | 100.8(0.0) | NA | GUCY1A3 |
| 4 | 156631711 | G | A |  |  |  |  | control | NA | NA | GUCY1A3 |
| 4 | 156631752 | T | C |  |  |  |  | case | NA | NA | GUCY1A3 |
| 4 | 156631753 | A | G |  |  |  |  | both | 125.2(0.0) | 50 | GUCY1A3 |
| 4 | 156631808 | T | A |  | Yes |  |  | both | 65.2(0.0) | NA | GUCY1A3 |
| 4 | 156631876 | T | G |  |  |  |  | case | 90.6(0.0) | 100 | GUCY1A3 |
| 4 | 156631883 | G | C |  | Yes |  |  | case | 128.8(0.0) | NA | GUCY1A3 |
| 4 | 156631890 | G | A |  |  |  |  | both | 111.6(0.0) | 100 | GUCY1A3 |
| 4 | 156632009 | C | T |  |  |  |  | case | 212.2(0.0) | 100 | GUCY1A3 |
| 4 | 156632073 | T | C |  |  |  |  | control | NA | NA | GUCY1A3 |
| 4 | 156632093 | C | T |  |  |  |  | control | NA | NA | GUCY1A3 |
| 4 | 156632127 | G | A |  |  |  |  | case | NA | 100 | GUCY1A3 |
| 4 | 156632163 | T | C |  |  |  |  | case | 82.0(0.0) | 100 | GUCY1A3 |
| 4 | 156632188 | C | A |  |  |  |  | case | 86.8(0.0) | 100 | GUCY1A3 |
| 4 | 156634336 | G | A |  |  |  |  | case | 111.6(0.0) | 100 | GUCY1A3 |
| 4 | 156634352 | A | G |  | Yes |  |  | control | NA | NA | GUCY1A3 |
| 4 | 156634354 | T | G |  |  |  |  | case | 45.4(36.3) | 20 | GUCY1A3 |
| 4 | 156634624 | T | C |  |  |  |  | case | 136.6(0.0) | NA | GUCY1A3 |
| 4 | 156638348 | T | G |  | Yes |  |  | case | 93.8(0.0) | 100 | GUCY1A3 |
| 4 | 156638364 | G | T |  |  |  |  | control | NA | NA | GUCY1A3 |
| 4 | 156643231 | T | C |  |  |  |  | control | NA | NA | GUCY1A3 |
| 4 | 156643232 | A | G |  |  |  |  | case | 91.0(0.0) | 100 | GUCY1A3 |
| 4 | 156643248 | T | C |  | Yes |  |  | control | NA | NA | GUCY1A3 |
| 4 | 156643282 | A | G |  |  |  |  | control | NA | NA | GUCY1A3 |
| 4 | 156651241 | G | C |  |  |  |  | both | NA | 100 | GUCY1A3 |
| 4 | 156651242 | G | A |  |  |  |  | both | 110.4(34.5) | 43.4 | GUCY1A3 |
| 4 | 156651264 | A | G |  | Yes |  |  | control | NA | NA | GUCY1A3 |
| 4 | 156651270 | G | T |  |  |  |  | case | 123.7(0.0) | 100 | GUCY1A3 |
| 4 | 156651325 | G | A |  |  |  |  | control | NA | NA | GUCY1A3 |
| 4 | 156651326 | C | G |  |  |  |  | case | 159.4(0.0) | NA | GUCY1A3 |
| 4 | 156651343 | A | G |  |  |  |  | control | NA | NA | GUCY1A3 |
| 4 | 156651360 | A | G |  |  |  |  | case | 86.7(0.0) | NA | GUCY1A3 |
| 4 | 156680941 | T | C |  |  |  |  | control | NA | NA | GUCY1B3 |
| 4 | 156696144 | G | A |  |  |  |  | control | NA | NA | GUCY1B3 |
| 4 | 156696157 | A | G |  |  |  |  | control | NA | NA | GUCY1B3 |
| 4 | 156698704 | A | G |  |  |  |  | control | NA | NA | GUCY1B3 |
| 4 | 156698752 | C | A |  |  |  |  | control | NA | NA | GUCY1B3 |
| 4 | 156698760 | A | G |  | Yes |  |  | case | 91.8(0.0) | NA | GUCY1B3 |
| 4 | 156710901 | A | C |  |  |  |  | both | 59.0(0.0) | 100 | GUCY1B3 |
| 4 | 156710978 | T | C |  | Yes |  |  | case | 131.0(0.0) | 50 | GUCY1B3 |
| 4 | 156711064 | T | G |  | Yes | Yes |  | case | 145.0(0.0) | 50 | GUCY1B3 |
| 4 | 156715042 | T | C | Yes |  |  |  | control | 119.0(0.0) | NA | GUCY1B3 |
| 4 | 156715053 | C | A |  |  |  |  | case | 129.9(0.0) | 100 | GUCY1B3 |
| 4 | 156715112 | T | A | Yes |  |  |  | case | 108.6(25.9) | 63.2 | GUCY1B3 |
| 4 | 156715136 | T | C |  |  |  |  | control | 109.8(32.3) | 28.6 | GUCY1B3 |
| 4 | 156715218 | G | A |  |  |  |  | control | NA | NA | GUCY1B3 |
| 4 | 156716599 | C | T | Yes | Yes |  |  | case | 101.0(28.3) | 66.7 | GUCY1B3 |
| 4 | 156717588 | G | C |  |  |  |  | case | 96.6(13.9) | NA | GUCY1B3 |
| 4 | 156717599 | A | G |  |  |  |  | case | NA | NA | GUCY1B3 |
| 4 | 156721028 | C | G |  | Yes | Yes |  | case | NA | NA | GUCY1B3 |
| 4 | 156721054 | C | A |  |  |  |  | both | 98.3(32.4) | 71.4 | GUCY1B3 |
| 4 | 156721152 | G | A |  |  |  |  | both | 113.0(35.7) | 35.4 | GUCY1B3 |
| 4 | 156723517 | T | C |  |  |  |  | both | NA | 100 | GUCY1B3 |
| 4 | 156723518 | A | G |  |  |  |  | both | 92.7(43.1) | 33.3 | GUCY1B3 |
| 4 | 156723545 | A | G |  |  |  |  | case | 88.8(0.0) | 100 | GUCY1B3 |
| 4 | 156724781 | A | G |  |  |  |  | case | 82.1(37.7) | 33.3 | GUCY1B3 |
| 4 | 156724909 | G | C |  |  |  |  | control | 170.6(0.0) | 100 | GUCY1B3 |
| 4 | 156726298 | T | G |  | Yes | Yes |  | case | NA | NA | GUCY1B3 |
| 4 | 156726353 | G | T |  | Yes |  |  | control | NA | NA | GUCY1B3 |
| 6 | 134210577 | G | GGT |  | Yes | Yes |  | control | NA | NA | TCF21 |
| 6 | 134210602 | A | T |  |  |  |  | case | 106.8(0.0) | NA | TCF21 |
| 6 | 134210609 | C | T |  |  |  |  | both | 97.8(0.0) | 100 | TCF21 |
| 6 | 134210640 | T | C |  | Yes |  |  | control | NA | NA | TCF21 |
| 6 | 134210665 | A | T |  |  |  |  | both | 125.0(0.0) | 100 | TCF21 |
| 6 | 134210680 | T | C |  |  |  |  | both | NA | 100 | TCF21 |
| 6 | 134210727 | A | C |  | Yes |  |  | control | NA | NA | TCF21 |
| 6 | 134210752 | C | A |  | Yes |  |  | case | NA | 100 | TCF21 |
| 6 | 134210779 | C | A |  | Yes |  |  | both | 112.3(29.7) | 35.7 | TCF21 |
| 6 | 134210924 | G | A |  | Yes |  |  | case | NA | NA | TCF21 |
| 6 | 134210983 | T | C |  | Yes |  |  | control | NA | NA | TCF21 |
| 6 | 161127492 | G | A | Yes |  |  |  | both | 109.9(23.7) | 23.1 | PLG |
| 6 | 161127495 | A | G |  |  |  |  | case | 152.0(0.0) | 50 | PLG |
| 6 | 161127549 | A | G | Yes |  |  |  | both | 112.9(29.6) | 35.2 | PLG |
| 6 | 161128749 | C | G |  |  |  |  | case | NA | NA | PLG |
| 6 | 161128779 | C | A |  |  |  |  | control | NA | NA | PLG |
| 6 | 161132139 | T | G | Yes |  |  |  | control | 102.4(7.6) | 33.3 | PLG |
| 6 | 161134094 | A | C |  |  |  |  | control | NA | NA | PLG |
| 6 | 161134138 | T | C |  |  |  |  | case | 125.3(18.0) | 66.7 | PLG |
| 6 | 161134159 | C | T |  | Yes | Yes |  | case | 95.0(0.0) | 100 | PLG |
| 6 | 161137765 | T | C | Yes |  |  |  | control | 102.4(0.0) | NA | PLG |
| 6 | 161139389 | A | G |  |  |  |  | control | NA | NA | PLG |
| 6 | 161139390 | T | C |  |  |  |  | both | 119.5(27.8) | 42.9 | PLG |
| 6 | 161139414 | T | C |  |  |  |  | case | 95.7(0.0) | 100 | PLG |
| 6 | 161139732 | C | G | Yes |  |  |  | case | 99.7(37.9) | 20 | PLG |
| 6 | 161139800 | A | G |  |  |  |  | control | NA | NA | PLG |
| 6 | 161139802 | A | G | Yes |  |  |  | control | 88.2(0.0) | 50 | PLG |
| 6 | 161143500 | A | G | Yes |  |  |  | both | 120.1(34.2) | 37.1 | PLG |
| 6 | 161143509 | T | C |  |  |  |  | control | NA | NA | PLG |
| 6 | 161143519 | T | C |  |  |  |  | both | 100.7(30.6) | 16.7 | PLG |
| 6 | 161143553 | G | A |  |  |  |  | case | 97.6(0.0) | 100 | PLG |
| 6 | 161152089 | A | G |  |  |  |  | control | NA | NA | PLG |
| 6 | 161152156 | A | G | Yes |  |  |  | both | 122.0(35.9) | 37.5 | PLG |
| 6 | 161152197 | G | A |  |  |  |  | both | 156.4(0.0) | NA | PLG |
| 6 | 161152229 | T | TCC |  | Yes | Yes |  | control | NA | NA | PLG |
| 6 | 161152233 | G | GC |  | Yes | Yes |  | control | NA | NA | PLG |
| 6 | 161152260 | G | A |  |  |  |  | both | 99.4(20.3) | 23.8 | PLG |
| 6 | 161152897 | G | C | Yes |  |  |  | both | 95.1(26.7) | 14.3 | PLG |
| 6 | 161155073 | T | C |  |  |  |  | case | 100.0(0.0) | NA | PLG |
| 6 | 161157920 | A | G |  |  |  |  | both | 80.1(17.4) | 25 | PLG |
| 6 | 161157945 | T | C |  |  |  |  | case | 98.0(0.0) | NA | PLG |
| 6 | 161157986 | C | G |  |  |  |  | control | NA | NA | PLG |
| 6 | 161158004 | G | A |  |  |  |  | case | 87.2(0.0) | 100 | PLG |
| 6 | 161159626 | T | C |  |  |  |  | control | NA | NA | PLG |
| 6 | 161159627 | C | T |  |  |  |  | case | 140.8(0.0) | 33.3 | PLG |
| 6 | 161159645 | T | G |  | Yes | Yes |  | control | NA | NA | PLG |
| 6 | 161159647 | T | A |  |  |  |  | case | 143.4(0.0) | NA | PLG |
| 6 | 161162392 | G | A | Yes |  |  |  | control | 84.4(0.0) | 66.7 | PLG |
| 6 | 161162404 | A | G | Yes |  |  |  | case | 84.0(0.0) | NA | PLG |
| 6 | 161162415 | T | C |  |  |  |  | both | 102.5(17.9) | 30.8 | PLG |
| 6 | 161162442 | C | A |  |  |  |  | case | NA | NA | PLG |
| 6 | 161173148 | C | T |  |  |  |  | case | 141.0(0.0) | NA | PLG |
| 6 | 161173172 | A | G |  |  |  |  | both | 105.3(30.7) | 37.9 | PLG |
| 6 | 161173222 | C | T |  |  |  |  | case | 91.0(0.0) | NA | PLG |
| 6 | 161174074 | A | G |  |  |  |  | both | 114.4(40.1) | 31 | PLG |
| 6 | 161174080 | A | T |  |  |  |  | control | 88.8(29.5) | NA | PLG |
| 7 | 18201950 | T | G |  | Yes | Yes |  | control | NA | NA | HDAC9 |
| 7 | 18498490 | T | G |  |  |  |  | both | 122.2(36.8) | 50 | HDAC9 |
| 7 | 18535891 | A | T |  |  |  |  | control | NA | NA | HDAC9 |
| 7 | 18624954 | A | G |  |  |  |  | case | NA | 100 | HDAC9 |
| 7 | 18625031 | T | A |  | Yes |  |  | control | NA | NA | HDAC9 |
| 7 | 18625046 | T | G |  |  |  |  | case | 136.9(0.0) | 100 | HDAC9 |
| 7 | 18629994 | G | A |  |  |  |  | case | NA | NA | HDAC9 |
| 7 | 18630047 | C | T |  |  |  |  | case | 71.8(0.0) | 100 | HDAC9 |
| 7 | 18630109 | G | A |  | Yes |  |  | control | NA | NA | HDAC9 |
| 7 | 18631170 | A | G |  |  |  |  | control | NA | NA | HDAC9 |
| 7 | 18631202 | T | C |  |  |  |  | control | NA | NA | HDAC9 |
| 7 | 18669065 | A | G |  |  |  |  | control | NA | NA | HDAC9 |
| 7 | 18687466 | C | T |  |  |  |  | both | 105.2(45.4) | 80 | HDAC9 |
| 7 | 18687468 | G | C |  |  |  |  | case | 111.4(0.0) | 100 | HDAC9 |
| 7 | 18688097 | T | C |  |  |  |  | control | NA | NA | HDAC9 |
| 7 | 18688234 | T | G |  |  |  |  | control | NA | NA | HDAC9 |
| 7 | 18688300 | T | G |  |  |  |  | case | NA | 100 | HDAC9 |
| 7 | 18705844 | A | G |  |  |  |  | case | 81.7(12.9) | 50 | HDAC9 |
| 7 | 18705911 | C | G |  |  |  |  | control | NA | NA | HDAC9 |
| 7 | 18705971 | G | A |  |  |  |  | case | 96.6(0.0) | NA | HDAC9 |
| 7 | 18706095 | G | A |  |  |  |  | control | NA | NA | HDAC9 |
| 7 | 18706097 | A | C |  |  |  |  | case | 131.8(0.0) | NA | HDAC9 |
| 7 | 18706133 | G | A |  |  |  |  | case | 136.4(0.0) | NA | HDAC9 |
| 7 | 18767323 | A | G |  |  |  |  | case | 88.0(0.0) | 100 | HDAC9 |
| 7 | 18767345 | T | C |  |  |  |  | both | 103.5(39.3) | 66.7 | HDAC9 |
| 7 | 18788749 | C | A |  |  |  |  | control | NA | NA | HDAC9 |
| 7 | 18801869 | T | C |  |  |  |  | control | NA | NA | HDAC9 |
| 7 | 18833001 | A | T |  |  |  |  | case | 82.2(0.0) | 100 | HDAC9 |
| 7 | 18833048 | C | T |  |  |  |  | case | 119.0(0.0) | 100 | HDAC9 |
| 7 | 18868810 | C | T |  |  |  |  | case | 129.9(0.0) | 100 | HDAC9 |
| 7 | 18875534 | A | C |  |  |  |  | case | 112.4(36.8) | 60 | HDAC9 |
| 7 | 18875535 | G | C |  |  |  |  | both | 129.6(41.6) | 40 | HDAC9 |
| 7 | 18875603 | A | G |  |  |  |  | control | NA | NA | HDAC9 |
| 7 | 18914105 | G | A |  |  |  |  | both | 112.5(8.3) | 66.7 | HDAC9 |
| 7 | 18914204 | G | A |  |  |  |  | case | 94.0(0.0) | 100 | HDAC9 |
| 7 | 18914207 | G | GT |  | Yes | Yes |  | control | NA | NA | HDAC9 |
| 7 | 18914218 | T | A |  |  |  |  | control | NA | NA | HDAC9 |
| 7 | 18975448 | A | G |  |  |  |  | case | 166.1(0.0) | NA | HDAC9 |
| 7 | 18975567 | T | TA |  |  |  |  | case | 118.5(16.5) | 100 | HDAC9 |
| 7 | 19015496 | T | G |  |  |  |  | both | 108.8(35.1) | 33.3 | HDAC9 |
| 7 | 19015514 | T | C |  |  |  |  | control | NA | NA | HDAC9 |
| 7 | 19015517 | C | T |  |  |  |  | case | 81.5(0.0) | NA | HDAC9 |
| 7 | 19015524 | T | C |  |  |  |  | both | 125.3(21.3) | 75 | HDAC9 |
| 7 | 19015555 | A | C |  |  |  |  | both | 11.6(0.0) | NA | HDAC9 |
| 7 | 19015564 | G | A |  |  |  |  | control | NA | NA | HDAC9 |
| 9 | 21968234 | A | G |  |  |  |  | both | 109.5(41.3) | 55.6 | CDKN2A |
| 9 | 21968238 | T | A |  |  |  |  | case | 114.0(32.3) | 53.8 | CDKN2A |
| 9 | 21968753 | G | A |  |  |  |  | both | 111.7(20.7) | 38.5 | CDKN2A |
| 9 | 21970953 | T | C |  |  |  |  | both | 114.1(28.2) | 31 | CDKN2A |
| 9 | 21970961 | G | C |  |  |  |  | case | 166.8(0.0) | 100 | CDKN2A |
| 9 | 21971007 | T | C |  |  |  |  | case | 0.0(0.0) | 100 | CDKN2A |
| 9 | 21971043 | T | G | Yes |  |  |  | both | 116.0(33.6) | 30.8 | CDKN2A |
| 9 | 21971122 | A | G | Yes |  |  |  | case | 97.9(10.3) | 33.3 | CDKN2A |
| 9 | 21994139 | C | T |  |  |  |  | control | NA | NA | CDKN2A |
| 9 | 21994156 | T | G |  |  |  |  | case | NA | 100 | CDKN2A |
| 9 | 21994182 | G | A |  |  |  |  | case | 155.3(0.0) | 100 | CDKN2A |
| 9 | 21994253 | A | G |  |  |  |  | both | 103.8(21.3) | 37.5 | CDKN2A |
| 9 | 21994284 | A | C |  |  |  |  | control | NA | NA | CDKN2A |
| 9 | 21994787 | G | C |  |  |  |  | case | 106.9(0.0) | 100 | CDKN2BAS1 |
| 9 | 21994791 | C | G |  |  |  |  | both | 113.2(0.0) | 20 | CDKN2BAS1 |
| 9 | 21994809 | T | C |  |  |  |  | case | 101.9(24.4) | 53.3 | CDKN2BAS1 |
| 9 | 21994814 | T | C |  |  |  |  | control | NA | NA | CDKN2BAS1 |
| 9 | 21994835 | C | T |  |  |  |  | both | 115.2(31.0) | 40 | CDKN2BAS1 |
| 9 | 21994918 | G | A |  |  |  |  | case | 96.4(6.5) | 66.7 | CDKN2BAS1 |
| 9 | 21994949 | T | A |  |  |  |  | case | 108.0(12.7) | 33.3 | CDKN2BAS1 |
| 9 | 21995112 | A | G |  |  |  |  | both | 109.3(30.4) | 22.7 | CDKN2BAS1 |
| 9 | 22005990 | C | T |  |  |  |  | both | 131.0(20.2) | 100 | CDKN2B |
| 9 | 22006008 | G | A |  |  |  |  | control | NA | NA | CDKN2B |
| 9 | 22006223 | T | G |  | Yes |  |  | case | 121.4(0.0) | NA | CDKN2B |
| 9 | 22008716 | T | C |  |  |  |  | case | 100.4(0.0) | NA | CDKN2B |
| 9 | 22008739 | A | G |  |  |  |  | case | 149.2(8.6) | 100 | CDKN2B |
| 9 | 22008786 | T | G |  |  |  |  | control | NA | NA | CDKN2B |
| 9 | 22008806 | C | G |  |  |  |  | case | 101.2(0.0) | NA | CDKN2B |
| 9 | 22008809 | T | C |  |  |  |  | control | NA | NA | CDKN2B |
| 9 | 22008824 | A | G |  |  |  |  | case | 128.6(0.0) | NA | CDKN2B |
| 9 | 22008882 | G | C |  |  |  |  | case | NA | NA | CDKN2B |
| 9 | 22029436 | G | A |  |  |  |  | case | NA | NA | CDKN2BAS1 |
| 9 | 22029506 | T | C |  |  |  |  | case | 134.6(0.0) | NA | CDKN2BAS1 |
| 9 | 22029508 | G | C |  |  |  |  | case | 124.3(0.0) | NA | CDKN2BAS1 |
| 9 | 22029513 | G | C |  |  |  |  | case | 105.3(12.3) | 50 | CDKN2BAS1 |
| 9 | 22029552 | A | G |  |  |  |  | control | NA | NA | CDKN2BAS1 |
| 9 | 22029584 | G | A |  |  |  |  | both | 106.4(35.7) | 55.6 | CDKN2BAS1 |
| 9 | 22032712 | A | G |  |  |  |  | case | NA | 100 | CDKN2BAS1 |
| 9 | 22032774 | T | C |  |  |  |  | both | 110.7(38.6) | 37 | CDKN2BAS1 |
| 9 | 22032813 | A | C |  |  |  |  | case | 90.6(0.0) | 100 | CDKN2BAS1 |
| 9 | 22032832 | G | C |  |  |  |  | both | 112.5(23.6) | 15.4 | CDKN2BAS1 |
| 9 | 22032892 | T | C |  |  |  |  | both | 145.0(0.0) | NA | CDKN2BAS1 |
| 9 | 22032902 | G | A |  |  |  |  | both | NA | NA | CDKN2BAS1 |
| 9 | 22032914 | G | C |  |  |  |  | control | NA | NA | CDKN2BAS1 |
| 9 | 22032976 | C | T |  |  |  |  | control | NA | NA | CDKN2BAS1 |
| 9 | 22046384 | A | C |  |  |  |  | case | 130.2(0.0) | 100 | CDKN2BAS1 |
| 9 | 22046393 | A | G |  |  |  |  | both | 108.0(0.0) | 25 | CDKN2BAS1 |
| 9 | 22046421 | T | C |  |  |  |  | case | 118.7(23.6) | 25 | CDKN2BAS1 |
| 9 | 22046443 | A | G |  |  |  |  | both | 87.3(18.6) | 43.8 | CDKN2BAS1 |
| 9 | 22046832 | T | G |  |  |  |  | both | 99.0(0.0) | 100 | CDKN2BAS1 |
| 9 | 22046884 | A | T |  |  |  |  | case | 120.3(33.2) | NA | CDKN2BAS1 |
| 9 | 22046892 | C | A |  |  |  |  | case | 120.3(33.2) | NA | CDKN2BAS1 |
| 9 | 22049144 | A | C |  |  |  |  | case | NA | NA | CDKN2BAS1 |
| 9 | 22049158 | C | T |  |  |  |  | control | NA | NA | CDKN2BAS1 |
| 9 | 22049173 | T | C |  |  |  |  | case | 61.0(0.0) | 100 | CDKN2BAS1 |
| 9 | 22049213 | T | C |  |  |  |  | case | 64.4(0.0) | 100 | CDKN2BAS1 |
| 9 | 22056314 | C | G |  |  |  |  | control | NA | NA | CDKN2BAS1 |
| 9 | 22056334 | G | T |  |  |  |  | control | NA | NA | CDKN2BAS1 |
| 9 | 22056358 | T | C |  |  |  |  | both | 90.4(0.0) | 100 | CDKN2BAS1 |
| 9 | 22056363 | A | G |  |  |  |  | case | 178.2(0.0) | 100 | CDKN2BAS1 |
| 9 | 22058528 | T | C |  |  |  |  | control | 147.8(0.0) | NA | CDKN2BAS1 |
| 9 | 22058578 | A | C |  |  |  |  | control | NA | NA | CDKN2BAS1 |
| 9 | 22058604 | C | CT |  |  |  |  | case | 109.0(0.0) | NA | CDKN2BAS1 |
| 9 | 22058648 | G | A |  |  |  |  | control | NA | NA | CDKN2BAS1 |
| 9 | 22058803 | G | A |  |  |  |  | control | NA | NA | CDKN2BAS1 |
| 9 | 22058814 | G | A |  |  |  |  | case | NA | NA | CDKN2BAS1 |
| 9 | 22058834 | A | G |  |  |  |  | control | 87.8(0.0) | 100 | CDKN2BAS1 |
| 9 | 22058910 | A | G |  |  |  |  | case | 133.8(0.0) | 100 | CDKN2BAS1 |
| 9 | 22059014 | G | C |  |  |  |  | both | 101.5(45.9) | 50 | CDKN2BAS1 |
| 9 | 22061983 | G | T |  |  |  |  | case | 142.2(0.0) | 50 | CDKN2BAS1 |
| 9 | 22062015 | A | C |  |  |  |  | both | 127.7(23.8) | 33.3 | CDKN2BAS1 |
| 9 | 22063940 | C | T |  |  |  |  | case | 125.7(19.4) | 50 | CDKN2BAS1 |
| 9 | 22065721 | G | C |  |  |  |  | control | 189.4(0.0) | NA | CDKN2BAS1 |
| 9 | 22065753 | G | C |  |  |  |  | case | NA | NA | CDKN2BAS1 |
| 9 | 22066349 | A | C |  |  |  |  | case | 117.9(0.0) | NA | CDKN2BAS1 |
| 9 | 22077716 | A | G |  |  |  |  | control | NA | NA | CDKN2BAS1 |
| 9 | 22077728 | C | T |  |  |  |  | case | 44.6(0.0) | 100 | CDKN2BAS1 |
| 9 | 22077798 | T | C |  |  |  |  | both | 115.8(26.4) | 35.7 | CDKN2BAS1 |
| 9 | 22077892 | G | A |  |  |  |  | case | 91.0(19.2) | 100 | CDKN2BAS1 |
| 9 | 22092313 | A | G |  |  |  |  | both | 106.4(35.7) | 55.6 | CDKN2BAS1 |
| 9 | 22092334 | C | CA |  |  |  |  | case | 134.8(0.0) | NA | CDKN2BAS1 |
| 9 | 22092336 | C | T |  |  |  |  | case | 134.8(0.0) | NA | CDKN2BAS1 |
| 9 | 22092369 | G | A |  |  |  |  | control | NA | NA | CDKN2BAS1 |
| 9 | 22092452 | T | TG |  |  |  |  | case | 90.5(44.5) | 33.3 | CDKN2BAS1 |
| 9 | 22092457 | A | T |  |  |  |  | both | 110.6(23.0) | 10 | CDKN2BAS1 |
| 9 | 22096456 | G | A |  |  |  |  | both | NA | 50 | CDKN2BAS1 |
| 9 | 22096480 | G | A |  |  |  |  | both | 119.9(45.5) | 36.4 | CDKN2BAS1 |
| 9 | 22096485 | T | C |  |  |  |  | control | 130.4(34.5) | NA | CDKN2BAS1 |
| 9 | 22096510 | G | A |  |  |  |  | control | NA | NA | CDKN2BAS1 |
| 9 | 22096514 | C | G |  | Yes | Yes |  | control | 272.6(0.0) | 50 | CDKN2BAS1 |
| 9 | 22097260 | A | G |  |  |  |  | case | 81.4(0.0) | 100 | CDKN2BAS1 |
| 9 | 22097341 | G | A |  |  |  |  | case | 128.9(48.6) | 33.3 | CDKN2BAS1 |
| 9 | 22097359 | T | C |  |  |  |  | case | NA | NA | CDKN2BAS1 |
| 9 | 22097365 | T | TG |  |  |  |  | case | NA | NA | CDKN2BAS1 |
| 9 | 22112359 | A | C |  |  |  |  | case | NA | 50 | CDKN2BAS1 |
| 9 | 22113679 | T | C |  |  |  |  | both | 107.8(20.6) | 36.4 | CDKN2BAS1 |
| 9 | 22113773 | G | T |  |  |  |  | control | NA | NA | CDKN2BAS1 |
| 9 | 22118670 | G | A |  |  |  |  | both | 102.1(18.1) | 45.5 | CDKN2BAS1 |
| 9 | 22118696 | G | A |  |  |  |  | case | NA | NA | CDKN2BAS1 |
| 9 | 22120224 | C | T |  |  |  |  | both | 109.1(24.7) | 50 | CDKN2BAS1 |
| 9 | 22120289 | T | C |  |  |  |  | case | NA | 100 | CDKN2BAS1 |
| 9 | 22120535 | C | T |  |  |  |  | both | 142.1(59.4) | 27.3 | CDKN2BAS1 |
| 9 | 22120685 | G | A |  |  |  |  | case | 124.4(4.2) | NA | CDKN2BAS1 |
| 9 | 22120800 | TCA | T |  |  |  |  | both | NA | NA | CDKN2BAS1 |
| 9 | 22120804 | T | C |  |  |  |  | control | NA | NA | CDKN2BAS1 |
| 9 | 22120837 | A | G |  |  |  |  | both | 110.2(32.6) | 22.2 | CDKN2BAS1 |
| 9 | 22121012 | T | C |  |  |  |  | control | NA | NA | CDKN2BAS1 |
| 10 | 44871410 | A | C |  |  |  |  | case | NA | NA | CXCL12 |
| 10 | 44871455 | T | TC |  | Yes | Yes |  | control | NA | NA | CXCL12 |
| 10 | 44871459 | T | C |  |  |  |  | both | NA | 50 | CXCL12 |
| 10 | 44871461 | C | CT |  | Yes | Yes |  | both | NA | 100 | CXCL12 |
| 10 | 44871475 | T | C |  |  |  |  | both | 107.7(0.0) | 100 | CXCL12 |
| 10 | 44873226 | G | A |  |  |  |  | control | NA | NA | CXCL12 |
| 10 | 44873244 | T | G |  |  |  |  | case | 193.4(0.0) | 100 | CXCL12 |
| 10 | 44873256 | G | A |  |  |  |  | control | NA | NA | CXCL12 |
| 10 | 44873288 | A | G |  |  |  |  | control | NA | NA | CXCL12 |
| 10 | 44873291 | C | G |  |  |  |  | case | 59.2(0.0) | 100 | CXCL12 |
| 10 | 44873331 | T | C |  |  |  |  | control | NA | NA | CXCL12 |
| 10 | 44873332 | A | G |  |  |  |  | case | 112.2(0.0) | 100 | CXCL12 |
| 10 | 44873352 | C | G |  |  |  |  | control | NA | NA | CXCL12 |
| 10 | 44874079 | A | G |  |  |  |  | control | NA | NA | CXCL12 |
| 10 | 44874103 | G | A |  | Yes |  |  | case | 62.4(0.0) | NA | CXCL12 |
| 10 | 44874130 | A | G |  | Yes |  |  | both | 114.9(2.7) | 33.3 | CXCL12 |
| 10 | 44874138 | T | G |  | Yes | Yes |  | case | NA | NA | CXCL12 |
| 10 | 44874154 | C | T |  |  |  |  | control | NA | NA | CXCL12 |
| 10 | 44876211 | T | A |  |  |  |  | case | 166.1(0.0) | NA | CXCL12 |
| 10 | 44876278 | A | G |  |  |  |  | control | NA | NA | CXCL12 |
| 10 | 44876321 | A | G |  |  |  |  | case | NA | NA | CXCL12 |
| 11 | 103780426 | T | C | Yes | Yes |  |  | case | 67.2(0.0) | NA | PDGFD |
| 11 | 103780502 | C | T |  |  |  |  | control | NA | NA | PDGFD |
| 11 | 103780526 | A | G |  |  |  |  | case | 176.0(0.0) | 100 | PDGFD |
| 11 | 103780538 | G | A |  |  |  |  | case | 115.2(0.0) | 100 | PDGFD |
| 11 | 103780545 | A | T |  |  |  |  | case | 104.2(0.0) | NA | PDGFD |
| 11 | 103797778 | C | T |  |  |  |  | case | 100.8(0.0) | NA | PDGFD |
| 11 | 103797807 | A | G | Yes | Yes |  |  | both | 102.5(27.5) | 42.1 | PDGFD |
| 11 | 103797816 | G | T |  | Yes |  |  | control | NA | NA | PDGFD |
| 11 | 103797836 | C | T |  |  |  |  | control | NA | NA | PDGFD |
| 11 | 103797852 | A | C |  |  |  |  | control | NA | NA | PDGFD |
| 11 | 103814203 | A | G | Yes |  |  |  | both | 100.2(25.9) | 45.5 | PDGFD |
| 11 | 103814204 | G | A | Yes |  |  |  | both | 100.2(25.9) | 45.5 | PDGFD |
| 11 | 103814218 | T | C | Yes |  |  |  | both | 100.0(24.5) | 42.1 | PDGFD |
| 11 | 103814282 | A | G |  |  |  |  | control | NA | NA | PDGFD |
| 11 | 103814340 | G | A |  |  |  |  | control | NA | NA | PDGFD |
| 11 | 103814350 | A | G | Yes |  |  |  | control | 71.0(0.0) | 50 | PDGFD |
| 11 | 103814374 | C | A | Yes |  |  |  | case | 108.7(25.5) | 43.8 | PDGFD |
| 11 | 103818399 | A | G |  |  |  |  | control | NA | NA | PDGFD |
| 11 | 103866871 | T | C |  |  |  |  | both | NA | NA | PDGFD |
| 11 | 103866878 | A | G |  | Yes |  |  | both | 102.8(0.0) | 50 | PDGFD |
| 11 | 103866886 | T | C |  |  |  |  | both | 102.8(0.0) | 50 | PDGFD |
| 11 | 103866942 | T | C | Yes |  |  |  | control | NA | NA | PDGFD |
| 11 | 103866943 | A | G |  |  |  |  | case | 91.4(0.0) | 100 | PDGFD |
| 11 | 103866972 | G | A |  | Yes |  |  | both | 122.2(21.8) | 50 | PDGFD |
| 11 | 103870840 | A | G | Yes |  |  |  | control | 100.3(18.5) | NA | PDGFD |
| 11 | 103870922 | A | G |  |  |  |  | both | 116.3(30.0) | 42.9 | PDGFD |
| 11 | 104034567 | G | A | Yes |  |  |  | control | 166.9(31.8) | 33.3 | PDGFD |
| 11 | 104034601 | C | G |  |  |  |  | control | NA | NA | PDGFD |
| 11 | 104034652 | C | G |  |  |  |  | control | NA | NA | PDGFD |
| 12 | 89916595 | A | T |  | Yes | Yes |  | both | 89.4(21.2) | 62.5 | GALNT4 |
| 12 | 89916673 | A | G |  |  |  |  | case | 161.6(0.0) | NA | GALNT4 |
| 12 | 89916683 | G | A |  |  |  |  | both | 143.8(7.1) | NA | GALNT4 |
| 12 | 89916745 | C | T |  |  |  |  | control | NA | NA | GALNT4 |
| 12 | 89916750 | A | G |  |  |  |  | control | NA | NA | GALNT4 |
| 12 | 89916767 | C | T |  |  |  |  | control | NA | NA | GALNT4 |
| 12 | 89916809 | C | T |  |  |  |  | both | 148.2(0.0) | 100 | GALNT4 |
| 12 | 89916902 | G | A |  |  |  |  | control | NA | NA | GALNT4 |
| 12 | 89917024 | T | A |  |  |  |  | both | 126.4(0.0) | 100 | GALNT4 |
| 12 | 89917028 | A | AG |  | Yes | Yes |  | case | NA | 100 | GALNT4 |
| 12 | 89917075 | A | G |  |  |  |  | case | NA | 100 | GALNT4 |
| 12 | 89917148 | A | G |  |  |  |  | both | 115.6(0.0) | 100 | GALNT4 |
| 12 | 89917173 | C | A |  |  |  |  | control | NA | NA | GALNT4 |
| 12 | 89917185 | T | C |  |  |  |  | both | 106.4(34.2) | 42.9 | GALNT4 |
| 12 | 89917227 | T | C |  |  |  |  | control | NA | NA | GALNT4 |
| 12 | 89917473 | C | T |  |  |  |  | both | NA | NA | GALNT4 |
| 12 | 89917508 | A | G |  |  |  |  | case | NA | NA | GALNT4 |
| 12 | 89917538 | G | A |  |  |  |  | case | 130.5(10.6) | 50 | GALNT4 |
| 12 | 89917701 | T | C |  |  |  |  | control | NA | NA | GALNT4 |
| 12 | 89917739 | C | T |  |  |  |  | both | 69.4(0.0) | 100 | GALNT4 |
| 12 | 89917788 | A | ACT |  | Yes | Yes |  | case | 118.8(0.0) | 100 | GALNT4 |
| 12 | 89917857 | C | CTG |  | Yes | Yes |  | case | 79.0(0.0) | 100 | GALNT4 |
| 12 | 89917860 | A | T |  |  |  |  | case | 79.0(0.0) | 100 | GALNT4 |
| 12 | 89917861 | A | G |  |  |  |  | case | 79.0(0.0) | 100 | GALNT4 |
| 12 | 89918017 | A | T |  |  |  |  | case | 81.0(0.0) | 100 | GALNT4 |
| 12 | 89918037 | T | A |  |  |  |  | case | 116.0(0.0) | 100 | GALNT4 |
| 12 | 89918195 | A | C |  |  |  |  | control | NA | NA | GALNT4 |
| 12 | 111051994 | T | C |  |  |  |  | control | NA | NA | TCTN1 |
| 12 | 111052001 | A | G | Yes |  |  |  | control | NA | 100 | TCTN1 |
| 12 | 111052130 | C | T |  |  |  |  | control | NA | NA | TCTN1 |
| 12 | 111057698 | C | G |  |  |  |  | case | 91.2(0.0) | NA | TCTN1 |
| 12 | 111057751 | T | C |  |  |  |  | both | 161.4(0.0) | NA | TCTN1 |
| 12 | 111057757 | A | G |  |  |  |  | control | NA | NA | TCTN1 |
| 12 | 111064168 | A | G | Yes |  |  |  | both | 126.0(33.7) | 25.6 | TCTN1 |
| 12 | 111064171 | A | G | Yes |  |  |  | case | NA | 100 | TCTN1 |
| 12 | 111064184 | A | G |  |  |  |  | control | 108.8(0.0) | 100 | TCTN1 |
| 12 | 111064205 | G | A |  |  |  |  | case | 83.0(0.0) | 100 | TCTN1 |
| 12 | 111066721 | C | G |  |  |  |  | control | NA | NA | TCTN1 |
| 12 | 111070329 | C | T |  |  |  |  | case | 72.0(0.0) | 100 | TCTN1 |
| 12 | 111072224 | T | G |  |  |  |  | case | 86.0(5.6) | 50 | TCTN1 |
| 12 | 111072488 | T | C |  |  |  |  | control | NA | NA | TCTN1 |
| 12 | 111072530 | G | T |  | Yes |  |  | control | 143.4(0.0) | 100 | TCTN1 |
| 12 | 111078209 | G | A |  |  |  |  | case | 107.2(0.0) | NA | TCTN1 |
| 12 | 111078305 | A | G |  |  |  |  | case | 130.6(0.0) | NA | TCTN1 |
| 12 | 111078892 | T | C |  |  |  |  | control | NA | NA | TCTN1 |
| 12 | 111078905 | T | G |  |  |  |  | control | NA | NA | TCTN1 |
| 12 | 111078944 | G | A |  |  |  |  | control | NA | NA | TCTN1 |
| 12 | 111079380 | G | C |  |  |  |  | case | 142.2(0.0) | 100 | TCTN1 |
| 12 | 111079423 | G | C |  |  |  |  | control | NA | NA | TCTN1 |
| 12 | 111080134 | C | T | Yes |  |  |  | both | 76.5(40.3) | 7.7 | TCTN1 |
| 12 | 111080150 | T | C |  |  |  |  | both | 107.0(28.0) | 34.4 | TCTN1 |
| 12 | 111082894 | C | G |  |  |  |  | control | NA | NA | TCTN1 |
| 12 | 111082922 | G | A |  |  |  |  | case | 92.6(10.7) | NA | TCTN1 |
| 12 | 111085677 | A | T | Yes |  |  |  | control | NA | NA | TCTN1 |
| 12 | 111085678 | A | T | Yes |  |  |  | control | NA | NA | TCTN1 |
| 12 | 111085698 | C | CGTAA |  | Yes | Yes |  | case | 98.8(0.0) | 100 | TCTN1 |
| 12 | 111856097 | T | C |  |  |  |  | case | 104.6(0.0) | 100 | SH2B3 |
| 12 | 111856112 | T | C |  |  |  |  | case | 96.9(39.2) | 50 | SH2B3 |
| 12 | 111856567 | G | C |  |  |  |  | control | NA | NA | SH2B3 |
| 12 | 111856604 | T | C |  | Yes |  |  | case | NA | 100 | SH2B3 |
| 12 | 111856658 | T | C |  | Yes |  |  | case | NA | 100 | SH2B3 |
| 12 | 111884568 | A | C |  |  |  |  | control | NA | NA | SH2B3 |
| 12 | 111884636 | G | A |  |  |  |  | both | 124.0(44.0) | 18.8 | SH2B3 |
| 12 | 111884755 | G | C |  |  |  |  | case | 68.2(0.0) | NA | SH2B3 |
| 12 | 111884942 | A | G |  |  |  |  | case | 82.6(0.0) | 100 | SH2B3 |
| 12 | 111884989 | A | C |  |  |  |  | control | 84.6(33.2) | 57.1 | SH2B3 |
| 12 | 111885164 | G | C |  |  |  |  | control | NA | NA | SH2B3 |
| 12 | 111885304 | T | C |  | Yes |  |  | both | 110.9(28.3) | 75 | SH2B3 |
| 12 | 111885473 | T | C |  |  |  |  | control | NA | NA | SH2B3 |
| 12 | 111885474 | A | G |  |  |  |  | case | NA | 100 | SH2B3 |
| 12 | 111885487 | C | G |  | Yes |  |  | case | 85.0(0.0) | NA | SH2B3 |
| 12 | 111885522 | A | G |  |  |  |  | case | 85.0(0.0) | 50 | SH2B3 |
| 12 | 111885525 | T | C |  |  |  |  | control | 96.2(0.0) | 50 | SH2B3 |
| 12 | 111885593 | A | G |  |  |  |  | both | 123.8(32.2) | 36.4 | SH2B3 |
| 12 | 111885607 | A | G |  | Yes |  |  | case | 138.0(0.0) | 100 | SH2B3 |
| 12 | 111885998 | A | G |  |  |  |  | both | 101.4(25.0) | 35 | SH2B3 |
| 12 | 111886006 | C | A |  |  |  |  | control | NA | 50 | SH2B3 |
| 12 | 111886023 | T | C |  | Yes | Yes |  | case | NA | NA | SH2B3 |
| 12 | 111886053 | G | T |  |  |  |  | control | 114.7(5.0) | NA | SH2B3 |
| 12 | 111886061 | T | C |  |  |  |  | case | 103.0(0.0) | 100 | SH2B3 |
| 12 | 111886073 | A | G |  |  |  |  | control | NA | NA | SH2B3 |
| 12 | 111886089 | T | C |  | Yes | Yes |  | case | 134.3(33.1) | 50 | SH2B3 |
| 12 | 111886098 | T | C |  |  |  |  | case | 88.4(0.0) | 100 | SH2B3 |
| 12 | 112082097 | G | A |  |  |  |  | case | 116.7(39.6) | 81.8 | BRAP |
| 12 | 112082242 | A | C |  |  |  |  | case | 237.6(0.0) | 100 | BRAP |
| 12 | 112082326 | T | C |  |  |  |  | control | 129.6(20.0) | 33.3 | BRAP |
| 12 | 112087784 | C | T |  |  |  |  | case | 241.0(0.0) | NA | BRAP |
| 12 | 112096549 | C | T |  |  |  |  | control | NA | NA | BRAP |
| 12 | 112097031 | T | C |  |  |  |  | both | 145.1(2.7) | 33.3 | BRAP |
| 12 | 112116958 | C | T |  |  |  |  | control | NA | NA | BRAP |
| 12 | 112116989 | C | T |  |  |  |  | control | NA | NA | BRAP |
| 12 | 112117134 | C | A |  |  |  |  | control | 125.8(32.2) | 50 | BRAP |
| 12 | 112119584 | A | C |  |  |  |  | case | 118.8(0.0) | 100 | BRAP |
| 12 | 112120950 | CTGG | C |  |  |  |  | control | NA | NA | BRAP |
| 12 | 112120974 | C | T |  |  |  |  | both | 106.7(22.7) | 9.1 | BRAP |
| 12 | 112120983 | G | C |  |  |  |  | both | 111.9(35.8) | 35.7 | BRAP |
| 12 | 112121001 | T | C |  |  |  |  | control | NA | NA | BRAP |
| 12 | 112123525 | A | G |  |  |  |  | control | NA | NA | BRAP |
| 13 | 28877376 | C | T |  |  |  |  | control | NA | NA | FLT1 |
| 13 | 28877397 | A | G |  |  |  |  | case | 143.0(0.0) | NA | FLT1 |
| 13 | 28877455 | G | C |  |  |  |  | control | NA | NA | FLT1 |
| 13 | 28877476 | A | G |  |  |  |  | control | NA | NA | FLT1 |
| 13 | 28877507 | T | TAAAG |  |  |  |  | case | 114.8(0.0) | NA | FLT1 |
| 13 | 28880832 | T | C |  |  |  |  | case | 131.6(0.0) | 100 | FLT1 |
| 13 | 28880898 | A | G |  |  |  |  | case | 96.2(0.0) | NA | FLT1 |
| 13 | 28883036 | C | G |  |  |  |  | case | 121.6(0.0) | 100 | FLT1 |
| 13 | 28885798 | A | G |  |  |  |  | control | NA | NA | FLT1 |
| 13 | 28886131 | C | T |  |  |  |  | case | 141.0(61.4) | 50 | FLT1 |
| 13 | 28891657 | C | G |  | Yes |  |  | control | NA | NA | FLT1 |
| 13 | 28891658 | G | A |  |  |  |  | control | NA | NA | FLT1 |
| 13 | 28891665 | G | A |  |  |  |  | case | 42.6(0.0) | NA | FLT1 |
| 13 | 28891696 | G | A |  |  |  |  | control | NA | NA | FLT1 |
| 13 | 28891699 | T | C |  | Yes |  |  | case | NA | NA | FLT1 |
| 13 | 28891707 | G | A |  |  |  |  | control | NA | NA | FLT1 |
| 13 | 28891718 | C | T |  |  |  |  | control | NA | NA | FLT1 |
| 13 | 28893607 | G | C |  | Yes |  |  | case | 84.4(0.0) | NA | FLT1 |
| 13 | 28895642 | A | G |  |  |  |  | case | 66.4(0.0) | 50 | FLT1 |
| 13 | 28896452 | C | T |  |  |  |  | control | NA | NA | FLT1 |
| 13 | 28896973 | A | G |  |  |  |  | case | 105.6(0.0) | 100 | FLT1 |
| 13 | 28897006 | G | A |  |  |  |  | case | 96.8(32.8) | 50 | FLT1 |
| 13 | 28901655 | G | A |  |  |  |  | control | NA | NA | FLT1 |
| 13 | 28903805 | G | A |  | Yes |  |  | case | 125.0(0.0) | 100 | FLT1 |
| 13 | 28908167 | G | A |  | Yes |  |  | both | 149.2(0.0) | 100 | FLT1 |
| 13 | 28913367 | C | T |  | Yes |  |  | control | NA | NA | FLT1 |
| 13 | 28913426 | C | T |  |  |  |  | case | NA | 100 | FLT1 |
| 13 | 28919629 | A | T |  |  |  |  | case | 81.0(0.0) | 100 | FLT1 |
| 13 | 28919667 | C | A |  |  |  |  | control | NA | NA | FLT1 |
| 13 | 28931696 | G | A |  | Yes |  |  | case | NA | 100 | FLT1 |
| 13 | 28931725 | A | G |  |  |  |  | case | 95.4(0.0) | NA | FLT1 |
| 13 | 28931784 | G | C |  |  |  |  | both | 109.9(22.1) | 66.7 | FLT1 |
| 13 | 28931802 | T | TTCC |  |  |  |  | control | NA | NA | FLT1 |
| 13 | 28942735 | G | A |  |  |  |  | control | NA | NA | FLT1 |
| 13 | 28942739 | C | T |  |  |  |  | control | NA | NA | FLT1 |
| 13 | 28942770 | A | G |  |  |  |  | control | NA | NA | FLT1 |
| 13 | 28959085 | C | T |  |  |  |  | both | 101.8(19.5) | 100 | FLT1 |
| 13 | 28959120 | A | G |  |  |  |  | both | 136.0(29.2) | 33.3 | FLT1 |
| 13 | 28959143 | G | T |  |  |  |  | both | 63.4(22.6) | 66.7 | FLT1 |
| 13 | 28964144 | A | G |  |  |  |  | case | 119.0(0.0) | 100 | FLT1 |
| 13 | 28964147 | C | T |  |  |  |  | case | 198.1(0.0) | 100 | FLT1 |
| 13 | 28964216 | C | G |  |  |  |  | both | NA | 100 | FLT1 |
| 13 | 28964228 | T | C |  |  |  |  | control | NA | NA | FLT1 |
| 13 | 28971094 | C | T |  |  |  |  | case | 96.6(56.3) | 50 | FLT1 |
| 13 | 28971098 | C | T |  |  |  |  | control | NA | NA | FLT1 |
| 13 | 28973184 | T | C |  |  |  |  | control | NA | NA | FLT1 |
| 13 | 28973240 | G | T |  |  |  |  | case | 117.2(0.0) | NA | FLT1 |
| 13 | 28973254 | C | A |  |  |  |  | control | NA | NA | FLT1 |
| 13 | 28979920 | C | A |  |  |  |  | control | NA | NA | FLT1 |
| 13 | 29001313 | G | A |  |  |  |  | case | NA | NA | FLT1 |
| 13 | 29001316 | A | G |  |  |  |  | both | 124.0(0.0) | NA | FLT1 |
| 13 | 29001396 | T | G |  |  |  |  | control | NA | NA | FLT1 |
| 13 | 29001410 | A | G |  |  |  |  | both | NA | 100 | FLT1 |
| 13 | 29001417 | A | G |  |  |  |  | control | NA | NA | FLT1 |
| 13 | 29001891 | G | T |  |  |  |  | control | NA | NA | FLT1 |
| 13 | 29001915 | C | T |  | Yes |  |  | case | NA | 50 | FLT1 |
| 13 | 29001933 | A | T |  |  |  |  | control | NA | NA | FLT1 |
| 13 | 29001960 | C | T |  |  |  |  | both | 111.8(45.8) | 50 | FLT1 |
| 13 | 29001992 | G | A |  |  |  |  | case | NA | NA | FLT1 |
| 13 | 29001999 | A | G |  |  |  |  | both | 154.2(0.0) | 100 | FLT1 |
| 13 | 29002021 | A | G |  |  |  |  | control | NA | NA | FLT1 |
| 13 | 29002038 | A | G |  |  |  |  | control | NA | NA | FLT1 |
| 13 | 29004224 | C | T |  |  |  |  | case | 91.2(0.0) | 100 | FLT1 |
| 13 | 29004233 | A | G |  | Yes |  |  | both | 119.9(43.6) | 66.7 | FLT1 |
| 13 | 29004288 | C | A |  |  |  |  | control | NA | NA | FLT1 |
| 13 | 29005280 | G | A |  |  |  |  | control | NA | NA | FLT1 |
| 13 | 29008022 | T | G |  |  |  |  | case | NA | 100 | FLT1 |
| 13 | 29008070 | G | T |  |  |  |  | both | 82.4(57.8) | 50 | FLT1 |
| 13 | 29008220 | T | C |  |  |  |  | control | NA | NA | FLT1 |
| 13 | 29008247 | G | T |  |  |  |  | control | NA | NA | FLT1 |
| 13 | 29008268 | A | T |  |  |  |  | both | 121.2(40.9) | 66.7 | FLT1 |
| 13 | 29041128 | C | G |  |  |  |  | case | 142.4(0.0) | 100 | FLT1 |
| 13 | 29041134 | C | T |  |  |  |  | control | NA | NA | FLT1 |
| 13 | 29041211 | G | T |  |  |  |  | case | 72.0(0.0) | 100 | FLT1 |
| 13 | 29041692 | C | T |  |  |  |  | case | 68.4(0.0) | NA | FLT1 |
| 13 | 29041742 | G | C |  |  |  |  | case | 103.4(0.0) | NA | FLT1 |
| 13 | 29068921 | A | G |  |  |  |  | case | 79.4(0.0) | NA | FLT1 |
| 15 | 91418970 | T | C |  |  |  |  | control | NA | NA | FURIN |
| 15 | 91418981 | A | G |  |  |  |  | case | 101.0(0.0) | 100 | FURIN |
| 15 | 91419028 | G | C |  |  |  |  | case | 132.6(0.0) | 100 | FURIN |
| 15 | 91419035 | G | C |  |  |  |  | case | 99.2(0.0) | 100 | FURIN |
| 15 | 91419063 | G | C |  |  |  |  | control | NA | NA | FURIN |
| 15 | 91419080 | A | G |  |  |  |  | control | NA | NA | FURIN |
| 15 | 91419107 | G | A |  |  |  |  | case | 123.8(0.0) | NA | FURIN |
| 15 | 91419119 | A | G |  |  |  |  | case | NA | NA | FURIN |
| 15 | 91419524 | G | A |  |  |  |  | control | NA | NA | FURIN |
| 15 | 91419563 | T | C |  |  |  |  | both | NA | NA | FURIN |
| 15 | 91419726 | T | G |  | Yes |  |  | case | NA | NA | FURIN |
| 15 | 91419742 | A | G |  |  |  |  | case | 125.3(44.3) | 100 | FURIN |
| 15 | 91419785 | A | C |  |  |  |  | both | 124.8(0.0) | 100 | FURIN |
| 15 | 91420204 | G | A |  |  |  |  | case | 103.0(0.0) | NA | FURIN |
| 15 | 91420371 | A | G |  |  |  |  | case | 123.7(0.0) | NA | FURIN |
| 15 | 91420417 | A | G |  |  |  |  | case | 121.0(0.0) | 100 | FURIN |
| 15 | 91420815 | A | G |  |  |  |  | case | 69.8(0.0) | 100 | FURIN |
| 15 | 91420833 | A | G |  | Yes |  |  | both | 120.0(0.0) | 50 | FURIN |
| 15 | 91420838 | T | C |  |  |  |  | case | 116.0(0.0) | 100 | FURIN |
| 15 | 91420839 | G | A |  |  |  |  | both | 138.0(0.0) | 100 | FURIN |
| 15 | 91421411 | T | G |  |  |  |  | control | NA | NA | FURIN |
| 15 | 91422071 | T | C |  |  |  |  | case | 110.6(0.0) | 100 | FURIN |
| 15 | 91422111 | A | G |  |  |  |  | control | NA | NA | FURIN |
| 15 | 91422134 | A | G |  |  |  |  | control | NA | NA | FURIN |
| 15 | 91422162 | T | C |  |  |  |  | both | 84.6(0.0) | 50 | FURIN |
| 15 | 91422681 | G | C |  |  |  |  | control | NA | NA | FURIN |
| 15 | 91422969 | T | C |  |  |  |  | case | NA | NA | FURIN |
| 15 | 91423180 | T | C |  | Yes |  |  | control | NA | NA | FURIN |
| 15 | 91423206 | T | C |  |  |  |  | case | 135.8(0.0) | NA | FURIN |
| 15 | 91423407 | T | C |  |  |  |  | control | NA | NA | FURIN |
| 15 | 91423447 | T | C |  |  |  |  | case | 75.2(0.0) | 100 | FURIN |
| 15 | 91423492 | C | G |  |  |  |  | case | 90.0(0.0) | 100 | FURIN |
| 15 | 91423936 | T | C |  |  |  |  | control | NA | NA | FURIN |
| 15 | 91424238 | A | G |  |  |  |  | control | NA | NA | FURIN |
| 15 | 91424250 | T | C |  |  |  |  | control | NA | NA | FURIN |
| 15 | 91424273 | T | C |  |  |  |  | control | NA | NA | FURIN |
| 15 | 91424577 | T | C |  |  |  |  | control | NA | NA | FURIN |
| 15 | 91424814 | T | G |  |  |  |  | both | 81.4(60.9) | 60 | FURIN |
| 15 | 91424877 | T | C |  |  |  |  | control | NA | NA | FURIN |
| 15 | 91424910 | A | C |  |  |  |  | control | NA | NA | FURIN |
| 15 | 91424911 | A | G |  |  |  |  | both | 116.5(32.3) | 37.5 | FURIN |
| 15 | 91424928 | T | C |  |  |  |  | both | 105.5(23.9) | 100 | FURIN |
| 15 | 91424994 | T | C |  |  |  |  | case | 148.2(0.0) | 100 | FURIN |
| 15 | 91428336 | T | G |  | Yes | Yes |  | case | 118.0(0.0) | 100 | FES |
| 15 | 91428363 | G | A |  |  |  |  | case | NA | 100 | FES |
| 15 | 91428410 | G | A |  |  |  |  | control | NA | NA | FES |
| 15 | 91428447 | A | G |  |  |  |  | case | 107.3(40.9) | 100 | FES |
| 15 | 91428747 | T | C |  | Yes |  |  | case | 100.9(15.8) | 50 | FES |
| 15 | 91430450 | C | T |  |  |  |  | control | NA | NA | FES |
| 15 | 91430453 | A | G |  |  |  |  | case | 85.0(0.0) | NA | FES |
| 15 | 91430490 | C | T |  |  |  |  | control | NA | NA | FES |
| 15 | 91430517 | G | A |  |  |  |  | case | NA | NA | FES |
| 15 | 91430529 | T | C |  |  |  |  | control | NA | NA | FES |
| 15 | 91430551 | A | G |  |  |  |  | both | 127.7(43.1) | 50 | FES |
| 15 | 91430554 | G | C |  | Yes |  |  | control | NA | NA | FES |
| 15 | 91430560 | T | C |  |  |  |  | both | 118.4(0.0) | 100 | FES |
| 15 | 91432592 | C | T |  |  |  |  | case | 98.2(0.0) | 100 | FES |
| 15 | 91432655 | T | G |  |  |  |  | both | 92.8(0.0) | 100 | FES |
| 15 | 91432668 | C | G |  |  |  |  | control | NA | NA | FES |
| 15 | 91432744 | T | C |  |  |  |  | control | NA | NA | FES |
| 15 | 91432751 | A | G |  |  |  |  | both | 64.8(0.0) | 100 | FES |
| 15 | 91432820 | C | G |  | Yes |  |  | both | NA | NA | FES |
| 15 | 91433089 | T | G |  | Yes | Yes |  | case | NA | NA | FES |
| 15 | 91433106 | T | C |  |  |  |  | control | NA | NA | FES |
| 15 | 91433192 | A | G |  |  |  |  | control | NA | NA | FES |
| 15 | 91433641 | A | G |  |  |  |  | control | NA | NA | FES |
| 15 | 91433645 | T | G |  |  |  |  | control | NA | NA | FES |
| 15 | 91434209 | A | C |  |  |  |  | both | 100.8(0.0) | NA | FES |
| 15 | 91434221 | T | C |  |  |  |  | control | NA | NA | FES |
| 15 | 91434238 | A | G |  |  |  |  | control | NA | NA | FES |
| 15 | 91434274 | T | C |  |  |  |  | case | 87.0(0.0) | NA | FES |
| 15 | 91434374 | A | G |  | Yes |  |  | case | 132.8(0.0) | 100 | FES |
| 15 | 91434393 | T | C |  |  |  |  | both | 109.8(37.8) | 41.2 | FES |
| 15 | 91434781 | T | C |  |  |  |  | both | 118.4(41.2) | 50 | FES |
| 15 | 91434784 | T | A |  | Yes |  |  | case | NA | NA | FES |
| 15 | 91434794 | T | G |  |  |  |  | case | NA | NA | FES |
| 15 | 91434866 | G | C |  | Yes |  |  | case | 158.0(0.0) | NA | FES |
| 15 | 91436386 | G | A |  | Yes |  |  | case | 151.6(0.0) | NA | FES |
| 15 | 91436405 | T | C |  |  |  |  | control | NA | NA | FES |
| 15 | 91436406 | A | G |  |  |  |  | case | 85.5(0.0) | 100 | FES |
| 15 | 91436524 | A | G |  | Yes |  |  | both | 130.8(0.0) | 100 | FES |
| 15 | 91436564 | T | G |  |  |  |  | both | 165.2(0.0) | 50 | FES |
| 15 | 91436593 | T | G |  |  |  |  | both | NA | NA | FES |
| 15 | 91436957 | A | G |  |  |  |  | control | NA | NA | FES |
| 15 | 91437039 | T | A |  |  |  |  | case | 76.2(0.0) | 100 | FES |
| 15 | 91437168 | T | C |  | Yes |  |  | both | 113.7(25.3) | 33.3 | FES |
| 15 | 91437178 | G | C |  | Yes |  |  | control | NA | NA | FES |
| 15 | 91438748 | C | T |  | Yes |  |  | control | NA | NA | FES |
| 15 | 91438772 | A | G |  |  |  |  | both | NA | NA | FES |
| 15 | 91438778 | A | G |  |  |  |  | case | NA | 100 | FES |
| 17 | 1964849 | T | C |  |  |  |  | control | 113.0(0.0) | NA | SMG6 |
| 17 | 1968859 | T | C |  |  |  |  | control | NA | NA | SMG6 |
| 17 | 1968867 | A | G |  |  |  |  | control | NA | NA | SMG6 |
| 17 | 1968974 | A | C |  | Yes | Yes |  | case | NA | NA | SMG6 |
| 17 | 1972120 | A | C |  |  |  |  | case | 91.6(0.0) | 100 | SMG6 |
| 17 | 1972163 | C | G |  |  |  |  | control | 89.8(0.0) | 100 | SMG6 |
| 17 | 1972190 | T | C |  |  |  |  | case | NA | NA | SMG6 |
| 17 | 1985104 | G | C |  |  |  |  | control | NA | NA | SMG6 |
| 17 | 1985229 | A | C |  |  |  |  | control | NA | NA | SMG6 |
| 17 | 1989034 | A | T |  |  |  |  | case | 140.8(0.0) | NA | SMG6 |
| 17 | 1989078 | T | C |  |  |  |  | control | NA | NA | SMG6 |
| 17 | 1989098 | G | T | Yes |  |  |  | both | 137.3(2.5) | 57.1 | SMG6 |
| 17 | 1989104 | A | C |  |  |  |  | control | NA | NA | SMG6 |
| 17 | 1989108 | C | T |  |  |  |  | case | 68.4(0.0) | NA | SMG6 |
| 17 | 1989177 | C | T |  |  |  |  | control | NA | NA | SMG6 |
| 17 | 2075972 | T | C | Yes |  |  |  | both | 107.2(38.9) | 36.1 | SMG6 |
| 17 | 2076025 | T | C | Yes |  |  |  | both | 100.8(19.5) | 33.3 | SMG6 |
| 17 | 2076090 | A | C |  |  |  |  | case | 106.4(0.0) | 100 | SMG6 |
| 17 | 2076132 | T | C |  |  |  |  | case | 128.6(0.0) | 50 | SMG6 |
| 17 | 2076141 | G | A |  |  |  |  | control | 95.1(25.0) | 28.6 | SMG6 |
| 17 | 2089972 | G | A | Yes |  |  |  | both | 152.2(90.4) | 66.7 | SMG6 |
| 17 | 2090009 | T | C |  |  |  |  | control | NA | NA | SMG6 |
| 17 | 2090087 | T | C |  |  |  |  | control | NA | NA | SMG6 |
| 17 | 2091699 | T | C |  |  |  |  | case | NA | 100 | SMG6 |
| 17 | 2139855 | C | T | Yes |  |  |  | control | 85.0(0.0) | 100 | SMG6 |
| 17 | 2147982 | C | T |  |  |  |  | both | 101.9(24.9) | 27.8 | SMG6 |
| 17 | 2186017 | A | G |  |  |  |  | control | NA | NA | SMG6 |
| 17 | 2186053 | T | G |  |  |  |  | case | NA | NA | SMG6 |
| 17 | 2186055 | A | G |  |  |  |  | case | 123.0(15.0) | 66.7 | SMG6 |
| 17 | 2186119 | G | A |  |  |  |  | case | NA | NA | SMG6 |
| 17 | 2186917 | T | A |  | Yes | Yes |  | control | NA | NA | SMG6 |
| 17 | 2186929 | A | G |  |  |  |  | control | NA | NA | SMG6 |
| 17 | 2186996 | A | G |  | Yes |  |  | control | NA | NA | SMG6 |
| 17 | 2196187 | T | C |  |  |  |  | case | 203.2(0.0) | 50 | SMG6 |
| 17 | 2201177 | T | G | Yes | Yes |  |  | case | 113.0(12.7) | 100 | SMG6 |
| 17 | 2202292 | A | G |  |  |  |  | control | NA | NA | SMG6 |
| 17 | 2202485 | C | G | Yes | Yes |  |  | both | 113.2(30.2) | 42.1 | SMG6 |
| 17 | 2202512 | T | C | Yes |  |  |  | case | 107.1(31.3) | 50 | SMG6 |
| 17 | 2202514 | C | G |  |  |  |  | both | 112.0(30.2) | 28.6 | SMG6 |
| 17 | 2202581 | G | C |  |  |  |  | control | NA | NA | SMG6 |
| 17 | 2202623 | C | T |  |  |  |  | case | NA | NA | SMG6 |
| 17 | 2202686 | T | C | Yes |  |  |  | both | 127.0(57.4) | 44.4 | SMG6 |
| 17 | 2202850 | C | T |  |  |  |  | control | 126.8(39.1) | 50 | SMG6 |
| 17 | 2202882 | G | A | Yes |  |  |  | both | 89.5(38.4) | 33.3 | SMG6 |
| 17 | 2202944 | A | G |  |  |  |  | case | 166.8(0.0) | 100 | SMG6 |
| 17 | 2203046 | T | C | Yes |  |  |  | both | 102.0(23.0) | 31.6 | SMG6 |
| 17 | 2203073 | G | C |  |  |  |  | control | NA | NA | SMG6 |
| 17 | 2203076 | C | T |  |  |  |  | case | 97.0(0.0) | 100 | SMG6 |
| 17 | 2203143 | T | C |  |  |  |  | control | NA | NA | SMG6 |
| 17 | 2203206 | A | G | Yes |  |  |  | control | 86.6(45.8) | 25 | SMG6 |
| 17 | 2203226 | A | G | Yes |  |  |  | control | 119.0(45.3) | 66.7 | SMG6 |
| 17 | 2203324 | C | T |  |  |  |  | case | NA | 100 | SMG6 |
| 17 | 2203357 | A | G |  |  |  |  | both | NA | 100 | SMG6 |
| 17 | 2203384 | C | T |  |  |  |  | control | NA | NA | SMG6 |
| 17 | 2203396 | C | T |  |  |  |  | case | 112.0(11.3) | 66.7 | SMG6 |
| 17 | 2203477 | C | T |  |  |  |  | case | 63.4(0.0) | 100 | SMG6 |
| 17 | 2203524 | A | G |  |  |  |  | case | 118.2(0.0) | 100 | SMG6 |
| 17 | 2203710 | T | C | Yes |  |  |  | both | 126.4(0.0) | NA | SMG6 |
| 17 | 2203719 | C | T |  |  |  |  | control | NA | NA | SMG6 |
| 17 | 2203911 | A | G |  | Yes |  |  | case | NA | NA | SMG6 |
| 17 | 2203930 | G | C |  |  |  |  | both | 123.0(15.0) | 66.7 | SMG6 |
| 17 | 2206943 | T | C |  | Yes |  |  | case | 82.0(0.0) | 100 | SMG6 |
| 17 | 2206959 | G | A |  |  |  |  | control | NA | NA | SMG6 |
| 17 | 2218891 | C | G |  |  |  |  | control | NA | NA | SRR |
| 17 | 2218956 | C | G |  |  |  |  | control | NA | NA | SRR |
| 17 | 2219015 | A | C |  | Yes |  |  | case | 138.0(0.0) | NA | SRR |
| 17 | 2221258 | A | G |  | Yes |  |  | control | NA | NA | SRR |
| 17 | 2221290 | A | G |  | Yes |  |  | control | NA | NA | SRR |
| 17 | 2224640 | G | A |  |  |  |  | control | NA | NA | SRR |
| 17 | 2224846 | C | T |  |  |  |  | both | 76.4(40.4) | 50 | SRR |
| 17 | 2226543 | G | A |  |  |  |  | case | 136.2(0.0) | NA | SRR |
| 17 | 2227025 | C | T |  | Yes |  |  | control | NA | NA | SRR |
| 17 | 2227034 | G | A |  |  |  |  | case | 118.6(0.0) | NA | SRR |
| 19 | 10381855 | C | G |  |  |  |  | case | 113.8(0.0) | 33.3 | ICAM1 |
| 19 | 10381895 | T | G |  |  |  |  | control | NA | NA | ICAM1 |
| 19 | 10385456 | G | A |  |  |  |  | case | NA | 100 | ICAM1 |
| 19 | 10385517 | T | C |  |  |  |  | control | NA | NA | ICAM1 |
| 19 | 10385618 | G | GC |  | Yes | Yes |  | control | NA | NA | ICAM1 |
| 19 | 10385621 | G | A |  |  |  |  | case | 124.6(0.0) | NA | ICAM1 |
| 19 | 10385697 | T | C |  |  |  |  | case | 82.3(53.3) | 100 | ICAM1 |
| 19 | 10385698 | A | G |  |  |  |  | case | 97.6(0.0) | 100 | ICAM1 |
| 19 | 10394170 | A | G |  |  |  |  | case | NA | NA | ICAM1 |
| 19 | 10394206 | A | G |  |  |  |  | control | NA | NA | ICAM1 |
| 19 | 10394225 | A | C |  |  |  |  | case | 85.2(0.0) | NA | ICAM1 |
| 19 | 10394312 | C | G |  |  |  |  | control | NA | NA | ICAM1 |
| 19 | 10394373 | G | A |  |  |  |  | case | 111.1(16.5) | 50 | ICAM1 |
| 19 | 10394385 | A | G |  |  |  |  | control | NA | NA | ICAM1 |
| 19 | 10394438 | A | G |  |  |  |  | both | 120.8(21.1) | 40 | ICAM1 |
| 19 | 10394462 | T | G |  |  |  |  | case | NA | 100 | ICAM1 |
| 19 | 10394744 | T | C |  |  |  |  | case | 101.6(0.0) | NA | ICAM1 |
| 19 | 10394774 | A | G |  |  |  |  | case | NA | NA | ICAM1 |
| 19 | 10394808 | T | C |  |  |  |  | case | NA | NA | ICAM1 |
| 19 | 10394833 | T | G |  |  |  |  | case | 93.0(0.0) | 100 | ICAM1 |
| 19 | 10394842 | T | G |  |  |  |  | case | 127.0(0.0) | NA | ICAM1 |
| 19 | 10394912 | T | G |  | Yes | Yes |  | control | NA | NA | ICAM1 |
| 19 | 10395085 | T | C |  |  |  |  | case | NA | NA | ICAM1 |
| 19 | 10395088 | T | C |  |  |  |  | case | 151.8(0.0) | NA | ICAM1 |
| 19 | 10395135 | A | G |  |  |  |  | case | 61.4(0.0) | 100 | ICAM1 |
| 19 | 10395194 | T | C |  |  |  |  | control | NA | NA | ICAM1 |
| 19 | 10395251 | C | G |  |  |  |  | both | 144.2(0.0) | NA | ICAM1 |
| 19 | 10395268 | T | C |  |  |  |  | both | 87.2(0.0) | 100 | ICAM1 |
| 19 | 10395583 | T | C |  |  |  |  | control | NA | NA | ICAM1 |
| 19 | 10395585 | T | C |  |  |  |  | control | NA | NA | ICAM1 |
| 19 | 10395691 | T | C |  |  |  |  | case | 105.3(0.0) | NA | ICAM1 |
| 19 | 10395831 | T | C |  |  |  |  | control | NA | NA | ICAM1 |
| 19 | 10395832 | A | G |  |  |  |  | case | 44.6(0.0) | 100 | ICAM1 |
| 19 | 10395909 | G | A |  |  |  |  | control | NA | NA | ICAM1 |
| 19 | 11210902 | A | G |  |  |  |  | case | 87.4(0.0) | 100 | LDLR |
| 19 | 11210951 | T | C |  |  |  |  | case | 123.2(0.0) | NA | LDLR |
| 19 | 11210952 | A | T |  |  |  |  | both | 102.6(16.4) | 16.7 | LDLR |
| 19 | 11210970 | A | G | Yes | Yes |  | Sanger sequencing | case | 131.1(15.1) | 50 | LDLR |
| 19 | 11210982 | A | G |  | Yes |  | Sanger sequencing | control | NA | NA | LDLR |
| 19 | 11213346 | C | T |  |  |  |  | case | 77.0(11.3) | 40 | LDLR |
| 19 | 11213360 | A | G | Yes |  |  |  | case | 122.1(39.4) | 50 | LDLR |
| 19 | 11213389 | T | C |  |  |  |  | case | 76.8(0.0) | 100 | LDLR |
| 19 | 11213434 | A | C |  | Yes | Yes | Sanger sequencing | case | 206.0(0.0) | 100 | LDLR |
| 19 | 11215900 | G | C |  |  |  |  | case | 129.2(38.0) | 28.6 | LDLR |
| 19 | 11215918 | T | C |  |  |  |  | case | 124.3(18.8) | NA | LDLR |
| 19 | 11215943 | G | T |  | Yes |  | Sanger sequencing | case | 168.0(0.0) | NA | LDLR |
| 19 | 11215964 | T | TG |  | Yes | Yes | Sanger sequencing | case | 122.2(0.0) | 100 | LDLR |
| 19 | 11215970 | A | T |  |  |  |  | case | 98.8(55.5) | 50 | LDLR |
| 19 | 11215977 | A | G | Yes |  |  |  | both | 131.2(31.5) | 37.5 | LDLR |
| 19 | 11216000 | A | G |  | Yes |  | Sanger sequencing | control | 133.8(5.4) | 100 | LDLR |
| 19 | 11216007 | T | C |  |  |  |  | control | NA | NA | LDLR |
| 19 | 11216009 | G | T |  | Yes |  | Sanger sequencing | case | 144.8(0.0) | 100 | LDLR |
| 19 | 11216033 | A | G |  |  |  |  | case | 108.2(73.0) | 66.7 | LDLR |
| 19 | 11216113 | A | G |  |  |  |  | case | 131.6(0.0) | 100 | LDLR |
| 19 | 11216125 | A | G |  |  |  |  | case | 124.0(0.0) | 50 | LDLR |
| 19 | 11216147 | A | G |  |  |  |  | case | 130.8(0.0) | NA | LDLR |
| 19 | 11216242 | A | C |  |  |  |  | case | 83.2(0.0) | 100 | LDLR |
| 19 | 11216275 | T | C |  |  |  |  | control | 105.2(40.0) | 41.7 | LDLR |
| 19 | 11217255 | T | C |  |  |  |  | case | 111.4(0.0) | 100 | LDLR |
| 19 | 11217296 | C | T |  |  |  |  | control | NA | NA | LDLR |
| 19 | 11218074 | C | T |  |  |  |  | control | NA | NA | LDLR |
| 19 | 11221364 | A | C |  |  |  |  | case | 115.6(0.0) | 100 | LDLR |
| 19 | 11221389 | T | C |  |  |  |  | both | 122.5(33.2) | 50 | LDLR |
| 19 | 11221397 | G | A |  |  |  |  | case | 134.8(30.5) | 55.6 | LDLR |
| 19 | 11221399 | A | T |  | Yes |  | Sanger sequencing | both | 175.8(35.6) | 75 | LDLR |
| 19 | 11222190 | AT | A |  | Yes | Yes | Sanger sequencing | case | NA | 100 | LDLR |
| 19 | 11222192 | T | A |  | Yes |  | Sanger sequencing | case | 133.5(26.7) | 25 | LDLR |
| 19 | 11222213 | A | G |  |  |  |  | case | NA | 100 | LDLR |
| 19 | 11222265 | A | G |  | Yes |  | Sanger sequencing | case | 182.4(0.0) | 100 | LDLR |
| 19 | 11223954 | C | G |  |  |  |  | case | 123.7(0.0) | NA | LDLR |
| 19 | 11223962 | A | G | Yes |  |  |  | case | 155.0(0.0) | 100 | LDLR |
| 19 | 11223977 | G | A |  |  |  |  | case | 106.6(28.2) | 80 | LDLR |
| 19 | 11224006 | A | G |  |  |  |  | control | 108.3(22.6) | 11.1 | LDLR |
| 19 | 11224013 | T | C | Yes | Yes |  | Sanger sequencing | case | 154.8(0.0) | 100 | LDLR |
| 19 | 11224014 | A | G | Yes |  |  |  | case | 104.0(25.2) | 100 | LDLR |
| 19 | 11224017 | A | G |  |  |  |  | case | NA | NA | LDLR |
| 19 | 11224025 | G | A |  |  |  |  | both | 116.1(43.1) | 46.7 | LDLR |
| 19 | 11224051 | T | C |  |  |  |  | case | 132.2(50.3) | 57.1 | LDLR |
| 19 | 11224354 | T | C | Yes |  |  |  | both | 133.9(17.3) | 50 | LDLR |
| 19 | 11224434 | G | C |  |  |  |  | control | 114.9(23.9) | NA | LDLR |
| 19 | 11226883 | A | C |  |  |  |  | case | 144.6(0.0) | NA | LDLR |
| 19 | 11226885 | G | C |  |  |  |  | both | 149.8(47.5) | 64.9 | LDLR |
| 19 | 11227547 | A | G |  |  |  |  | control | NA | NA | LDLR |
| 19 | 11227593 | T | C |  |  |  |  | both | 123.8(32.1) | 40.7 | LDLR |
| 19 | 11227601 | G | A | Yes |  |  |  | case | 110.6(11.9) | 75 | LDLR |
| 19 | 11227647 | T | C |  |  |  |  | case | 171.6(38.8) | 66.7 | LDLR |
| 19 | 11227651 | T | C |  | Yes |  | Sanger sequencing | case | 171.6(38.8) | 66.7 | LDLR |
| 19 | 11227663 | T | G |  |  |  |  | case | 169.2(47.2) | 62.5 | LDLR |
| 19 | 11227676 | C | T |  | Yes | Yes | Sanger sequencing | case | 168.3(62.1) | 73.3 | LDLR |
| 19 | 11230785 | T | A |  |  |  |  | case | 141.4(0.0) | NA | LDLR |
| 19 | 11230791 | A | C |  |  |  |  | both | 131.7(24.6) | 16.7 | LDLR |
| 19 | 11230802 | T | C |  |  |  |  | case | 130.8(0.0) | 100 | LDLR |
| 19 | 11230869 | T | A |  |  |  |  | control | NA | NA | LDLR |
| 19 | 11231056 | C | G |  | Yes |  | Sanger sequencing | case | NA | 100 | LDLR |
| 19 | 11231084 | A | G | Yes | Yes |  | Sanger sequencing | case | NA | 33.3 | LDLR |
| 19 | 11231110 | T | C |  |  |  |  | both | 108.0(14.9) | 40 | LDLR |
| 19 | 11231146 | A | C |  | Yes | Yes | Sanger sequencing | case | 190.4(0.0) | 100 | LDLR |
| 19 | 11233931 | G | C |  |  |  |  | control | NA | NA | LDLR |
| 19 | 11233966 | T | C |  |  |  |  | case | NA | 100 | LDLR |
| 19 | 11233995 | A | G |  |  |  |  | control | NA | NA | LDLR |
| 19 | 11238705 | T | G |  |  |  |  | case | 161.6(0.0) | NA | LDLR |
| 19 | 11238713 | G | GAGA | Yes |  |  |  | both | NA | NA | LDLR |
| 19 | 11238721 | A | G |  |  |  |  | case | 97.6(11.1) | 50 | LDLR |
| 19 | 11240214 | GAGAA | G |  | Yes | Yes | Sanger sequencing | control | NA | NA | LDLR |
| 19 | 11240215 | G | GTC |  | Yes | Yes | Sanger sequencing | control | NA | NA | LDLR |
| 19 | 11240230 | T | A |  | Yes | Yes | Sanger sequencing | case | 140.2(47.3) | 100 | LDLR |
| 19 | 11240256 | T | C |  |  |  |  | case | 118.4(47.0) | 57.1 | LDLR |
| 19 | 11240277 | T | C |  |  |  |  | control | NA | 100 | LDLR |
| 19 | 11241957 | C | A |  |  |  |  | case | 94.8(0.0) | 100 | LDLR |
| 19 | 11241972 | G | C | Yes |  |  |  | both | 121.5(46.6) | 33.3 | LDLR |

We listed 1,235 novel variants identified in the discovery stage. * Also identified in the replication stage. †Confirmed by IGV snapshot or Sanger sequencing. ‡Variants were case-specific, control-specific or identified in both groups. §A percentage of the administration of cholesterol lowering medications among variants carriers. Abbreviations: Chr, chromosome; A1, minor allele; A2, major allele; NA, data not available

**Table S4. Association results of single-variant tests from the discovery stage**

| Chr | Position  (hg19) | rsID  (dbSNP138) | A1 | A2 | MAF in cases | MAF in controls | OR | *P* | Gene | Type | AA　change |
| --- | --- | --- | --- | --- | --- | --- | --- | --- | --- | --- | --- |
| 1 | 55505604 | - | A | G | 0.016 | 0.0079 | 2 | 8.6 x 10^-5^ | *PCSK9* | missense | E32K |
| 1 | 55509585 | rs151193009 | T | C | 0.0047 | 0.011 | 0.44 | 2.7 x 10^-4^ | *PCSK9* | missense | R93C |
| 2 | 203684462 | - | A | C | 0.0016 | 0.00034 | 4.8 | 0.033 | *ICA1L* | missense | D174Y |
| 2 | 203948147 | - | G | A | 0.0022 | 0.00034 | 6.4 | 5.9 x 10^-3^ | *NBEAL1* | missense | N297S |
| 2 | 203996741 | - | C | A | 0.0049 | 0.0083 | 0.59 | 0.028 | *NBEAL1* | missense | K1175Q |
| 2 | 204003431 | rs141142182 | C | T | 0.012 | 0.016 | 0.723 | 0.048 | *NBEAL1* | missense | I1574T |
| 2 | 204032045 | - | A | G | 0.0013 | 0 | NA | 6.2 x 10^-3^ | *NBEAL1* | missense | V1958I |
| 2 | 204131371 | - | C | G | 0 | 0.0013 | 0 | 8.0 x 10^-3^ | *CYP20A1* | missense | Q189H |
| 4 | 156715112 | - | T | A | 0.0013 | 0 | NA | 6.2 x 10^-3^ | *GUCY1B3* | missense | E200D |
| 6 | 161159625 | rs121918027 | A | G | 0.022 | 0.015 | 1.5 | 3.5 x 10^-3^ | *PLG* | missense | A620T |
| 9 | 21971043 | - | T | G | 0.00018 | 0.0013 | 0.13 | 0.04 | *CDKN2A* | missense | D105E |
| 11 | 103814218 | - | T | C | 0.0012 | 0.00017 | 7.5 | 0.034 | *PDGFD* | missense | R245Q |
| 12 | 111082836 | rs118096349 | T | G | 0.0072 | 0.011 | 0.64 | 0.031 | *TCTN1* | missense | G466C |
| 17 | 1989027 | rs187319098 | G | C | 0.002 | 0.0046 | 0.43 | 0.022 | *SMG6* | missense | E268Q |
| 17 | 2186947 | rs372239404 | G | A | 0.0016 | 0.00017 | 9.6 | 9.5 x 10^-3^ | *SMG6* | missense | M807T |
| 19 | 11226885 | - | G | C | 0.0016 | 0.00017 | 9.6 | 9.5 x 10^-3^ | *LDLR* | missense | L568V |

All single-variant association results that *P* values were less than 0.05. Abbreviations: Chr, chromosome; A1, minor allele; A2, major allele; MAF, minor allele frequency; OR, odds ratio; AA, amino acid; NA, not available.

**Table S5. Association results of gene-based tests from the discovery stage**

| Genes | Non-Synonymous  (SKAT) | Damaging  (CAST) | Disruptive  (CAST) |
| --- | --- | --- | --- |
| *PCSK9* | 3.7 x 10^-5^ | 0.67 | 0.69 |
| *GUCY1B3* | 7.3 x 10^-3^ | 0.033 | 0.055 |
| *PLG* | 0.017 | 1 | 1 |
| *ICA1L* | 0.039 | 0.018 | NA |
| *NBEAL1* | 0.043 | 1 | 0.84 |
| *TCTN1* | 0.044 | 1 | NA |
| *LDLR* | 0.049 | 2.6 x 10^-5^ | 5.0 x 10^-3^ |

Genes which exceed a threshold of *P* < 0.05 at least in one test.

**Table S6. List of variants identified in the replication stage.**

| CHR | BP | A1 | A2 | dbSNP* | Discovery† | Damaging | Disruptive | case/control‡ | gene |
| --- | --- | --- | --- | --- | --- | --- | --- | --- | --- |
| 1 | 55505520 | A | G | rs186669805 | Yes |  |  | both | PCSK9 |
| 1 | 55505532 | G | C | novel |  |  |  | control | PCSK9 |
| 1 | 55505552 | ACTG | A | rs113330492 | Yes |  |  | both | PCSK9 |
| 1 | 55505555 | C | G | novel |  | Yes |  | control | PCSK9 |
| 1 | 55505581 | A | G | novel |  |  |  | both | PCSK9 |
| 1 | 55505596 | A | G | novel |  |  |  | case | PCSK9 |
| 1 | 55505601 | A | C | novel |  |  |  | case | PCSK9 |
| 1 | 55505604 | A | G | novel | Yes |  |  | both | PCSK9 |
| 1 | 55505625 | A | G | novel |  |  |  | case | PCSK9 |
| 1 | 55505668 | T | C | rs11583680 | Yes |  |  | both | PCSK9 |
| 1 | 55505671 | C | A | novel | Yes |  |  | both | PCSK9 |
| 1 | 55505684 | A | C | novel |  |  |  | case | PCSK9 |
| 1 | 55505692 | C | CA | novel |  | Yes | Yes | case | PCSK9 |
| 1 | 55505712 | A | G | novel | Yes |  |  | both | PCSK9 |
| 1 | 55509520 | T | C | novel | Yes |  |  | control | PCSK9 |
| 1 | 55509521 | A | G | novel |  | Yes |  | case | PCSK9 |
| 1 | 55509543 | A | G | novel | Yes |  |  | case | PCSK9 |
| 1 | 55509561 | A | G | novel | Yes |  |  | both | PCSK9 |
| 1 | 55509566 | T | C | novel |  | Yes |  | control | PCSK9 |
| 1 | 55509575 | A | G | rs373551845 |  | Yes |  | case | PCSK9 |
| 1 | 55509577 | G | A | novel |  |  |  | case | PCSK9 |
| 1 | 55509582 | C | G | novel |  |  |  | case | PCSK9 |
| 1 | 55509585 | T | C | rs151193009 | Yes |  |  | both | PCSK9 |
| 1 | 55509594 | T | C | rs185392267 | Yes |  |  | control | PCSK9 |
| 1 | 55509598 | A | G | rs376385276 |  |  |  | control | PCSK9 |
| 1 | 55509618 | T | C | rs369067856 |  |  |  | case | PCSK9 |
| 1 | 55509641 | T | C | novel |  | Yes |  | case | PCSK9 |
| 1 | 55509644 | A | G | rs79805678 |  | Yes |  | both | PCSK9 |
| 1 | 55509648 | A | G | novel |  |  |  | control | PCSK9 |
| 1 | 55509661 | C | T | novel |  |  |  | both | PCSK9 |
| 1 | 55509704 | C | G | novel | Yes |  |  | case | PCSK9 |
| 1 | 55512254 | A | G | novel |  |  |  | control | PCSK9 |
| 1 | 55512299 | T | C | novel | Yes |  |  | both | PCSK9 |
| 1 | 55517952 | T | C | rs148612296 |  | Yes |  | control | PCSK9 |
| 1 | 55517990 | T | G | novel |  | Yes |  | case | PCSK9 |
| 1 | 55518006 | A | C | novel |  | Yes |  | case | PCSK9 |
| 1 | 55518015 | T | C | novel |  | Yes |  | control | PCSK9 |
| 1 | 55518036 | T | C | rs200856421 |  | Yes |  | both | PCSK9 |
| 1 | 55518063 | T | C | novel |  | Yes |  | both | PCSK9 |
| 1 | 55518071 | A | G | novel |  |  |  | case | PCSK9 |
| 1 | 55518082 | G | C | novel | Yes |  |  | both | PCSK9 |
| 1 | 55518320 | T | C | novel |  | Yes |  | case | PCSK9 |
| 1 | 55518329 | G | A | novel |  |  |  | control | PCSK9 |
| 1 | 55518362 | T | G | rs150169598 |  |  |  | case | PCSK9 |
| 1 | 55518374 | T | C | rs148195424 | Yes |  |  | control | PCSK9 |
| 1 | 55518375 | T | G | novel |  |  |  | control | PCSK9 |
| 1 | 55518417 | A | G | rs376945520 |  |  |  | case | PCSK9 |
| 1 | 55518452 | A | G | rs200146448 | Yes |  |  | both | PCSK9 |
| 1 | 55518456 | T | C | rs201789841 | Yes |  |  | both | PCSK9 |
| 1 | 55518467 | G | A | rs2495477 |  | Yes |  | both | PCSK9 |
| 1 | 55521683 | G | A | novel |  |  |  | control | PCSK9 |
| 1 | 55521716 | A | G | novel |  |  |  | case | PCSK9 |
| 1 | 55521781 | A | G | novel |  | Yes |  | control | PCSK9 |
| 1 | 55521794 | A | G | novel |  |  |  | both | PCSK9 |
| 1 | 55521821 | A | C | novel |  | Yes |  | control | PCSK9 |
| 1 | 55523033 | A | G | rs509504 |  | Yes |  | case | PCSK9 |
| 1 | 55523034 | A | G | novel |  | Yes |  | control | PCSK9 |
| 1 | 55523076 | T | C | rs148562777 |  |  |  | control | PCSK9 |
| 1 | 55523099 | G | A | novel |  | Yes |  | control | PCSK9 |
| 1 | 55523119 | T | C | novel |  |  |  | case | PCSK9 |
| 1 | 55523126 | T | C | rs139683719 |  | Yes |  | both | PCSK9 |
| 1 | 55523141 | G | C | novel |  | Yes |  | control | PCSK9 |
| 1 | 55523188 | A | G | novel |  | Yes | Yes | control | PCSK9 |
| 1 | 55523798 | G | A | novel | Yes |  |  | both | PCSK9 |
| 1 | 55523812 | A | G | novel |  | Yes | Yes | both | PCSK9 |
| 1 | 55523829 | A | G | novel |  |  |  | case | PCSK9 |
| 1 | 55523873 | T | C | novel |  |  |  | control | PCSK9 |
| 1 | 55524197 | A | G | rs540796 |  | Yes |  | both | PCSK9 |
| 1 | 55524203 | T | A | novel |  | Yes |  | case | PCSK9 |
| 1 | 55524237 | G | A | rs562556 | Yes |  |  | both | PCSK9 |
| 1 | 55524239 | T | C | rs373517174 |  | Yes |  | control | PCSK9 |
| 1 | 55524244 | A | G | rs376388695 |  |  |  | case | PCSK9 |
| 1 | 55524245 | T | C | novel |  | Yes |  | control | PCSK9 |
| 1 | 55524300 | T | C | novel | Yes |  |  | control | PCSK9 |
| 1 | 55524312 | T | C | rs201395805 | Yes |  |  | both | PCSK9 |
| 1 | 55525165 | T | G | rs374455190 |  |  |  | both | PCSK9 |
| 1 | 55525183 | T | C | novel |  |  |  | case | PCSK9 |
| 1 | 55525195 | A | G | novel | Yes |  |  | both | PCSK9 |
| 1 | 55525219 | A | G | novel | Yes | Yes |  | both | PCSK9 |
| 1 | 55525241 | G | T | novel |  |  |  | case | PCSK9 |
| 1 | 55525261 | A | G | novel |  |  |  | control | PCSK9 |
| 1 | 55525295 | T | G | novel |  |  |  | control | PCSK9 |
| 1 | 55525335 | T | A | novel |  | Yes |  | control | PCSK9 |
| 1 | 55527062 | A | T | novel |  |  |  | case | PCSK9 |
| 1 | 55527093 | T | C | rs72646525 | Yes |  |  | both | PCSK9 |
| 1 | 55527110 | T | C | rs373323910 |  | Yes | Yes | case | PCSK9 |
| 1 | 55527131 | A | G | rs372586224 | Yes |  |  | both | PCSK9 |
| 1 | 55527141 | C | G | novel |  |  |  | both | PCSK9 |
| 1 | 55527158 | A | G | rs367606156 | Yes |  |  | both | PCSK9 |
| 1 | 55527174 | G | C | novel |  |  |  | both | PCSK9 |
| 1 | 55527190 | C | CAA | novel |  | Yes | Yes | control | PCSK9 |
| 1 | 55529048 | A | G | novel |  |  |  | both | PCSK9 |
| 1 | 55529056 | T | C | rs199815786 |  | Yes |  | both | PCSK9 |
| 1 | 55529064 | A | G | novel | Yes | Yes |  | both | PCSK9 |
| 1 | 55529108 | A | G | rs143291739 | Yes |  |  | both | PCSK9 |
| 1 | 55529125 | T | C | novel |  | Yes |  | both | PCSK9 |
| 1 | 55529132 | G | A | rs201280059 |  |  |  | both | PCSK9 |
| 1 | 55529153 | G | C | novel | Yes |  |  | both | PCSK9 |
| 1 | 55529158 | T | C | rs371914056 |  | Yes |  | control | PCSK9 |
| 1 | 55529182 | A | C | novel | Yes |  |  | both | PCSK9 |
| 1 | 55529187 | G | A | rs505151 | Yes |  |  | both | PCSK9 |
| 1 | 55529200 | T | C | novel |  | Yes |  | control | PCSK9 |
| 1 | 55529243 | T | C | novel |  | Yes | Yes | control | PCSK9 |
| 2 | 203644300 | T | G | novel |  |  |  | case | ICA1L |
| 2 | 203644364 | G | GT | novel |  | Yes | Yes | control | ICA1L |
| 2 | 203650717 | C | T | novel |  | Yes |  | control | ICA1L |
| 2 | 203653562 | T | C | novel | Yes |  |  | both | ICA1L |
| 2 | 203653569 | A | C | novel |  | Yes |  | both | ICA1L |
| 2 | 203653587 | T | A | rs142998309 |  |  |  | case | ICA1L |
| 2 | 203653611 | C | A | novel |  | Yes |  | case | ICA1L |
| 2 | 203653622 | T | C | novel | Yes |  |  | both | ICA1L |
| 2 | 203653775 | T | C | novel |  |  |  | control | ICA1L |
| 2 | 203661666 | C | T | novel |  |  |  | both | ICA1L |
| 2 | 203679478 | G | A | novel |  | Yes |  | both | ICA1L |
| 2 | 203679493 | T | C | novel |  | Yes |  | case | ICA1L |
| 2 | 203679496 | A | G | novel |  | Yes |  | case | ICA1L |
| 2 | 203680729 | C | T | novel |  |  |  | case | ICA1L |
| 2 | 203682193 | C | A | novel |  | Yes | Yes | both | ICA1L |
| 2 | 203682229 | A | T | novel |  | Yes |  | case | ICA1L |
| 2 | 203684462 | A | C | novel | Yes | Yes |  | both | ICA1L |
| 2 | 203684601 | C | CAG | novel |  | Yes | Yes | control | ICA1L |
| 2 | 203684617 | A | G | novel | Yes |  |  | both | ICA1L |
| 2 | 203690442 | T | C | novel |  |  |  | case | ICA1L |
| 2 | 203690443 | A | G | novel |  | Yes |  | control | ICA1L |
| 2 | 203693607 | G | C | novel |  |  |  | control | ICA1L |
| 2 | 203693671 | C | T | novel |  |  |  | case | ICA1L |
| 2 | 203693674 | G | T | novel |  | Yes |  | control | ICA1L |
| 2 | 203693715 | G | T | novel |  |  |  | case | ICA1L |
| 2 | 203881142 | G | T | novel |  |  |  | case | NBEAL1 |
| 2 | 203881143 | A | G | novel |  |  |  | control | NBEAL1 |
| 2 | 203881152 | C | T | novel |  | Yes |  | control | NBEAL1 |
| 2 | 203906514 | C | G | novel |  |  |  | case | NBEAL1 |
| 2 | 203906522 | C | CTT | novel |  | Yes | Yes | case | NBEAL1 |
| 2 | 203906537 | G | A | novel |  |  |  | case | NBEAL1 |
| 2 | 203906553 | T | C | novel |  | Yes |  | both | NBEAL1 |
| 2 | 203914554 | T | C | novel |  |  |  | case | NBEAL1 |
| 2 | 203914634 | C | G | novel |  |  |  | control | NBEAL1 |
| 2 | 203914652 | T | C | novel |  | Yes | Yes | both | NBEAL1 |
| 2 | 203914694 | C | T | novel | Yes |  |  | both | NBEAL1 |
| 2 | 203914695 | T | G | novel |  |  |  | control | NBEAL1 |
| 2 | 203921185 | T | C | novel |  |  |  | case | NBEAL1 |
| 2 | 203921186 | A | G | novel |  | Yes |  | case | NBEAL1 |
| 2 | 203922075 | G | A | rs146121994 |  | Yes |  | case | NBEAL1 |
| 2 | 203922079 | A | G | novel |  |  |  | case | NBEAL1 |
| 2 | 203922103 | C | T | novel |  |  |  | case | NBEAL1 |
| 2 | 203922115 | T | G | novel |  |  |  | control | NBEAL1 |
| 2 | 203922161 | T | G | novel |  |  |  | case | NBEAL1 |
| 2 | 203922165 | C | T | novel |  | Yes |  | control | NBEAL1 |
| 2 | 203942551 | T | C | novel |  | Yes |  | control | NBEAL1 |
| 2 | 203942552 | A | G | novel | Yes |  |  | control | NBEAL1 |
| 2 | 203947955 | G | A | novel | Yes |  |  | case | NBEAL1 |
| 2 | 203947966 | T | C | novel |  |  |  | case | NBEAL1 |
| 2 | 203948014 | T | TG | novel |  | Yes | Yes | both | NBEAL1 |
| 2 | 203948044 | G | A | novel | Yes |  |  | both | NBEAL1 |
| 2 | 203948050 | A | G | novel | Yes |  |  | case | NBEAL1 |
| 2 | 203948076 | C | A | novel |  | Yes |  | case | NBEAL1 |
| 2 | 203948147 | G | A | novel | Yes |  |  | both | NBEAL1 |
| 2 | 203948198 | A | G | novel |  |  |  | case | NBEAL1 |
| 2 | 203948203 | G | A | novel |  |  |  | control | NBEAL1 |
| 2 | 203948218 | T | C | novel |  | Yes | Yes | control | NBEAL1 |
| 2 | 203949204 | G | T | novel |  | Yes | Yes | both | NBEAL1 |
| 2 | 203949210 | A | G | novel |  |  |  | case | NBEAL1 |
| 2 | 203949256 | A | C | novel |  | Yes | Yes | case | NBEAL1 |
| 2 | 203964355 | C | T | novel | Yes |  |  | both | NBEAL1 |
| 2 | 203964406 | A | G | rs116202043 |  |  |  | control | NBEAL1 |
| 2 | 203964417 | G | C | novel |  |  |  | case | NBEAL1 |
| 2 | 203972147 | C | T | novel |  |  |  | control | NBEAL1 |
| 2 | 203972193 | G | A | novel |  | Yes |  | case | NBEAL1 |
| 2 | 203972196 | G | C | novel |  | Yes |  | control | NBEAL1 |
| 2 | 203972351 | C | T | novel |  | Yes |  | control | NBEAL1 |
| 2 | 203972384 | G | A | novel |  | Yes |  | case | NBEAL1 |
| 2 | 203972385 | G | C | novel | Yes |  |  | case | NBEAL1 |
| 2 | 203972408 | A | G | novel |  | Yes | Yes | case | NBEAL1 |
| 2 | 203972425 | G | C | rs183416448 |  |  |  | control | NBEAL1 |
| 2 | 203972465 | G | A | novel |  | Yes |  | case | NBEAL1 |
| 2 | 203972568 | G | T | novel |  |  |  | control | NBEAL1 |
| 2 | 203972603 | A | G | novel |  | Yes |  | case | NBEAL1 |
| 2 | 203972608 | A | G | novel |  |  |  | case | NBEAL1 |
| 2 | 203972647 | A | G | novel |  |  |  | control | NBEAL1 |
| 2 | 203972696 | T | C | novel |  | Yes |  | case | NBEAL1 |
| 2 | 203972703 | T | C | novel |  | Yes | Yes | both | NBEAL1 |
| 2 | 203972725 | A | G | novel |  |  |  | case | NBEAL1 |
| 2 | 203972761 | G | A | novel | Yes |  |  | case | NBEAL1 |
| 2 | 203972853 | T | C | novel |  | Yes | Yes | control | NBEAL1 |
| 2 | 203974917 | G | A | novel |  |  |  | case | NBEAL1 |
| 2 | 203974934 | A | G | novel |  |  |  | case | NBEAL1 |
| 2 | 203974965 | T | C | rs181724607 |  |  |  | case | NBEAL1 |
| 2 | 203974966 | A | G | rs199945717 |  | Yes |  | both | NBEAL1 |
| 2 | 203974986 | C | G | novel |  |  |  | control | NBEAL1 |
| 2 | 203976724 | T | TG | novel |  | Yes | Yes | case | NBEAL1 |
| 2 | 203976760 | G | A | novel |  |  |  | control | NBEAL1 |
| 2 | 203977747 | C | T | novel |  |  |  | case | NBEAL1 |
| 2 | 203977775 | A | G | novel |  |  |  | control | NBEAL1 |
| 2 | 203977823 | T | C | novel |  |  |  | both | NBEAL1 |
| 2 | 203977840 | T | C | rs369275951 | Yes |  |  | case | NBEAL1 |
| 2 | 203977841 | A | G | novel |  |  |  | case | NBEAL1 |
| 2 | 203977918 | G | A | novel | Yes |  |  | case | NBEAL1 |
| 2 | 203977932 | A | C | novel |  | Yes |  | control | NBEAL1 |
| 2 | 203977966 | C | G | novel | Yes |  |  | control | NBEAL1 |
| 2 | 203977990 | C | A | novel |  |  |  | control | NBEAL1 |
| 2 | 203977991 | C | T | novel | Yes |  |  | case | NBEAL1 |
| 2 | 203978006 | C | T | novel |  |  |  | case | NBEAL1 |
| 2 | 203978008 | T | C | novel |  | Yes | Yes | both | NBEAL1 |
| 2 | 203980717 | T | G | novel |  |  |  | case | NBEAL1 |
| 2 | 203980718 | A | T | novel |  | Yes | Yes | case | NBEAL1 |
| 2 | 203980733 | GCCCA | G | novel |  | Yes | Yes | case | NBEAL1 |
| 2 | 203980750 | C | T | novel |  |  |  | case | NBEAL1 |
| 2 | 203987004 | G | T | novel |  |  |  | control | NBEAL1 |
| 2 | 203987050 | C | T | novel |  |  |  | control | NBEAL1 |
| 2 | 203987059 | T | A | novel | Yes |  |  | both | NBEAL1 |
| 2 | 203990076 | G | A | novel |  |  |  | case | NBEAL1 |
| 2 | 203990094 | A | G | novel | Yes |  |  | both | NBEAL1 |
| 2 | 203990097 | C | G | novel |  |  |  | case | NBEAL1 |
| 2 | 203990134 | A | C | novel |  |  |  | case | NBEAL1 |
| 2 | 203990157 | T | C | novel | Yes |  |  | both | NBEAL1 |
| 2 | 203990158 | G | T | novel |  | Yes |  | case | NBEAL1 |
| 2 | 203990772 | C | A | novel |  |  |  | case | NBEAL1 |
| 2 | 203990773 | C | T | rs78750758 | Yes |  |  | both | NBEAL1 |
| 2 | 203990816 | A | G | novel |  | Yes |  | both | NBEAL1 |
| 2 | 203991287 | A | G | novel | Yes |  |  | both | NBEAL1 |
| 2 | 203991339 | G | A | novel |  | Yes |  | control | NBEAL1 |
| 2 | 203991361 | A | C | novel | Yes |  |  | both | NBEAL1 |
| 2 | 203991404 | A | G | novel |  |  |  | both | NBEAL1 |
| 2 | 203991436 | G | A | novel |  |  |  | control | NBEAL1 |
| 2 | 203991439 | A | G | novel |  |  |  | case | NBEAL1 |
| 2 | 203991566 | G | A | novel |  | Yes |  | case | NBEAL1 |
| 2 | 203991597 | T | C | novel |  | Yes | Yes | case | NBEAL1 |
| 2 | 203991607 | G | A | novel |  |  |  | control | NBEAL1 |
| 2 | 203992530 | T | C | novel |  |  |  | case | NBEAL1 |
| 2 | 203992533 | C | T | novel |  |  |  | case | NBEAL1 |
| 2 | 203992544 | T | C | novel |  | Yes | Yes | case | NBEAL1 |
| 2 | 203992579 | T | A | rs185067409 | Yes |  |  | both | NBEAL1 |
| 2 | 203992638 | A | C | novel |  |  |  | case | NBEAL1 |
| 2 | 203992640 | A | G | novel |  |  |  | control | NBEAL1 |
| 2 | 203995077 | T | C | novel |  |  |  | control | NBEAL1 |
| 2 | 203995078 | A | G | rs189753012 |  |  |  | case | NBEAL1 |
| 2 | 203995083 | T | A | novel |  |  |  | case | NBEAL1 |
| 2 | 203995111 | A | T | novel | Yes |  |  | both | NBEAL1 |
| 2 | 203995131 | A | G | novel |  |  |  | case | NBEAL1 |
| 2 | 203995146 | A | G | rs376644554 | Yes |  |  | both | NBEAL1 |
| 2 | 203995148 | C | A | novel |  | Yes |  | both | NBEAL1 |
| 2 | 203996711 | C | T | novel |  | Yes |  | control | NBEAL1 |
| 2 | 203996726 | A | G | novel | Yes |  |  | case | NBEAL1 |
| 2 | 203996741 | C | A | novel | Yes |  |  | both | NBEAL1 |
| 2 | 203996742 | G | A | novel |  |  |  | case | NBEAL1 |
| 2 | 203996776 | A | G | novel |  | Yes |  | case | NBEAL1 |
| 2 | 203996792 | G | C | novel | Yes |  |  | both | NBEAL1 |
| 2 | 203996817 | T | C | novel |  |  |  | control | NBEAL1 |
| 2 | 203996846 | G | A | novel |  |  |  | case | NBEAL1 |
| 2 | 203997831 | G | A | novel | Yes |  |  | both | NBEAL1 |
| 2 | 204000408 | G | A | rs189175469 |  | Yes |  | case | NBEAL1 |
| 2 | 204000445 | T | TC | novel |  | Yes | Yes | case | NBEAL1 |
| 2 | 204000509 | A | G | novel |  |  |  | case | NBEAL1 |
| 2 | 204000515 | T | C | rs199672188 |  |  |  | case | NBEAL1 |
| 2 | 204000584 | G | A | novel | Yes |  |  | control | NBEAL1 |
| 2 | 204000613 | GA | G | novel |  | Yes | Yes | case | NBEAL1 |
| 2 | 204000623 | T | C | novel |  |  |  | control | NBEAL1 |
| 2 | 204000661 | C | A | rs201246207 | Yes |  |  | both | NBEAL1 |
| 2 | 204000670 | T | G | novel | Yes |  |  | both | NBEAL1 |
| 2 | 204000759 | G | A | rs199629992 |  | Yes |  | both | NBEAL1 |
| 2 | 204000893 | A | T | novel | Yes |  |  | both | NBEAL1 |
| 2 | 204000935 | G | T | novel |  |  |  | control | NBEAL1 |
| 2 | 204001326 | A | G | novel |  |  |  | case | NBEAL1 |
| 2 | 204001418 | T | C | novel |  | Yes | Yes | control | NBEAL1 |
| 2 | 204001423 | C | A | novel |  | Yes |  | control | NBEAL1 |
| 2 | 204001437 | T | C | novel |  |  |  | case | NBEAL1 |
| 2 | 204001497 | T | C | novel |  |  |  | case | NBEAL1 |
| 2 | 204002892 | T | C | novel |  | Yes | Yes | case | NBEAL1 |
| 2 | 204002963 | C | CT | novel |  | Yes | Yes | control | NBEAL1 |
| 2 | 204002967 | T | C | novel |  | Yes | Yes | case | NBEAL1 |
| 2 | 204002968 | T | G | novel |  |  |  | case | NBEAL1 |
| 2 | 204002977 | C | T | novel |  |  |  | case | NBEAL1 |
| 2 | 204002989 | G | A | novel | Yes |  |  | case | NBEAL1 |
| 2 | 204003368 | T | TTGAC | novel |  | Yes | Yes | case | NBEAL1 |
| 2 | 204003381 | C | A | novel |  | Yes |  | both | NBEAL1 |
| 2 | 204003431 | C | T | rs141142182 | Yes |  |  | both | NBEAL1 |
| 2 | 204003432 | A | C | novel |  | Yes |  | case | NBEAL1 |
| 2 | 204003465 | G | T | novel | Yes |  |  | case | NBEAL1 |
| 2 | 204009332 | G | A | novel | Yes |  |  | control | NBEAL1 |
| 2 | 204009364 | T | G | novel |  |  |  | case | NBEAL1 |
| 2 | 204009369 | G | A | rs150123071 | Yes |  |  | both | NBEAL1 |
| 2 | 204009386 | A | G | novel |  |  |  | case | NBEAL1 |
| 2 | 204009437 | G | A | rs182529739 |  |  |  | case | NBEAL1 |
| 2 | 204009482 | T | C | novel |  | Yes |  | case | NBEAL1 |
| 2 | 204009502 | C | T | novel |  | Yes |  | control | NBEAL1 |
| 2 | 204009527 | T | C | novel |  |  |  | case | NBEAL1 |
| 2 | 204009532 | G | A | novel |  | Yes |  | case | NBEAL1 |
| 2 | 204009553 | C | T | novel |  | Yes |  | case | NBEAL1 |
| 2 | 204009618 | C | T | novel |  |  |  | case | NBEAL1 |
| 2 | 204009775 | T | C | novel |  | Yes |  | control | NBEAL1 |
| 2 | 204009790 | T | C | novel |  | Yes |  | control | NBEAL1 |
| 2 | 204009836 | G | A | novel |  |  |  | case | NBEAL1 |
| 2 | 204009845 | T | C | novel |  | Yes | Yes | control | NBEAL1 |
| 2 | 204009846 | A | G | novel |  |  |  | both | NBEAL1 |
| 2 | 204009881 | T | C | novel |  | Yes | Yes | both | NBEAL1 |
| 2 | 204016221 | C | T | novel |  | Yes |  | both | NBEAL1 |
| 2 | 204016266 | C | T | rs146950181 |  | Yes |  | both | NBEAL1 |
| 2 | 204016280 | C | A | novel |  | Yes |  | case | NBEAL1 |
| 2 | 204016303 | A | G | novel |  |  |  | case | NBEAL1 |
| 2 | 204022453 | G | A | novel |  | Yes |  | control | NBEAL1 |
| 2 | 204022478 | T | G | rs76455076 | Yes |  |  | both | NBEAL1 |
| 2 | 204022498 | A | G | novel |  | Yes |  | both | NBEAL1 |
| 2 | 204022523 | C | G | novel | Yes |  |  | both | NBEAL1 |
| 2 | 204031974 | T | C | novel |  |  |  | control | NBEAL1 |
| 2 | 204031991 | C | G | novel |  |  |  | control | NBEAL1 |
| 2 | 204032009 | TA | T | novel |  | Yes | Yes | case | NBEAL1 |
| 2 | 204032011 | T | C | novel |  | Yes |  | both | NBEAL1 |
| 2 | 204032045 | A | G | novel | Yes |  |  | both | NBEAL1 |
| 2 | 204034492 | A | G | novel | Yes |  |  | control | NBEAL1 |
| 2 | 204034502 | G | A | novel |  | Yes |  | both | NBEAL1 |
| 2 | 204034542 | T | C | novel |  | Yes | Yes | both | NBEAL1 |
| 2 | 204034572 | T | C | novel |  | Yes | Yes | control | NBEAL1 |
| 2 | 204036668 | A | G | novel |  | Yes |  | control | NBEAL1 |
| 2 | 204036670 | G | T | novel |  | Yes |  | control | NBEAL1 |
| 2 | 204037480 | T | C | novel | Yes | Yes |  | case | NBEAL1 |
| 2 | 204037488 | G | T | novel |  | Yes |  | case | NBEAL1 |
| 2 | 204037527 | C | A | novel | Yes | Yes |  | case | NBEAL1 |
| 2 | 204037533 | G | A | rs140473940 |  |  |  | case | NBEAL1 |
| 2 | 204037556 | T | C | rs201436178 |  | Yes |  | case | NBEAL1 |
| 2 | 204039973 | T | C | novel |  | Yes |  | case | NBEAL1 |
| 2 | 204039974 | T | C | rs372557211 |  | Yes |  | case | NBEAL1 |
| 2 | 204039975 | A | G | novel |  | Yes |  | control | NBEAL1 |
| 2 | 204045118 | A | G | novel |  | Yes |  | both | NBEAL1 |
| 2 | 204045122 | A | G | rs368629458 |  | Yes |  | case | NBEAL1 |
| 2 | 204045126 | A | G | rs201582714 |  | Yes |  | case | NBEAL1 |
| 2 | 204048059 | T | C | novel | Yes | Yes |  | both | NBEAL1 |
| 2 | 204053211 | T | C | novel |  | Yes |  | case | NBEAL1 |
| 2 | 204053262 | T | G | novel |  | Yes |  | case | NBEAL1 |
| 2 | 204053282 | T | C | novel |  |  |  | control | NBEAL1 |
| 2 | 204053289 | A | T | novel | Yes | Yes |  | control | NBEAL1 |
| 2 | 204058523 | C | A | novel |  | Yes |  | control | NBEAL1 |
| 2 | 204058557 | G | C | rs114408639 |  |  |  | case | NBEAL1 |
| 2 | 204058588 | C | T | novel | Yes |  |  | control | NBEAL1 |
| 2 | 204058614 | G | C | novel | Yes |  |  | both | NBEAL1 |
| 2 | 204062060 | A | C | novel |  | Yes |  | both | NBEAL1 |
| 2 | 204062061 | A | G | rs4675323 | Yes |  |  | both | NBEAL1 |
| 2 | 204062079 | T | C | novel |  |  |  | control | NBEAL1 |
| 2 | 204062083 | T | C | novel |  |  |  | both | NBEAL1 |
| 2 | 204064149 | C | A | novel |  |  |  | case | NBEAL1 |
| 2 | 204066326 | C | T | novel |  | Yes |  | case | NBEAL1 |
| 2 | 204066367 | G | A | novel |  |  |  | case | NBEAL1 |
| 2 | 204066402 | G | A | novel | Yes |  |  | both | NBEAL1 |
| 2 | 204066416 | G | C | novel | Yes |  |  | both | NBEAL1 |
| 2 | 204066426 | T | A | novel |  |  |  | case | NBEAL1 |
| 2 | 204066429 | T | C | rs201080847 |  |  |  | both | NBEAL1 |
| 2 | 204066437 | A | G | rs201986939 | Yes |  |  | both | NBEAL1 |
| 2 | 204067474 | C | T | novel |  | Yes |  | case | NBEAL1 |
| 2 | 204067479 | T | G | novel |  | Yes |  | case | NBEAL1 |
| 2 | 204067494 | T | TAACAC | novel |  | Yes | Yes | case | NBEAL1 |
| 2 | 204073411 | A | C | rs200435235 | Yes | Yes |  | control | NBEAL1 |
| 2 | 204073414 | G | A | rs140112414 | Yes |  |  | case | NBEAL1 |
| 2 | 204073415 | T | C | novel |  |  |  | control | NBEAL1 |
| 2 | 204073419 | T | C | novel |  | Yes |  | both | NBEAL1 |
| 2 | 204073420 | A | G | novel |  |  |  | case | NBEAL1 |
| 2 | 204073430 | C | G | rs369316763 | Yes |  |  | control | NBEAL1 |
| 2 | 204073472 | T | C | novel | Yes |  |  | case | NBEAL1 |
| 2 | 204073473 | G | A | novel |  | Yes |  | case | NBEAL1 |
| 2 | 204073891 | T | C | novel | Yes |  |  | case | NBEAL1 |
| 2 | 204073938 | T | C | novel |  | Yes | Yes | case | NBEAL1 |
| 2 | 204073939 | A | G | novel | Yes |  |  | control | NBEAL1 |
| 2 | 204073953 | C | A | novel | Yes |  |  | case | NBEAL1 |
| 2 | 204073966 | G | T | novel |  |  |  | control | NBEAL1 |
| 2 | 204073971 | G | A | novel |  |  |  | case | NBEAL1 |
| 2 | 204074020 | A | G | rs145550746 | Yes |  |  | both | NBEAL1 |
| 2 | 204075760 | C | T | novel |  |  |  | case | NBEAL1 |
| 2 | 204075770 | T | C | rs370994169 |  | Yes |  | case | NBEAL1 |
| 2 | 204075771 | A | G | rs200542429 | Yes |  |  | case | NBEAL1 |
| 2 | 204078242 | C | T | novel |  | Yes |  | control | NBEAL1 |
| 2 | 204078253 | T | C | novel |  | Yes |  | control | NBEAL1 |
| 2 | 204078273 | A | G | novel |  |  |  | control | NBEAL1 |
| 2 | 204078278 | T | C | novel | Yes |  |  | both | NBEAL1 |
| 2 | 204078290 | C | G | novel |  |  |  | control | NBEAL1 |
| 2 | 204078325 | G | A | novel |  | Yes |  | control | NBEAL1 |
| 2 | 204081997 | G | C | novel |  | Yes |  | case | NBEAL1 |
| 2 | 204082014 | A | G | rs180757088 |  | Yes |  | both | NBEAL1 |
| 2 | 204082021 | T | C | rs377175460 |  | Yes |  | control | NBEAL1 |
| 2 | 204082035 | A | T | novel |  | Yes |  | control | NBEAL1 |
| 2 | 204082063 | G | C | novel |  |  |  | case | NBEAL1 |
| 2 | 204103815 | G | C | novel |  | Yes |  | case | CYP20A1 |
| 2 | 204103851 | T | C | novel |  | Yes |  | case | CYP20A1 |
| 2 | 204110578 | G | C | novel |  |  |  | case | CYP20A1 |
| 2 | 204110618 | G | A | novel | Yes |  |  | case | CYP20A1 |
| 2 | 204111498 | C | T | novel |  | Yes |  | case | CYP20A1 |
| 2 | 204111524 | C | T | novel |  |  |  | case | CYP20A1 |
| 2 | 204111542 | A | G | novel |  |  |  | both | CYP20A1 |
| 2 | 204111581 | T | C | rs150778410 |  |  |  | control | CYP20A1 |
| 2 | 204111582 | A | G | novel |  |  |  | case | CYP20A1 |
| 2 | 204111584 | T | C | novel |  |  |  | both | CYP20A1 |
| 2 | 204111587 | A | G | rs149546776 |  | Yes |  | case | CYP20A1 |
| 2 | 204111603 | A | C | rs182035689 | Yes |  |  | both | CYP20A1 |
| 2 | 204116690 | C | T | rs2043449 | Yes |  |  | both | CYP20A1 |
| 2 | 204116730 | A | T | rs201173639 |  | Yes | Yes | both | CYP20A1 |
| 2 | 204116735 | T | C | novel |  |  |  | control | CYP20A1 |
| 2 | 204116760 | A | C | novel | Yes |  |  | case | CYP20A1 |
| 2 | 204116764 | G | A | rs200681711 | Yes |  |  | both | CYP20A1 |
| 2 | 204116787 | C | T | novel |  | Yes |  | case | CYP20A1 |
| 2 | 204131251 | G | A | rs376437549 |  | Yes |  | control | CYP20A1 |
| 2 | 204131261 | C | T | novel | Yes | Yes |  | case | CYP20A1 |
| 2 | 204131274 | G | C | novel |  |  |  | control | CYP20A1 |
| 2 | 204131287 | T | C | novel |  | Yes |  | both | CYP20A1 |
| 2 | 204131321 | G | A | rs199882728 | Yes |  |  | both | CYP20A1 |
| 2 | 204131355 | T | C | novel | Yes |  |  | case | CYP20A1 |
| 2 | 204131371 | C | G | novel | Yes |  |  | both | CYP20A1 |
| 2 | 204131381 | T | C | novel |  |  |  | control | CYP20A1 |
| 2 | 204131388 | G | A | novel | Yes |  |  | both | CYP20A1 |
| 2 | 204143326 | G | A | novel |  |  |  | case | CYP20A1 |
| 2 | 204143380 | A | C | novel |  |  |  | control | CYP20A1 |
| 2 | 204143402 | C | T | novel |  | Yes |  | both | CYP20A1 |
| 2 | 204143413 | G | T | novel |  | Yes | Yes | control | CYP20A1 |
| 2 | 204150427 | A | G | novel |  |  |  | both | CYP20A1 |
| 2 | 204150433 | T | C | novel |  |  |  | control | CYP20A1 |
| 2 | 204154493 | A | G | novel |  |  |  | case | CYP20A1 |
| 2 | 204154508 | A | G | novel | Yes |  |  | control | CYP20A1 |
| 2 | 204154519 | G | C | novel |  | Yes |  | case | CYP20A1 |
| 2 | 204154552 | T | C | rs1048013 | Yes |  |  | both | CYP20A1 |
| 2 | 204157009 | A | G | novel |  | Yes |  | control | CYP20A1 |
| 2 | 204161214 | C | T | novel |  | Yes |  | control | CYP20A1 |
| 2 | 204161238 | C | CT | novel |  | Yes | Yes | case | CYP20A1 |
| 2 | 204161248 | C | A | novel |  | Yes |  | case | CYP20A1 |
| 2 | 204161284 | A | G | novel |  | Yes |  | both | CYP20A1 |
| 2 | 204161503 | A | G | novel | Yes |  |  | both | CYP20A1 |
| 2 | 204161513 | T | G | novel | Yes |  |  | both | CYP20A1 |
| 2 | 204161533 | A | C | novel |  |  |  | control | CYP20A1 |
| 4 | 156696147 | G | A | rs75622942 |  | Yes |  | both | GUCY1B3 |
| 4 | 156710883 | T | C | rs367583649 |  | Yes |  | control | GUCY1B3 |
| 4 | 156710978 | T | C | novel | Yes | Yes |  | control | GUCY1B3 |
| 4 | 156715015 | G | A | novel |  |  |  | case | GUCY1B3 |
| 4 | 156715021 | T | G | novel |  |  |  | control | GUCY1B3 |
| 4 | 156715042 | T | C | novel | Yes |  |  | case | GUCY1B3 |
| 4 | 156715054 | C | T | novel |  | Yes |  | case | GUCY1B3 |
| 4 | 156715092 | A | G | novel |  |  |  | case | GUCY1B3 |
| 4 | 156715112 | T | A | novel | Yes |  |  | both | GUCY1B3 |
| 4 | 156715136 | T | C | novel |  | Yes |  | both | GUCY1B3 |
| 4 | 156715143 | C | T | novel |  |  |  | case | GUCY1B3 |
| 4 | 156715173 | G | A | rs140646673 | Yes |  |  | both | GUCY1B3 |
| 4 | 156716519 | C | G | novel |  | Yes |  | case | GUCY1B3 |
| 4 | 156716531 | T | G | rs193112561 |  | Yes |  | both | GUCY1B3 |
| 4 | 156716599 | C | T | novel | Yes | Yes |  | case | GUCY1B3 |
| 4 | 156717588 | G | C | novel | Yes |  |  | case | GUCY1B3 |
| 4 | 156717651 | G | A | novel |  | Yes |  | control | GUCY1B3 |
| 4 | 156721054 | C | A | novel |  | Yes |  | both | GUCY1B3 |
| 4 | 156721098 | A | G | rs17854507 |  | Yes |  | both | GUCY1B3 |
| 4 | 156721101 | T | C | rs201839794 |  | Yes |  | both | GUCY1B3 |
| 4 | 156721119 | G | A | novel |  | Yes |  | control | GUCY1B3 |
| 4 | 156721152 | G | A | novel |  | Yes |  | both | GUCY1B3 |
| 4 | 156721169 | A | C | novel |  |  |  | control | GUCY1B3 |
| 4 | 156721198 | T | C | rs2229202 |  | Yes |  | both | GUCY1B3 |
| 4 | 156721226 | T | C | rs2229203 |  |  |  | both | GUCY1B3 |
| 4 | 156723518 | A | G | novel |  | Yes |  | both | GUCY1B3 |
| 4 | 156723546 | T | C | novel |  | Yes |  | control | GUCY1B3 |
| 4 | 156723551 | G | A | novel |  | Yes |  | case | GUCY1B3 |
| 4 | 156723630 | A | G | novel |  |  |  | case | GUCY1B3 |
| 4 | 156723656 | T | C | rs146343051 |  | Yes |  | both | GUCY1B3 |
| 4 | 156723671 | T | C | rs201458783 |  | Yes |  | case | GUCY1B3 |
| 4 | 156723683 | T | C | rs139612573 |  | Yes |  | both | GUCY1B3 |
| 4 | 156723686 | G | A | novel |  | Yes |  | control | GUCY1B3 |
| 4 | 156723721 | C | T | novel |  |  |  | control | GUCY1B3 |
| 4 | 156724781 | A | G | novel |  | Yes |  | both | GUCY1B3 |
| 4 | 156724855 | T | A | novel |  |  |  | control | GUCY1B3 |
| 4 | 156724865 | A | G | novel |  | Yes |  | both | GUCY1B3 |
| 4 | 156724909 | G | C | novel | Yes |  |  | case | GUCY1B3 |
| 4 | 156725787 | G | A | novel |  |  |  | control | GUCY1B3 |
| 4 | 156725797 | A | G | rs371379330 |  |  |  | case | GUCY1B3 |
| 4 | 156725822 | C | G | novel |  | Yes |  | control | GUCY1B3 |
| 4 | 156725893 | A | C | novel |  | Yes |  | case | GUCY1B3 |
| 4 | 156725894 | T | A | novel |  | Yes |  | case | GUCY1B3 |
| 4 | 156726321 | A | G | novel |  |  |  | case | GUCY1B3 |
| 4 | 156726329 | G | A | novel |  |  |  | both | GUCY1B3 |
| 4 | 156726357 | G | A | novel |  | Yes |  | case | GUCY1B3 |
| 4 | 156726363 | G | A | novel |  | Yes |  | case | GUCY1B3 |
| 6 | 161127480 | T | G | novel |  |  |  | control | PLG |
| 6 | 161127492 | G | A | novel | Yes |  |  | both | PLG |
| 6 | 161127495 | A | G | novel | Yes |  |  | control | PLG |
| 6 | 161127507 | A | C | novel |  |  |  | case | PLG |
| 6 | 161127508 | T | A | novel |  |  |  | case | PLG |
| 6 | 161127520 | A | G | novel |  |  |  | case | PLG |
| 6 | 161127528 | C | G | novel |  |  |  | both | PLG |
| 6 | 161127549 | A | G | novel | Yes |  |  | both | PLG |
| 6 | 161127557 | T | C | rs144100362 |  | Yes |  | control | PLG |
| 6 | 161128803 | G | T | novel |  |  |  | case | PLG |
| 6 | 161132117 | G | T | novel |  |  |  | control | PLG |
| 6 | 161132139 | T | G | novel | Yes |  |  | both | PLG |
| 6 | 161132157 | T | C | rs139357983 | Yes |  |  | control | PLG |
| 6 | 161132181 | A | T | novel |  |  |  | case | PLG |
| 6 | 161132200 | C | T | novel |  | Yes |  | control | PLG |
| 6 | 161132209 | C | T | novel |  | Yes |  | case | PLG |
| 6 | 161134036 | A | C | novel |  |  |  | case | PLG |
| 6 | 161134048 | C | A | novel |  | Yes |  | control | PLG |
| 6 | 161134079 | T | A | novel |  |  |  | control | PLG |
| 6 | 161134081 | T | C | rs372617319 |  | Yes |  | case | PLG |
| 6 | 161134086 | T | C | rs201792453 | Yes |  |  | both | PLG |
| 6 | 161134106 | G | A | novel |  |  |  | case | PLG |
| 6 | 161134111 | C | T | novel |  | Yes |  | control | PLG |
| 6 | 161134138 | T | C | novel |  | Yes |  | case | PLG |
| 6 | 161135835 | C | T | novel |  |  |  | control | PLG |
| 6 | 161135859 | T | A | rs150072546 | Yes |  |  | both | PLG |
| 6 | 161135860 | T | C | rs145192723 |  | Yes |  | both | PLG |
| 6 | 161135900 | T | G | rs201014517 |  |  |  | control | PLG |
| 6 | 161135924 | A | G | rs374234922 |  |  |  | case | PLG |
| 6 | 161135933 | C | T | rs151092364 |  |  |  | control | PLG |
| 6 | 161137712 | A | G | rs121918030 |  |  |  | case | PLG |
| 6 | 161137719 | T | C | rs200226472 |  | Yes |  | both | PLG |
| 6 | 161137752 | T | C | novel |  | Yes |  | case | PLG |
| 6 | 161137765 | T | C | novel | Yes |  |  | case | PLG |
| 6 | 161137779 | C | T | rs14224 |  | Yes |  | both | PLG |
| 6 | 161139357 | T | C | novel |  | Yes |  | case | PLG |
| 6 | 161139390 | T | C | novel |  | Yes |  | both | PLG |
| 6 | 161139408 | G | C | novel |  | Yes |  | control | PLG |
| 6 | 161139438 | C | T | novel |  | Yes |  | case | PLG |
| 6 | 161139462 | A | C | novel |  |  |  | control | PLG |
| 6 | 161139480 | T | C | rs1130656 |  | Yes |  | both | PLG |
| 6 | 161139732 | C | G | novel | Yes |  |  | both | PLG |
| 6 | 161139758 | T | C | novel |  | Yes |  | control | PLG |
| 6 | 161139759 | A | G | novel |  |  |  | control | PLG |
| 6 | 161139765 | A | AG | novel |  | Yes | Yes | case | PLG |
| 6 | 161139798 | A | G | rs199771790 | Yes |  |  | both | PLG |
| 6 | 161139802 | A | G | novel | Yes |  |  | both | PLG |
| 6 | 161139810 | G | T | novel |  |  |  | both | PLG |
| 6 | 161143443 | T | C | novel |  |  |  | case | PLG |
| 6 | 161143463 | T | G | rs121918028 | Yes |  |  | both | PLG |
| 6 | 161143466 | T | C | novel |  | Yes | Yes | control | PLG |
| 6 | 161143500 | A | G | novel | Yes |  |  | both | PLG |
| 6 | 161143519 | T | C | novel |  | Yes |  | both | PLG |
| 6 | 161143566 | A | G | novel |  |  |  | case | PLG |
| 6 | 161143573 | A | G | novel |  | Yes |  | case | PLG |
| 6 | 161143582 | T | A | novel |  | Yes |  | control | PLG |
| 6 | 161143599 | T | C | novel |  |  |  | control | PLG |
| 6 | 161152104 | T | C | novel |  | Yes |  | case | PLG |
| 6 | 161152117 | T | G | novel |  |  |  | case | PLG |
| 6 | 161152119 | T | C | novel |  | Yes |  | control | PLG |
| 6 | 161152156 | A | G | novel | Yes |  |  | both | PLG |
| 6 | 161152197 | G | A | novel |  | Yes |  | control | PLG |
| 6 | 161152229 | T | TCC | novel |  | Yes | Yes | control | PLG |
| 6 | 161152233 | G | GC | novel |  | Yes | Yes | control | PLG |
| 6 | 161152258 | A | G | novel |  |  |  | case | PLG |
| 6 | 161152260 | G | A | novel |  | Yes |  | both | PLG |
| 6 | 161152819 | T | C | rs4252128 | Yes |  |  | both | PLG |
| 6 | 161152837 | T | C | rs140970354 | Yes |  |  | both | PLG |
| 6 | 161152842 | C | T | novel |  |  |  | case | PLG |
| 6 | 161152897 | G | C | novel | Yes |  |  | both | PLG |
| 6 | 161152908 | A | G | novel |  |  |  | control | PLG |
| 6 | 161155024 | T | C | rs193059713 |  | Yes |  | both | PLG |
| 6 | 161155106 | T | A | novel |  |  |  | case | PLG |
| 6 | 161157920 | A | G | novel |  | Yes |  | both | PLG |
| 6 | 161157929 | C | A | novel |  | Yes |  | case | PLG |
| 6 | 161157968 | C | T | novel |  | Yes |  | case | PLG |
| 6 | 161158031 | G | T | novel |  | Yes |  | both | PLG |
| 6 | 161159625 | A | G | rs121918027 | Yes |  |  | both | PLG |
| 6 | 161159626 | T | C | novel | Yes |  |  | control | PLG |
| 6 | 161159627 | C | T | novel |  | Yes |  | both | PLG |
| 6 | 161160107 | G | A | novel |  |  |  | control | PLG |
| 6 | 161160165 | T | A | novel |  |  |  | both | PLG |
| 6 | 161162350 | A | G | rs142551860 |  |  |  | case | PLG |
| 6 | 161162386 | C | T | novel |  |  |  | control | PLG |
| 6 | 161162392 | G | A | novel | Yes |  |  | both | PLG |
| 6 | 161162404 | A | G | novel | Yes |  |  | both | PLG |
| 6 | 161162406 | C | T | rs4252170 |  | Yes |  | both | PLG |
| 6 | 161162407 | A | G | rs181030365 | Yes |  |  | both | PLG |
| 6 | 161162411 | A | G | rs147930532 |  |  |  | case | PLG |
| 6 | 161162415 | T | C | novel |  | Yes |  | both | PLG |
| 6 | 161173158 | A | G | novel |  |  |  | case | PLG |
| 6 | 161173172 | A | G | novel |  | Yes |  | both | PLG |
| 6 | 161173198 | G | A | novel |  |  |  | control | PLG |
| 6 | 161173212 | T | C | novel |  |  |  | both | PLG |
| 6 | 161173222 | A | T | novel |  |  |  | control | PLG |
| 6 | 161173229 | C | T | novel |  | Yes |  | case | PLG |
| 6 | 161173248 | A | G | novel |  |  |  | control | PLG |
| 6 | 161173272 | A | G | rs121918033 | Yes |  |  | both | PLG |
| 9 | 21968234 | A | G | novel |  | Yes |  | both | CDKN2A |
| 9 | 21968238 | T | A | novel | Yes |  |  | both | CDKN2A |
| 9 | 21968727 | T | C | rs189127161 |  | Yes |  | both | CDKN2A |
| 9 | 21968732 | A | G | rs181044510 | Yes |  |  | both | CDKN2A |
| 9 | 21968753 | G | A | novel |  | Yes |  | both | CDKN2A |
| 9 | 21968762 | T | C | novel |  |  |  | control | CDKN2A |
| 9 | 21970916 | T | C | rs3731249 |  |  |  | case | CDKN2A |
| 9 | 21970930 | C | G | novel |  |  |  | both | CDKN2A |
| 9 | 21970942 | T | C | rs149937815 | Yes |  |  | control | CDKN2A |
| 9 | 21970953 | T | C | novel |  | Yes |  | both | CDKN2A |
| 9 | 21971014 | T | A | novel |  |  |  | control | CDKN2A |
| 9 | 21971043 | T | G | novel | Yes |  |  | both | CDKN2A |
| 9 | 21971053 | T | G | rs137854598 |  |  |  | control | CDKN2A |
| 9 | 21971120 | T | G | novel |  |  |  | control | CDKN2A |
| 9 | 21971122 | A | G | novel | Yes |  |  | both | CDKN2A |
| 9 | 21971133 | A | G | novel |  |  |  | case | CDKN2A |
| 9 | 21971161 | C | T | novel |  |  |  | both | CDKN2A |
| 9 | 21971179 | T | G | novel |  |  |  | case | CDKN2A |
| 9 | 21971183 | A | C | novel |  |  |  | control | CDKN2A |
| 9 | 21974705 | T | G | rs373407950 |  |  |  | control | CDKN2A |
| 9 | 21974708 | A | G | novel |  |  |  | case | CDKN2A |
| 9 | 21974805 | G | T | novel |  |  |  | control | CDKN2A |
| 9 | 21994160 | T | C | novel |  | Yes |  | both | CDKN2A |
| 9 | 21994218 | A | C | novel |  |  |  | both | CDKN2A |
| 9 | 21994228 | T | C | novel |  |  |  | case | CDKN2A |
| 9 | 21994253 | A | G | novel |  | Yes |  | both | CDKN2A |
| 9 | 21994254 | A | T | novel |  |  |  | control | CDKN2A |
| 9 | 21994278 | A | G | novel |  |  |  | case | CDKN2A |
| 9 | 21994281 | A | G | novel |  |  |  | case | CDKN2A |
| 9 | 21994323 | T | C | novel |  |  |  | both | CDKN2A |
| 11 | 103780426 | T | C | novel | Yes | Yes |  | case | PDGFD |
| 11 | 103780455 | G | A | rs10791649 |  | Yes |  | both | PDGFD |
| 11 | 103780457 | G | A | novel |  | Yes |  | case | PDGFD |
| 11 | 103780459 | T | C | rs144227909 |  |  |  | control | PDGFD |
| 11 | 103780479 | C | G | novel |  | Yes |  | case | PDGFD |
| 11 | 103780502 | C | T | novel | Yes |  |  | control | PDGFD |
| 11 | 103780504 | C | G | novel |  |  |  | control | PDGFD |
| 11 | 103780541 | C | G | novel |  |  |  | control | PDGFD |
| 11 | 103780547 | A | C | novel |  | Yes |  | case | PDGFD |
| 11 | 103797733 | A | G | novel |  | Yes |  | case | PDGFD |
| 11 | 103797807 | A | G | novel | Yes | Yes |  | both | PDGFD |
| 11 | 103797821 | T | C | rs202233663 |  | Yes |  | control | PDGFD |
| 11 | 103797854 | G | A | rs145779686 |  |  |  | control | PDGFD |
| 11 | 103814191 | T | C | rs147536650 | Yes |  |  | both | PDGFD |
| 11 | 103814203 | A | G | novel | Yes |  |  | both | PDGFD |
| 11 | 103814204 | G | A | novel | Yes |  |  | both | PDGFD |
| 11 | 103814218 | T | C | novel | Yes |  |  | both | PDGFD |
| 11 | 103814219 | A | G | novel |  |  |  | case | PDGFD |
| 11 | 103814332 | A | G | rs141538106 |  |  |  | both | PDGFD |
| 11 | 103814350 | A | G | novel | Yes |  |  | case | PDGFD |
| 11 | 103814371 | C | G | novel |  |  |  | case | PDGFD |
| 11 | 103814374 | C | A | novel | Yes |  |  | both | PDGFD |
| 11 | 103814378 | T | C | novel |  | Yes |  | control | PDGFD |
| 11 | 103818395 | C | T | rs35045740 | Yes |  |  | both | PDGFD |
| 11 | 103866844 | A | G | rs149741224 |  | Yes |  | case | PDGFD |
| 11 | 103866872 | A | G | rs201226151 |  |  |  | control | PDGFD |
| 11 | 103866873 | A | T | novel |  |  |  | both | PDGFD |
| 11 | 103866925 | G | A | novel |  | Yes |  | case | PDGFD |
| 11 | 103866942 | T | C | novel | Yes |  |  | control | PDGFD |
| 11 | 103866972 | G | A | novel | Yes | Yes |  | both | PDGFD |
| 11 | 103870838 | G | C | novel |  | Yes |  | control | PDGFD |
| 11 | 103870840 | A | G | novel | Yes |  |  | case | PDGFD |
| 11 | 103870844 | G | A | novel |  | Yes |  | control | PDGFD |
| 11 | 103870898 | T | C | novel |  | Yes |  | control | PDGFD |
| 11 | 103870922 | A | G | novel |  | Yes |  | both | PDGFD |
| 11 | 104034567 | G | A | novel | Yes |  |  | both | PDGFD |
| 11 | 104034632 | G | GTAGA | novel |  | Yes | Yes | control | PDGFD |
| 11 | 104034635 | T | G | novel |  | Yes |  | case | PDGFD |
| 12 | 111052001 | A | G | novel | Yes |  |  | case | TCTN1 |
| 12 | 111052067 | T | C | novel |  |  |  | both | TCTN1 |
| 12 | 111052071 | A | G | novel |  | Yes |  | case | TCTN1 |
| 12 | 111052123 | A | G | novel |  |  |  | case | TCTN1 |
| 12 | 111052144 | T | C | novel |  |  |  | case | TCTN1 |
| 12 | 111057655 | T | G | novel |  |  |  | control | TCTN1 |
| 12 | 111057718 | A | G | rs145478892 |  |  |  | control | TCTN1 |
| 12 | 111057747 | G | A | rs140230455 |  | Yes |  | both | TCTN1 |
| 12 | 111057762 | GT | G | novel |  | Yes | Yes | case | TCTN1 |
| 12 | 111064168 | A | G | novel | Yes |  |  | both | TCTN1 |
| 12 | 111064170 | T | C | novel |  | Yes |  | control | TCTN1 |
| 12 | 111064171 | A | G | novel | Yes |  |  | case | TCTN1 |
| 12 | 111064184 | A | G | novel | Yes |  |  | case | TCTN1 |
| 12 | 111064229 | T | C | novel |  |  |  | both | TCTN1 |
| 12 | 111066587 | A | C | rs117896500 | Yes |  |  | both | TCTN1 |
| 12 | 111066692 | G | A | novel |  |  |  | case | TCTN1 |
| 12 | 111070296 | A | C | novel |  |  |  | case | TCTN1 |
| 12 | 111072203 | T | C | novel |  | Yes | Yes | case | TCTN1 |
| 12 | 111072207 | T | C | novel |  |  |  | control | TCTN1 |
| 12 | 111072224 | T | G | novel | Yes |  |  | both | TCTN1 |
| 12 | 111072238 | G | C | novel |  | Yes |  | case | TCTN1 |
| 12 | 111072247 | T | C | novel |  | Yes |  | control | TCTN1 |
| 12 | 111072476 | A | G | rs200574241 |  | Yes |  | control | TCTN1 |
| 12 | 111072530 | G | T | novel | Yes | Yes |  | control | TCTN1 |
| 12 | 111072568 | C | G | novel |  |  |  | control | TCTN1 |
| 12 | 111072571 | T | C | rs199525130 |  |  |  | control | TCTN1 |
| 12 | 111072572 | A | G | novel |  | Yes |  | case | TCTN1 |
| 12 | 111072581 | C | G | novel |  | Yes |  | case | TCTN1 |
| 12 | 111072587 | G | A | novel |  | Yes |  | case | TCTN1 |
| 12 | 111078211 | T | C | novel |  | Yes |  | case | TCTN1 |
| 12 | 111078224 | T | C | rs371899538 |  | Yes |  | both | TCTN1 |
| 12 | 111078270 | T | C | novel |  |  |  | control | TCTN1 |
| 12 | 111078277 | T | C | novel |  | Yes |  | both | TCTN1 |
| 12 | 111078292 | T | C | novel |  | Yes |  | control | TCTN1 |
| 12 | 111078304 | T | C | rs145970332 |  | Yes |  | case | TCTN1 |
| 12 | 111078837 | G | C | novel |  | Yes | Yes | case | TCTN1 |
| 12 | 111078888 | T | C | rs377300877 |  | Yes |  | case | TCTN1 |
| 12 | 111078889 | T | G | novel |  |  |  | control | TCTN1 |
| 12 | 111078905 | T | G | novel | Yes |  |  | control | TCTN1 |
| 12 | 111078910 | A | G | rs374701683 |  |  |  | control | TCTN1 |
| 12 | 111079350 | G | A | novel |  |  |  | case | TCTN1 |
| 12 | 111079360 | T | C | novel |  |  |  | control | TCTN1 |
| 12 | 111079391 | T | C | novel |  | Yes |  | both | TCTN1 |
| 12 | 111079393 | C | T | novel |  |  |  | control | TCTN1 |
| 12 | 111079402 | T | C | novel |  |  |  | control | TCTN1 |
| 12 | 111079430 | T | G | novel |  |  |  | case | TCTN1 |
| 12 | 111080076 | G | A | novel |  |  |  | control | TCTN1 |
| 12 | 111080093 | G | T | novel |  | Yes |  | control | TCTN1 |
| 12 | 111080134 | C | T | novel | Yes |  |  | both | TCTN1 |
| 12 | 111080142 | T | G | novel |  |  |  | control | TCTN1 |
| 12 | 111080150 | T | C | novel |  | Yes |  | both | TCTN1 |
| 12 | 111082789 | T | C | novel |  |  |  | case | TCTN1 |
| 12 | 111082807 | G | A | novel |  |  |  | control | TCTN1 |
| 12 | 111082827 | C | T | rs114568905 |  |  |  | control | TCTN1 |
| 12 | 111082836 | T | G | rs118096349 | Yes |  |  | both | TCTN1 |
| 12 | 111082860 | G | T | novel |  |  |  | control | TCTN1 |
| 12 | 111082877 | T | C | novel |  | Yes |  | both | TCTN1 |
| 12 | 111082879 | G | A | novel |  |  |  | case | TCTN1 |
| 12 | 111082922 | G | A | novel |  | Yes |  | both | TCTN1 |
| 12 | 111085027 | A | G | novel |  | Yes |  | control | TCTN1 |
| 12 | 111085069 | T | C | rs368907353 |  | Yes |  | case | TCTN1 |
| 12 | 111085075 | T | C | novel |  | Yes |  | case | TCTN1 |
| 12 | 111085594 | T | C | rs373723058 |  |  |  | control | TCTN1 |
| 12 | 111085674 | T | A | novel |  |  |  | case | TCTN1 |
| 12 | 111085675 | T | A | novel |  |  |  | case | TCTN1 |
| 12 | 111085677 | A | T | novel | Yes |  |  | control | TCTN1 |
| 12 | 111085678 | A | T | novel | Yes |  |  | control | TCTN1 |
| 12 | 111085698 | C | CGTAA | novel |  | Yes | Yes | case | TCTN1 |
| 17 | 1964815 | A | C | novel |  |  |  | control | SMG6 |
| 17 | 1964849 | T | C | novel |  | Yes |  | case | SMG6 |
| 17 | 1964859 | T | C | rs369323853 | Yes | Yes |  | both | SMG6 |
| 17 | 1964876 | T | C | rs144129895 |  | Yes |  | both | SMG6 |
| 17 | 1964895 | T | C | novel |  | Yes |  | control | SMG6 |
| 17 | 1964898 | T | A | novel |  |  |  | control | SMG6 |
| 17 | 1968830 | T | C | novel |  | Yes |  | control | SMG6 |
| 17 | 1968907 | T | C | novel |  | Yes |  | case | SMG6 |
| 17 | 1968967 | C | T | rs2232487 | Yes |  |  | case | SMG6 |
| 17 | 1968971 | C | T | novel |  |  |  | control | SMG6 |
| 17 | 1972082 | A | C | novel |  | Yes |  | both | SMG6 |
| 17 | 1972116 | T | C | rs2232483 | Yes |  |  | case | SMG6 |
| 17 | 1972147 | T | C | rs139385089 |  |  |  | control | SMG6 |
| 17 | 1972163 | C | G | novel |  | Yes |  | case | SMG6 |
| 17 | 1972209 | C | T | rs2273980 | Yes |  |  | both | SMG6 |
| 17 | 1985110 | A | C | novel |  |  |  | case | SMG6 |
| 17 | 1985205 | T | C | rs376160722 | Yes |  |  | both | SMG6 |
| 17 | 1989027 | G | C | rs187319098 | Yes |  |  | both | SMG6 |
| 17 | 1989054 | C | T | novel |  |  |  | control | SMG6 |
| 17 | 1989081 | A | G | novel |  |  |  | control | SMG6 |
| 17 | 1989098 | G | T | novel | Yes |  |  | case | SMG6 |
| 17 | 1989104 | G | C | novel |  |  |  | case | SMG6 |
| 17 | 1989120 | A | G | novel |  | Yes |  | control | SMG6 |
| 17 | 2075972 | T | C | novel | Yes |  |  | both | SMG6 |
| 17 | 2075982 | C | G | novel |  |  |  | case | SMG6 |
| 17 | 2076025 | T | C | novel | Yes |  |  | case | SMG6 |
| 17 | 2076039 | C | G | novel |  |  |  | control | SMG6 |
| 17 | 2076071 | G | T | novel |  |  |  | control | SMG6 |
| 17 | 2076084 | T | C | rs191617249 |  | Yes |  | both | SMG6 |
| 17 | 2076129 | T | C | novel |  | Yes |  | case | SMG6 |
| 17 | 2076132 | T | C | novel |  | Yes |  | case | SMG6 |
| 17 | 2076138 | C | T | novel |  | Yes |  | both | SMG6 |
| 17 | 2076140 | A | C | novel |  |  |  | control | SMG6 |
| 17 | 2076141 | G | A | novel |  | Yes |  | both | SMG6 |
| 17 | 2089972 | G | A | novel | Yes |  |  | case | SMG6 |
| 17 | 2090032 | G | T | novel |  | Yes |  | control | SMG6 |
| 17 | 2091699 | T | C | novel | Yes |  |  | case | SMG6 |
| 17 | 2091721 | A | G | rs200765278 |  | Yes |  | both | SMG6 |
| 17 | 2091755 | A | C | novel |  | Yes |  | control | SMG6 |
| 17 | 2091765 | T | C | rs903160 | Yes |  |  | both | SMG6 |
| 17 | 2091799 | T | C | rs142201292 |  | Yes |  | case | SMG6 |
| 17 | 2139842 | T | C | rs368650142 |  |  |  | case | SMG6 |
| 17 | 2139843 | A | G | rs376512734 | Yes | Yes |  | case | SMG6 |
| 17 | 2139845 | T | G | novel |  |  |  | case | SMG6 |
| 17 | 2139855 | C | T | novel | Yes |  |  | case | SMG6 |
| 17 | 2139858 | A | G | novel |  |  |  | control | SMG6 |
| 17 | 2139886 | T | G | novel |  |  |  | control | SMG6 |
| 17 | 2139898 | A | G | novel |  | Yes |  | control | SMG6 |
| 17 | 2139901 | A | C | novel |  |  |  | case | SMG6 |
| 17 | 2147959 | A | G | novel |  | Yes |  | both | SMG6 |
| 17 | 2147982 | C | T | novel | Yes |  |  | both | SMG6 |
| 17 | 2147998 | C | T | rs148043002 | Yes |  |  | both | SMG6 |
| 17 | 2185956 | T | G | rs191133452 | Yes |  |  | both | SMG6 |
| 17 | 2185960 | T | C | novel |  |  |  | case | SMG6 |
| 17 | 2185962 | A | C | novel |  | Yes |  | case | SMG6 |
| 17 | 2186017 | A | G | novel | Yes |  |  | case | SMG6 |
| 17 | 2186018 | C | G | novel |  |  |  | both | SMG6 |
| 17 | 2186085 | T | C | novel |  | Yes |  | case | SMG6 |
| 17 | 2186087 | C | T | novel |  |  |  | control | SMG6 |
| 17 | 2186100 | T | C | rs749240 |  | Yes |  | both | SMG6 |
| 17 | 2186125 | A | T | rs142250229 | Yes |  |  | both | SMG6 |
| 17 | 2186947 | G | A | rs372239404 | Yes | Yes |  | both | SMG6 |
| 17 | 2186980 | G | C | novel |  |  |  | control | SMG6 |
| 17 | 2186992 | C | CTG | novel |  | Yes | Yes | control | SMG6 |
| 17 | 2187007 | C | T | novel |  | Yes |  | case | SMG6 |
| 17 | 2187015 | A | G | rs142112792 |  | Yes |  | both | SMG6 |
| 17 | 2195847 | A | G | novel |  | Yes |  | case | SMG6 |
| 17 | 2196208 | A | G | novel |  | Yes |  | case | SMG6 |
| 17 | 2200545 | A | G | novel |  |  |  | case | SMG6 |
| 17 | 2200560 | A | G | rs370989363 |  | Yes |  | both | SMG6 |
| 17 | 2201177 | T | G | novel | Yes | Yes |  | control | SMG6 |
| 17 | 2201186 | A | G | novel |  | Yes |  | case | SMG6 |
| 17 | 2201198 | A | G | novel |  |  |  | control | SMG6 |
| 17 | 2201207 | A | C | novel |  |  |  | control | SMG6 |
| 17 | 2201220 | T | G | novel |  | Yes |  | both | SMG6 |
| 17 | 2201270 | A | G | novel |  | Yes |  | control | SMG6 |
| 17 | 2201284 | C | T | novel |  | Yes |  | control | SMG6 |
| 17 | 2201296 | A | G | novel |  | Yes |  | case | SMG6 |
| 17 | 2201310 | C | T | novel |  | Yes |  | case | SMG6 |
| 17 | 2201318 | C | T | novel |  |  |  | both | SMG6 |
| 17 | 2201323 | T | C | rs138966738 |  |  |  | case | SMG6 |
| 17 | 2202209 | A | G | novel |  |  |  | control | SMG6 |
| 17 | 2202211 | A | C | novel |  |  |  | control | SMG6 |
| 17 | 2202232 | G | A | novel |  | Yes |  | case | SMG6 |
| 17 | 2202261 | C | T | novel |  |  |  | case | SMG6 |
| 17 | 2202344 | G | T | novel |  |  |  | case | SMG6 |
| 17 | 2202357 | C | T | rs201331245 |  |  |  | case | SMG6 |
| 17 | 2202397 | T | C | novel |  | Yes |  | both | SMG6 |
| 17 | 2202398 | A | G | novel |  |  |  | case | SMG6 |
| 17 | 2202447 | A | G | novel |  |  |  | case | SMG6 |
| 17 | 2202479 | A | G | rs140068523 |  |  |  | case | SMG6 |
| 17 | 2202485 | C | G | novel | Yes | Yes |  | both | SMG6 |
| 17 | 2202512 | T | C | novel | Yes |  |  | control | SMG6 |
| 17 | 2202513 | C | G | novel |  |  |  | case | SMG6 |
| 17 | 2202514 | C | G | novel |  | Yes |  | both | SMG6 |
| 17 | 2202590 | C | G | novel |  |  |  | control | SMG6 |
| 17 | 2202625 | C | T | rs369510433 |  | Yes |  | control | SMG6 |
| 17 | 2202635 | A | G | rs145169086 | Yes |  |  | both | SMG6 |
| 17 | 2202653 | A | G | novel |  |  |  | control | SMG6 |
| 17 | 2202668 | C | T | novel |  |  |  | case | SMG6 |
| 17 | 2202686 | T | C | novel | Yes |  |  | both | SMG6 |
| 17 | 2202705 | A | G | novel |  |  |  | case | SMG6 |
| 17 | 2202729 | C | T | novel |  |  |  | case | SMG6 |
| 17 | 2202749 | T | C | rs200099320 |  |  |  | case | SMG6 |
| 17 | 2202766 | A | G | rs139116937 |  | Yes |  | both | SMG6 |
| 17 | 2202830 | T | C | novel |  |  |  | case | SMG6 |
| 17 | 2202850 | C | T | novel |  | Yes |  | both | SMG6 |
| 17 | 2202882 | G | A | novel | Yes |  |  | both | SMG6 |
| 17 | 2202887 | C | T | novel |  |  |  | both | SMG6 |
| 17 | 2202890 | T | C | novel |  |  |  | both | SMG6 |
| 17 | 2202898 | A | G | novel |  | Yes |  | case | SMG6 |
| 17 | 2202914 | A | G | novel |  |  |  | control | SMG6 |
| 17 | 2202919 | C | T | novel |  | Yes |  | case | SMG6 |
| 17 | 2202926 | A | T | novel |  |  |  | both | SMG6 |
| 17 | 2202943 | T | C | rs216196 |  | Yes |  | both | SMG6 |
| 17 | 2202995 | T | C | rs140472144 |  |  |  | control | SMG6 |
| 17 | 2203025 | G | T | rs1885987 | Yes |  |  | both | SMG6 |
| 17 | 2203046 | T | C | novel | Yes |  |  | both | SMG6 |
| 17 | 2203048 | A | G | novel |  | Yes |  | case | SMG6 |
| 17 | 2203057 | C | G | rs200020527 | Yes |  |  | both | SMG6 |
| 17 | 2203071 | T | G | rs149069908 | Yes |  |  | both | SMG6 |
| 17 | 2203146 | A | T | novel |  |  |  | case | SMG6 |
| 17 | 2203167 | T | G | rs216195 | Yes |  |  | both | SMG6 |
| 17 | 2203175 | G | C | rs1885986 | Yes |  |  | both | SMG6 |
| 17 | 2203197 | G | C | novel |  |  |  | case | SMG6 |
| 17 | 2203206 | A | G | novel | Yes |  |  | both | SMG6 |
| 17 | 2203208 | T | C | novel |  |  |  | case | SMG6 |
| 17 | 2203215 | G | A | novel |  |  |  | control | SMG6 |
| 17 | 2203226 | A | G | novel | Yes |  |  | case | SMG6 |
| 17 | 2203235 | C | G | novel |  |  |  | case | SMG6 |
| 17 | 2203252 | A | G | rs201836751 |  | Yes |  | both | SMG6 |
| 17 | 2203267 | A | G | novel |  | Yes |  | case | SMG6 |
| 17 | 2203290 | A | G | rs147212426 |  |  |  | case | SMG6 |
| 17 | 2203294 | T | C | novel |  | Yes |  | control | SMG6 |
| 17 | 2203321 | T | C | rs142112207 |  | Yes |  | control | SMG6 |
| 17 | 2203323 | G | T | novel |  |  |  | control | SMG6 |
| 17 | 2203327 | A | G | novel |  | Yes |  | both | SMG6 |
| 17 | 2203343 | T | C | rs150759893 |  |  |  | control | SMG6 |
| 17 | 2203348 | T | C | rs216194 |  | Yes |  | case | SMG6 |
| 17 | 2203349 | A | G | rs151096972 | Yes |  |  | control | SMG6 |
| 17 | 2203353 | T | C | novel |  |  |  | case | SMG6 |
| 17 | 2203354 | C | G | novel |  |  |  | case | SMG6 |
| 17 | 2203360 | A | G | novel |  | Yes |  | case | SMG6 |
| 17 | 2203396 | C | T | novel |  | Yes |  | case | SMG6 |
| 17 | 2203413 | T | C | novel |  |  |  | case | SMG6 |
| 17 | 2203453 | A | G | rs216193 |  | Yes |  | both | SMG6 |
| 17 | 2203530 | G | C | novel |  |  |  | case | SMG6 |
| 17 | 2203544 | A | ACTT | novel |  |  |  | case | SMG6 |
| 17 | 2203558 | A | C | novel |  | Yes |  | case | SMG6 |
| 17 | 2203607 | C | T | novel |  |  |  | control | SMG6 |
| 17 | 2203640 | C | T | novel |  |  |  | case | SMG6 |
| 17 | 2203710 | T | C | novel | Yes |  |  | case | SMG6 |
| 17 | 2203733 | C | T | novel |  |  |  | case | SMG6 |
| 17 | 2203795 | A | G | rs202139783 |  | Yes |  | both | SMG6 |
| 17 | 2203802 | T | C | novel |  |  |  | both | SMG6 |
| 17 | 2203845 | A | G | rs202048947 | Yes |  |  | both | SMG6 |
| 17 | 2203952 | G | A | novel |  |  |  | control | SMG6 |
| 17 | 2206960 | A | G | novel |  | Yes |  | control | SMG6 |
| 17 | 2206966 | T | C | novel |  |  |  | case | SMG6 |
| 19 | 11200222 | C | A | novel |  | Yes |  | case | LDLR |
| 19 | 11200232 | T | C | novel |  |  |  | control | LDLR |
| 19 | 11200247 | G | T | novel |  |  |  | case | LDLR |
| 19 | 11200270 | T | C | novel |  |  |  | both | LDLR |
| 19 | 11200280 | T | C | novel |  |  |  | control | LDLR |
| 19 | 11210898 | C | G | novel |  | Yes | Yes | case | LDLR |
| 19 | 11210910 | C | T | novel |  | Yes |  | case | LDLR |
| 19 | 11210912 | T | C | rs2228671 |  | Yes |  | both | LDLR |
| 19 | 11210923 | T | A | novel |  |  |  | case | LDLR |
| 19 | 11210928 | T | C | rs121908024 |  | Yes | Yes | case | LDLR |
| 19 | 11210952 | A | T | novel | Yes |  |  | both | LDLR |
| 19 | 11210970 | A | G | novel | Yes | Yes |  | both | LDLR |
| 19 | 11211017 | A | G | rs55958434 |  | Yes |  | case | LDLR |
| 19 | 11213346 | C | T | novel | Yes |  |  | control | LDLR |
| 19 | 11213360 | A | G | novel | Yes |  |  | both | LDLR |
| 19 | 11213391 | A | G | novel |  |  |  | case | LDLR |
| 19 | 11213402 | T | C | novel |  | Yes | Yes | case | LDLR |
| 19 | 11213429 | A | G | novel |  | Yes |  | control | LDLR |
| 19 | 11213432 | G | T | novel |  | Yes |  | case | LDLR |
| 19 | 11213434 | T | C | rs139400379 |  | Yes |  | control | LDLR |
| 19 | 11213441 | A | G | novel |  |  |  | both | LDLR |
| 19 | 11213462 | T | C | rs13306510 |  |  |  | case | LDLR |
| 19 | 11215900 | G | C | novel |  | Yes |  | both | LDLR |
| 19 | 11215918 | T | C | novel |  | Yes |  | both | LDLR |
| 19 | 11215926 | A | G | rs201102461 | Yes |  |  | both | LDLR |
| 19 | 11215952 | T | C | novel |  |  |  | control | LDLR |
| 19 | 11215970 | A | T | novel | Yes |  |  | case | LDLR |
| 19 | 11215976 | T | C | rs368978979 |  |  |  | case | LDLR |
| 19 | 11215977 | A | G | novel | Yes |  |  | both | LDLR |
| 19 | 11215989 | A | AC | novel |  | Yes | Yes | case | LDLR |
| 19 | 11216000 | A | G | novel | Yes | Yes |  | case | LDLR |
| 19 | 11216014 | C | G | novel |  | Yes |  | control | LDLR |
| 19 | 11216033 | A | G | novel | Yes |  |  | both | LDLR |
| 19 | 11216059 | T | C | novel |  | Yes |  | case | LDLR |
| 19 | 11216060 | C | T | novel |  | Yes |  | case | LDLR |
| 19 | 11216090 | A | G | rs139089530 |  | Yes |  | case | LDLR |
| 19 | 11216092 | T | C | novel |  | Yes |  | control | LDLR |
| 19 | 11216099 | C | T | novel |  | Yes |  | case | LDLR |
| 19 | 11216125 | A | G | novel |  | Yes |  | case | LDLR |
| 19 | 11216243 | A | G | novel |  | Yes |  | case | LDLR |
| 19 | 11216255 | G | A | novel |  |  |  | both | LDLR |
| 19 | 11216258 | C | T | novel |  | Yes |  | case | LDLR |
| 19 | 11216263 | A | C | novel |  | Yes |  | case | LDLR |
| 19 | 11216264 | C | G | rs121908029 |  | Yes |  | control | LDLR |
| 19 | 11216275 | T | C | novel |  | Yes |  | both | LDLR |
| 19 | 11217263 | T | C | novel |  | Yes |  | case | LDLR |
| 19 | 11217303 | T | C | rs150673992 |  |  |  | control | LDLR |
| 19 | 11217315 | T | C | rs200990725 |  |  |  | both | LDLR |
| 19 | 11217352 | A | G | rs143992984 |  |  |  | case | LDLR |
| 19 | 11218076 | C | T | novel |  | Yes |  | control | LDLR |
| 19 | 11218079 | A | G | rs148698650 |  |  |  | control | LDLR |
| 19 | 11218104 | G | A | novel |  |  |  | both | LDLR |
| 19 | 11218154 | G | T | novel |  | Yes |  | case | LDLR |
| 19 | 11218157 | T | C | rs151207122 | Yes |  |  | case | LDLR |
| 19 | 11218158 | A | G | novel |  |  |  | case | LDLR |
| 19 | 11218159 | C | G | novel |  | Yes |  | case | LDLR |
| 19 | 11218189 | T | C | rs13306512 |  | Yes |  | both | LDLR |
| 19 | 11221334 | G | A | novel |  |  |  | case | LDLR |
| 19 | 11221356 | T | C | rs199622547 |  | Yes |  | both | LDLR |
| 19 | 11221357 | A | G | rs72658860 | Yes |  |  | case | LDLR |
| 19 | 11221389 | T | C | novel |  | Yes |  | both | LDLR |
| 19 | 11221397 | G | A | novel | Yes |  |  | both | LDLR |
| 19 | 11221399 | A | T | novel | Yes | Yes |  | case | LDLR |
| 19 | 11221425 | A | G | novel |  | Yes |  | control | LDLR |
| 19 | 11221443 | T | C | rs13306515 |  | Yes |  | both | LDLR |
| 19 | 11222191 | C | T | novel |  | Yes |  | case | LDLR |
| 19 | 11222192 | T | A | novel | Yes | Yes |  | control | LDLR |
| 19 | 11222253 | T | A | novel |  |  |  | case | LDLR |
| 19 | 11222295 | T | C | rs149227308 |  | Yes |  | control | LDLR |
| 19 | 11222305 | A | C | novel |  | Yes | Yes | case | LDLR |
| 19 | 11223961 | T | C | rs13306498 |  | Yes |  | both | LDLR |
| 19 | 11223962 | A | G | novel | Yes |  |  | case | LDLR |
| 19 | 11223969 | G | T | novel |  | Yes |  | case | LDLR |
| 19 | 11223974 | C | T | novel |  | Yes |  | case | LDLR |
| 19 | 11223977 | G | A | novel | Yes |  |  | both | LDLR |
| 19 | 11223983 | T | C | novel |  | Yes |  | case | LDLR |
| 19 | 11223997 | A | G | novel |  | Yes |  | case | LDLR |
| 19 | 11224006 | A | G | novel |  | Yes |  | both | LDLR |
| 19 | 11224013 | T | C | novel | Yes | Yes |  | case | LDLR |
| 19 | 11224014 | A | G | novel | Yes |  |  | case | LDLR |
| 19 | 11224016 | T | A | novel |  |  |  | both | LDLR |
| 19 | 11224019 | A | G | novel |  |  |  | both | LDLR |
| 19 | 11224025 | G | A | novel | Yes |  |  | both | LDLR |
| 19 | 11224030 | G | C | novel |  |  |  | control | LDLR |
| 19 | 11224051 | T | C | novel |  | Yes |  | both | LDLR |
| 19 | 11224069 | A | G | novel |  | Yes |  | case | LDLR |
| 19 | 11224095 | C | G | novel |  | Yes |  | case | LDLR |
| 19 | 11224232 | T | C | novel |  | Yes |  | case | LDLR |
| 19 | 11224233 | A | G | rs193922568 | Yes |  |  | case | LDLR |
| 19 | 11224265 | A | G | rs5930 |  | Yes |  | both | LDLR |
| 19 | 11224270 | A | T | novel |  |  |  | case | LDLR |
| 19 | 11224276 | T | C | novel |  | Yes |  | case | LDLR |
| 19 | 11224320 | T | TG | novel |  | Yes | Yes | case | LDLR |
| 19 | 11224336 | C | T | novel |  |  |  | control | LDLR |
| 19 | 11224354 | T | C | novel | Yes |  |  | both | LDLR |
| 19 | 11224368 | A | G | rs373848925 | Yes |  |  | case | LDLR |
| 19 | 11224382 | A | G | rs367655096 |  | Yes |  | both | LDLR |
| 19 | 11224391 | A | G | novel |  | Yes |  | case | LDLR |
| 19 | 11224398 | A | G | rs141673997 | Yes |  |  | both | LDLR |
| 19 | 11224415 | T | C | novel |  | Yes |  | both | LDLR |
| 19 | 11224418 | T | C | novel |  | Yes |  | case | LDLR |
| 19 | 11224434 | G | C | novel | Yes |  |  | control | LDLR |
| 19 | 11226775 | C | T | novel |  | Yes |  | case | LDLR |
| 19 | 11226798 | T | C | novel |  |  |  | control | LDLR |
| 19 | 11226800 | T | C | rs5929 |  | Yes |  | both | LDLR |
| 19 | 11226801 | A | G | novel |  | Yes |  | case | LDLR |
| 19 | 11226829 | A | G | rs28941776 |  |  |  | control | LDLR |
| 19 | 11226844 | T | C | novel |  |  |  | case | LDLR |
| 19 | 11226845 | A | G | rs151047249 |  | Yes |  | control | LDLR |
| 19 | 11226856 | C | A | novel |  |  |  | control | LDLR |
| 19 | 11226885 | G | C | novel | Yes |  |  | both | LDLR |
| 19 | 11227540 | T | C | novel |  |  |  | both | LDLR |
| 19 | 11227550 | A | G | novel |  | Yes |  | control | LDLR |
| 19 | 11227554 | T | C | rs1799898 |  | Yes |  | both | LDLR |
| 19 | 11227593 | T | C | novel |  | Yes |  | both | LDLR |
| 19 | 11227594 | A | G | rs201971888 |  |  |  | both | LDLR |
| 19 | 11227601 | G | A | novel | Yes |  |  | both | LDLR |
| 19 | 11227602 | T | C | rs688 |  | Yes |  | both | LDLR |
| 19 | 11227612 | T | C | rs373371572 | Yes | Yes |  | both | LDLR |
| 19 | 11227613 | A | G | rs201102492 |  | Yes |  | case | LDLR |
| 19 | 11227647 | T | C | novel |  | Yes |  | case | LDLR |
| 19 | 11227649 | A | AC | novel |  | Yes | Yes | case | LDLR |
| 19 | 11227651 | T | C | novel | Yes | Yes |  | case | LDLR |
| 19 | 11227663 | T | G | novel | Yes |  |  | both | LDLR |
| 19 | 11227666 | A | G | rs148181903 |  |  |  | case | LDLR |
| 19 | 11227676 | C | T | novel |  | Yes | Yes | both | LDLR |
| 19 | 11230790 | A | T | novel |  |  |  | case | LDLR |
| 19 | 11230791 | A | C | novel |  | Yes |  | both | LDLR |
| 19 | 11230798 | A | G | rs139791325 |  |  |  | control | LDLR |
| 19 | 11230818 | T | C | novel |  | Yes |  | case | LDLR |
| 19 | 11230820 | A | G | novel |  |  |  | case | LDLR |
| 19 | 11230833 | T | C | rs373570349 |  | Yes |  | case | LDLR |
| 19 | 11230880 | A | T | novel |  |  |  | case | LDLR |
| 19 | 11230881 | C | T | rs5925 |  | Yes |  | both | LDLR |
| 19 | 11230909 | A | G | novel |  |  |  | case | LDLR |
| 19 | 11231084 | A | G | novel | Yes | Yes |  | case | LDLR |
| 19 | 11231089 | T | C | novel |  | Yes |  | control | LDLR |
| 19 | 11231110 | T | C | novel |  | Yes |  | both | LDLR |
| 19 | 11231112 | T | C | rs28942084 | Yes | Yes |  | control | LDLR |
| 19 | 11231130 | T | C | rs369943481 |  | Yes |  | case | LDLR |
| 19 | 11231131 | A | G | rs146869252 |  | Yes |  | case | LDLR |
| 19 | 11233869 | G | C | novel |  | Yes |  | case | LDLR |
| 19 | 11233888 | A | G | novel |  |  |  | case | LDLR |
| 19 | 11233907 | T | C | novel |  |  |  | control | LDLR |
| 19 | 11233915 | A | G | novel |  |  |  | case | LDLR |
| 19 | 11233941 | A | G | rs5927 |  | Yes |  | both | LDLR |
| 19 | 11233948 | T | C | novel |  |  |  | control | LDLR |
| 19 | 11233992 | A | G | novel |  | Yes |  | case | LDLR |
| 19 | 11238691 | T | C | rs377563758 |  | Yes |  | case | LDLR |
| 19 | 11238695 | A | G | rs199766976 | Yes |  |  | case | LDLR |
| 19 | 11238713 | G | GAGA | novel | Yes |  |  | control | LDLR |
| 19 | 11238721 | A | G | novel |  | Yes |  | both | LDLR |
| 19 | 11238730 | T | C | rs183255090 |  | Yes |  | both | LDLR |
| 19 | 11238748 | A | T | novel |  | Yes |  | case | LDLR |
| 19 | 11238761 | A | G | novel |  |  |  | case | LDLR |
| 19 | 11240226 | G | A | rs147191787 |  | Yes |  | control | LDLR |
| 19 | 11240230 | T | A | novel |  | Yes | Yes | case | LDLR |
| 19 | 11240256 | T | C | novel |  | Yes |  | both | LDLR |
| 19 | 11240277 | T | C | novel |  | Yes |  | case | LDLR |
| 19 | 11240278 | A | G | rs137853964 |  |  |  | case | LDLR |
| 19 | 11240295 | G | A | novel |  | Yes |  | control | LDLR |
| 19 | 11241972 | G | C | novel | Yes |  |  | both | LDLR |
| 19 | 11241983 | T | C | rs139757711 |  | Yes |  | control | LDLR |
| 19 | 11241988 | T | C | rs13306505 | Yes |  |  | both | LDLR |
| 19 | 11241989 | A | G | novel |  | Yes |  | case | LDLR |

We listed 1028 variants identified in 11 genes including the 16 SNVs and 7 genes that showed an association in the discovery stage. Of those, 791 were novel and 237 were registered in dbSNP (138).

* Registered in dbSNP (138), †Also identified in the discovery stage. ‡Variants were case-specific, control-specific or identified in both groups. Abbreviations: Chr, chromosome; A1, minor allele; A2, major allele; NA, data not available

**Table S7. Top results of gene-based tests**

| gene | grouping/test method | OR | ( 95%CI ) | *P* |
| --- | --- | --- | --- | --- |
| *LDLR* | All non-synonymous/SKAT | - | - | 7.7 x 10^-4^ |
|  | Damaging /CAST | 4.4 | ( 2.6, 7.4 ) | 7.2 x 10^-10^* |
|  | Disruptive/CAST | 15 | ( 3.7, 64 ) | 5.8 x 10^-7^* |
| *PCSK9* | All non-synonymous/SKAT | - | - | 2.3 x 10^-7^* |
|  | Damaging /CAST | 0.84 | ( 0.48, 1.5 ) | 0.54 |
|  | Disruptive/CAST | 0.59 | ( 0.22, 1.6 ) | 0.29 |
|  | Gain-of-function/CAST | 1.3 | ( 1.1, 1.5 ) | 1.0 x 10^-4^* |
|  | Loss-of-function/CAST | 0.66 | ( 0.53, 0.81 ) | 1.1 x 10^-4^* |

We represented results of gene-based tests for *LDLR* and *PCSK9*. These results are meta-analysis of the discovery and replication stages. For SKAT, meta-analysis was done by the MetaSKAT R package. For CAST, meta-analysis was done by the Metafor R package using the Cochran-Mantel-Haenszel method. OR: odds ratio, CI: confidential interval, * means the study-wide significance, Gain-of-function: variants that registered as gain-of-function variants on the database, Loss-of-function: variants that registered as loss-of-function variants on the database or disruptive variant.

**Table S8. Identified *LDLR* variants in the familial hypercholesterolemia database^23^**

| Chr | BP | Ref | Alt | Type | Cases | Controls | Group | AA change  or splice-site change | LDLR  FH database ID | FH allele name | Allele Frequency  in ExAC |
| --- | --- | --- | --- | --- | --- | --- | --- | --- | --- | --- | --- |
| 19 | 11200232 | C | T | missense | 0 | 1 | Non synonymous | P3L |  |  |  |
| 19 | 11200247 | T | G | missense | 1 | 0 | Non synonymous | L8W |  |  |  |
| 19 | 11200270 | C | T | missense | 1 | 1 | Non synonymous | L16F |  |  |  |
| 19 | 11200280 | C | T | missense | 0 | 1 | Non synonymous | A19V |  |  |  |
| 19 | 11200282 | G | A | missense | 0 | 1 | Non synonymous | G20R | LDLR_00868 |  | 0.001046 |
| 19 | 11210898 | G | C | splice-site | 1 | 0 | Disruptive | -1G>C | LDLR_00277 |  |  |
| 19 | 11210902 | G | A | missense | 1 | 0 | Non synonymous | G24D |  |  |  |
| 19 | 11210910 | T | C | missense | 1 | 0 | Damaging | C27R |  |  |  |
| 19 | 11210923 | A | T | missense | 1 | 0 | Non synonymous | E31V |  |  |  |
| 19 | 11210928 | C | T | nonsense | 1 | 0 | Disruptive | Q33* | LDLR_00005 | FH Turkey/Milan-4 | 1.65E-05 |
| 19 | 11210952 | T | A | missense | 4 | 3 | Non synonymous | S41T |  |  |  |
| 19 | 11210970 | G | A | missense | 3 | 1 | Damaging | D47N | LDLR_00383 | FH Hyogo | 1.65E-05 |
| 19 | 11210982 | G | A | missense | 0 | 2 | Damaging | E51K |  |  |  |
| 19 | 11213346 | T | C | missense | 3 | 2 | Non synonymous | V66A |  |  |  |
| 19 | 11213360 | G | A | missense | 5 | 1 | Non synonymous | G71R |  |  | 1.65E-05 |
| 19 | 11213391 | G | A | missense | 1 | 0 | Non synonymous | R81H |  |  |  |
| 19 | 11213402 | C | T | nonsense | 1 | 0 | Disruptive | Q85* | LDLR_00184 |  |  |
| 19 | 11213429 | G | A | missense | 0 | 1 | Damaging | D94N |  |  | 8.24E-06 |
| 19 | 11213432 | T | G | missense | 1 | 0 | Damaging | C95G | LDLR_00454 |  |  |
| 19 | 11213434 | C | A | nonsense | 1 | 0 | Disruptive | C95* | LDLR_00306 |  |  |
| 19 | 11213441 | G | A | missense | 1 | 1 | Non synonymous | G98S | LDLR_01032 |  | 5.77E-05 |
| 19 | 11213462 | C | T | missense | 2 | 0 | Non synonymous | P105S | LDLR_00442 |  | 6.59E-05 |
| 19 | 11215926 | G | A | missense | 61 | 62 | Non synonymous | R74H | LDLR_00366 |  | 0.000184 |
| 19 | 11215943 | T | G | missense | 2 | 0 | Damaging | C80G | LDLR_00935 |  |  |
| 19 | 11215952 | C | T | missense | 0 | 2 | Non synonymous | R83W | LDLR_00692 |  |  |
| 19 | 11215964 | TG | T | indel frameshift | 1 | 0 | Disruptive | C128fs |  |  |  |
| 19 | 11215970 | T | A | missense | 4 | 0 | Non synonymous | S89T | LDLR_00936 |  |  |
| 19 | 11215976 | C | T | missense | 1 | 0 | Non synonymous | R91W |  |  | 8.29E-06 |
| 19 | 11215977 | G | A | missense | 7 | 2 | Non synonymous | R91Q |  |  | 2.49E-05 |
| 19 | 11215989 | AC | A | indel frameshift | 1 | 0 | Disruptive | D136fs |  |  |  |
| 19 | 11216000 | G | A | missense | 2 | 1 | Damaging | E99K | LDLR_00413 | FH Sassari-2 |  |
| 19 | 11216007 | C | T | missense | 0 | 1 | Non synonymous | S142F |  |  |  |
| 19 | 11216009 | T | G | missense | 1 | 0 | Damaging | C143G |  |  |  |
| 19 | 11216033 | G | A | missense | 4 | 2 | Non synonymous | A151T | LDLR_01038 |  |  |
| 19 | 11216060 | T | C | missense | 1 | 0 | Damaging | C160R | LDLR_00721 |  |  |
| 19 | 11216090 | G | A | missense | 1 | 0 | Damaging | D170N |  |  | 8.26E-06 |
| 19 | 11216099 | T | C | missense | 1 | 0 | Damaging | C173R | LDLR_00023 | FH Greece-1, FH Canada |  |
| 19 | 11216147 | G | A | missense | 1 | 0 | Non synonymous | V189M | LDLR_00938 |  |  |
| 19 | 11216243 | G | A | missense | 1 | 0 | Damaging | D221N | LDLR_00490 |  |  |
| 19 | 11216255 | A | G | missense | 2 | 1 | Non synonymous | K225E |  |  |  |
| 19 | 11216258 | T | C | missense | 1 | 0 | Damaging | S226P | LDLR_00035 | FH Miami-1 |  |
| 19 | 11216263 | C | A | missense | 1 | 0 | Damaging | D227E |  |  |  |
| 19 | 11216264 | G | C | missense | 0 | 1 | Damaging | E228Q | LDLR_00038 | FH Tulsa-2, FH Iraq | 8.55E-06 |
| 19 | 11217303 | C | T | missense | 0 | 1 | Non synonymous | R253W | LDLR_00375 |  | 0.000181 |
| 19 | 11217315 | C | T | missense | 2 | 1 | Non synonymous | R257W | LDLR_00712 |  | 7.41E-05 |
| 19 | 11217352 | G | A | missense | 1 | 0 | Non synonymous | G269D | LDLR_00504 | FH Rome-3 | 0.00019 |
| 19 | 11218074 | T | C | missense | 0 | 1 | Non synonymous | L275P |  |  |  |
| 19 | 11218076 | T | C | missense | 0 | 1 | Damaging | C276R |  |  |  |
| 19 | 11218079 | G | A | missense | 0 | 1 | Non synonymous | E277K | LDLR_00195 | FH Walloon, Genoa-3 | 0.000503 |
| 19 | 11218104 | A | G | missense | 3 | 1 | Non synonymous | H285R |  |  |  |
| 19 | 11218154 | T | G | missense | 1 | 0 | Damaging | C302G |  |  |  |
| 19 | 11218157 | C | T | missense | 1 | 1 | Non synonymous | R303W | LDLR_00512 |  | 4.12E-05 |
| 19 | 11218158 | G | A | missense | 1 | 0 | Non synonymous | R303Q |  |  | 8.24E-06 |
| 19 | 11221334 | A | G | missense | 1 | 0 | Non synonymous | N316S |  |  | 3.32E-05 |
| 19 | 11221357 | G | A | missense | 1 | 3 | Non synonymous | G324S | LDLR_00640 |  | 0.001163 |
| 19 | 11221364 | C | A | missense | 1 | 0 | Non synonymous | S326Y |  |  |  |
| 19 | 11221397 | A | G | missense | 5 | 4 | Non synonymous | E337G |  |  |  |
| 19 | 11221399 | T | A | missense | 8 | 1 | Damaging | C338S | LDLR_00280 | FH Wakayama |  |
| 19 | 11222190 | A | AT | indel frameshift | 1 | 0 | Disruptive | D354fs |  |  |  |
| 19 | 11222192 | A | T | missense | 2 | 2 | Damaging | I187F |  |  |  |
| 19 | 11222213 | G | A | missense | 1 | 0 | Non synonymous | D362N |  |  |  |
| 19 | 11222253 | A | T | missense | 1 | 0 | Non synonymous | Y375F |  |  |  |
| 19 | 11222265 | G | A | missense | 1 | 0 | Damaging | C379Y | LDLR_00364 |  |  |
| 19 | 11222295 | C | T | missense | 0 | 1 | Damaging | T389M | LDLR_00884 |  | 1.67E-05 |
| 19 | 11222305 | C | A | nonsense | 1 | 0 | Disruptive | C392* | LDLR_00157 |  | 8.35E-06 |
| 19 | 11223954 | G | C | missense | 1 | 0 | Non synonymous | G228A |  |  | 2.51E-05 |
| 19 | 11223962 | G | A | missense | 2 | 0 | Non synonymous | A399T | LDLR_00534 | FH Nuoro | 8.36E-06 |
| 19 | 11223969 | T | G | missense | 1 | 0 | Damaging | L401R |  |  |  |
| 19 | 11223974 | T | C | missense | 1 | 0 | Damaging | F403L | LDLR_00536 | FH Wakayama |  |
| 19 | 11223977 | A | G | missense | 4 | 1 | Non synonymous | T404A |  |  |  |
| 19 | 11223983 | C | T | missense | 1 | 0 | Damaging | R406W | LDLR_00451 | FH Rumania | 1.67E-05 |
| 19 | 11224013 | C | T | missense | 1 | 0 | Damaging | R416W | LDLR_00216 |  | 2.49E-05 |
| 19 | 11224014 | G | A | missense | 5 | 0 | Non synonymous | R416Q | LDLR_00379 |  | 1.66E-05 |
| 19 | 11224016 | A | T | missense | 2 | 1 | Non synonymous | S417C |  |  |  |
| 19 | 11224017 | G | A | missense | 1 | 0 | Non synonymous | S417N |  |  |  |
| 19 | 11224019 | G | A | missense | 2 | 3 | Non synonymous | E418K | LDLR_00942 |  |  |
| 19 | 11224025 | A | G | missense | 11 | 6 | Non synonymous | T420A |  |  |  |
| 19 | 11224030 | C | G | missense | 0 | 1 | Non synonymous | S421R |  |  |  |
| 19 | 11224095 | G | C | missense | 1 | 0 | Damaging | W443S | LDLR_00779 |  |  |
| 19 | 11224233 | G | A | missense | 1 | 1 | Non synonymous | G461S |  |  | 1.66E-05 |
| 19 | 11224270 | T | A | missense | 2 | 0 | Non synonymous | I473N | LDLR_00295 |  |  |
| 19 | 11224276 | C | T | missense | 1 | 0 | Damaging | A475V |  |  |  |
| 19 | 11224320 | TG | T | indel frameshift | 1 | 0 | Disruptive | W490fs |  |  |  |
| 19 | 11224326 | G | A | missense | 1 | 0 | Damaging | D492N | LDLR_00074 | FH Cincinnati-2 | 1.65E-05 |
| 19 | 11224336 | T | C | missense | 0 | 1 | Non synonymous | L495P |  |  |  |
| 19 | 11224354 | C | T | missense | 4 | 3 | Non synonymous | A501V | LDLR_00899 |  | 8.24E-06 |
| 19 | 11224368 | G | A | missense | 4 | 0 | Non synonymous | V506M |  |  | 8.24E-05 |
| 19 | 11224398 | G | A | missense | 9 | 3 | Non synonymous | G516S |  |  | 7.43E-05 |
| 19 | 11224434 | C | G | missense | 0 | 4 | Non synonymous | H528D |  |  |  |
| 19 | 11226775 | T | C | missense | 1 | 0 | Damaging | M531T |  |  |  |
| 19 | 11226798 | C | T | missense | 0 | 1 | Non synonymous | P539S |  |  |  |
| 19 | 11226801 | G | A | missense | 1 | 0 | Damaging | A540T | LDLR_00243 |  | 1.65E-05 |
| 19 | 11226829 | G | A | missense | 0 | 1 | Non synonymous | G549D | LDLR_00232 |  | 4.94E-05 |
| 19 | 11226844 | C | T | missense | 1 | 0 | Non synonymous | S554L |  |  |  |
| 19 | 11226856 | A | C | missense | 0 | 1 | Non synonymous | E558A |  |  |  |
| 19 | 11226883 | C | A | missense | 1 | 0 | Non synonymous | T567N |  |  |  |
| 19 | 11226885 | C | G | missense | 33 | 5 | Non synonymous | L568V | LDLR_00558 |  |  |
| 19 | 11227540 | C | T | missense | 3 | 1 | Non synonymous | L571F |  |  | 8.24E-06 |
| 19 | 11227547 | G | A | missense | 0 | 1 | Non synonymous | G573D |  |  |  |
| 19 | 11227550 | G | A | missense | 0 | 1 | Damaging | R574H |  |  | 8.24E-06 |
| 19 | 11227594 | G | A | missense | 1 | 1 | Non synonymous | D589N | LDLR_01066 |  | 5.77E-05 |
| 19 | 11227601 | A | G | missense | 3 | 1 | Non synonymous | N591S |  |  | 8.24E-06 |
| 19 | 11227612 | C | T | missense | 5 | 2 | Damaging | R595W | LDLR_01207 |  | 8.24E-06 |
| 19 | 11227613 | G | A | missense | 1 | 0 | Damaging | R595Q | LDLR_00563 |  | 4.94E-05 |
| 19 | 11227649 | AC | A | indel frameshift | 1 | 0 | Disruptive | H607fs |  |  |  |
| 19 | 11227651 | C | T | missense | 3 | 0 | Damaging | P608S | LDLR_00662 |  |  |
| 19 | 11227663 | G | T | missense | 6 | 2 | Non synonymous | A612S |  |  |  |
| 19 | 11227666 | G | A | missense | 1 | 0 | Non synonymous | V613I | LDLR_01070 |  | 1.65E-05 |
| 19 | 11227676 | T | C | splice-site | 14 | 1 | Disruptive | +2T>C | LDLR_00087 | FH Niigata | 8.24E-06 |
| 19 | 11230790 | T | A | missense | 1 | 0 | Non synonymous | I623N |  |  |  |
| 19 | 11230798 | G | A | missense | 0 | 2 | Non synonymous | E626K |  |  | 0.000486 |
| 19 | 11230802 | C | T | missense | 1 | 0 | Non synonymous | A627V | LDLR_00658 |  |  |
| 19 | 11230820 | G | A | missense | 1 | 0 | Non synonymous | R633H | LDLR_01073 |  | 1.65E-05 |
| 19 | 11230880 | T | A | missense | 1 | 0 | Non synonymous | V653D |  |  |  |
| 19 | 11230909 | G | A | missense | 1 | 0 | Non synonymous | E536K |  |  |  |
| 19 | 11231056 | G | C | missense | 1 | 0 | Damaging | W666C |  |  |  |
| 19 | 11231084 | G | A | missense | 3 | 0 | Damaging | G676S | LDLR_00946 |  | 1.66E-05 |
| 19 | 11231112 | C | T | missense | 2 | 1 | Damaging | P685L | LDLR_00094 | FH Gujerat, FH Frosinone1, FH Kanazawa-2 | 6.61E-05 |
| 19 | 11231130 | C | T | missense | 1 | 0 | Damaging | S691L |  |  | 3.3E-05 |
| 19 | 11231146 | C | A | nonsense | 1 | 0 | Disruptive | C696* | LDLR_00906 |  |  |
| 19 | 11233888 | G | A | missense | 2 | 0 | Non synonymous | V727I |  |  | 4.13E-05 |
| 19 | 11233907 | C | T | missense | 0 | 1 | Non synonymous | S733F |  |  |  |
| 19 | 11233915 | G | A | missense | 3 | 0 | Non synonymous | V736I |  |  | 2.48E-05 |
| 19 | 11233931 | C | G | missense | 0 | 1 | Non synonymous | T741R |  |  |  |
| 19 | 11233948 | C | T | missense | 0 | 1 | Non synonymous | P747S |  |  |  |
| 19 | 11233966 | C | T | missense | 1 | 0 | Non synonymous | P753S |  |  |  |
| 19 | 11238695 | G | A | missense | 1 | 1 | Non synonymous | V775I |  |  | 2.47E-05 |
| 19 | 11238705 | G | T | missense | 1 | 0 | Non synonymous | R778I |  |  | 8.24E-06 |
| 19 | 11238713 | GAGA | G | indel | 1 | 2 | Non synonymous | EK781E |  |  | 8.24E-06 |
| 19 | 11238731 | G | A | missense | 0 | 1 | Non synonymous | V787M |  |  |  |
| 19 | 11238761 | G | A | missense | 1 | 0 | Non synonymous | V619M | LDLR_00259 |  | 8.25E-06 |
| 19 | 11240215 | GTC | AGAAG | indel frameshift | 0 | 1 | Disruptive | V806fs |  |  |  |
| 19 | 11240230 | A | T | nonsense | 11 | 0 | Disruptive | K811* | LDLR_00281 | FH Tokyo |  |
| 19 | 11240240 | G | A | missense | 1 | 0 | Non synonymous | R814Q | LDLR_00376 | FH Xhosa | 0.00028 |
| 19 | 11240278 | G | A | missense | 2 | 0 | Non synonymous | V827I | LDLR_00101 | FH New York-5 | 0.000758 |
| 19 | 11241972 | C | G | missense | 9 | 13 | Non synonymous | L855V |  |  | 8.24E-06 |
| 19 | 11241988 | C | T | missense | 136 | 152 | Non synonymous | A860V |  |  | 6.59E-05 |

Abbreviations: Chr, chromosome; Ref, reference allele (hg19), Alt, alternative allele; Type, variant type; cases, number of carriers in cases; controls, number of carriers in controls; AA, amino acid; FH, Familial Hypercholesterolemia.

**Table S9. Identified *PCSK9* variants in the familial hypercholesterolemia database^24^**

| Chr | Position | rsID(dbSNP138) | Ref | Alt | Type | cases | controls | Group | AA change | *PCSK9* FH database ID | Predicted Effect^*^ | Allele Frequency  in ExAC |
| --- | --- | --- | --- | --- | --- | --- | --- | --- | --- | --- | --- | --- |
| 1 | 55505520 | rs186669805 | G | A | missense | 245 | 204 | Non synonymous | V4I | PCSK9_00006 | GoF | 0.000268 |
| 1 | 55505532 |  | C | G | missense | 0 | 1 | Non synonymous | R8G |  |  |  |
| 1 | 55505552 | rs113330492 | A | ACTG | insertion | 2310 | 2038 | Non synonymous | L15LL |  |  | 0.1375 |
| 1 | 55505581 |  | G | A | missense | 2 | 1 | Non synonymous | G24D |  |  |  |
| 1 | 55505596 |  | G | A | missense | 2 | 0 | Non synonymous | R29H |  |  |  |
| 1 | 55505601 |  | C | A | missense | 1 | 0 | Non synonymous | Q31K |  |  |  |
| 1 | 55505604 |  | G | A | missense | 292 | 147 | Non synonymous | E32K | PCSK9_00008 | GoF | 7.69E-05 |
| 1 | 55505625 |  | G | A | missense | 1 | 0 | Non synonymous | E39K |  |  |  |
| 1 | 55505668 | rs11583680 | C | T | missense | 2336 | 2056 | Non synonymous | A53V | PCSK9_00011 |  | 0.1914 |
| 1 | 55505671 |  | A | C | missense | 3 | 6 | Non synonymous | E54A | PCSK9_00012 | GoF |  |
| 1 | 55505684 |  | C | A | missense | 1 | 0 | Non synonymous | H58Q |  |  |  |
| 1 | 55505692 |  | CA | C | indel frameshift | 1 | 0 | Disruptive | T61fs |  |  |  |
| 1 | 55505712 |  | G | A | missense | 9 | 4 | Non synonymous | A68T | PCSK9_00014 |  | 3.72E-05 |
| 1 | 55509520 |  | C | T | missense | 0 | 2 | Non synonymous | P71L |  |  | 9.18E-05 |
| 1 | 55509543 |  | G | A | missense | 1 | 2 | Non synonymous | V79M |  |  | 1.66E-05 |
| 1 | 55509561 |  | G | A | missense | 4 | 1 | Non synonymous | E85K |  |  | 2.48E-05 |
| 1 | 55509577 |  | A | G | missense | 1 | 0 | Non synonymous | Q90R |  |  |  |
| 1 | 55509582 |  | G | C | missense | 1 | 0 | Non synonymous | E92Q |  |  | 1.65E-05 |
| 1 | 55509585 | rs151193009 | C | T | missense | 134 | 177 | Non synonymous | R93C | PCSK9_00019 | LoF | 0.000587 |
| 1 | 55509594 | rs185392267 | C | T | missense | 1 | 1 | Non synonymous | R96C |  |  | 8.27E-06 |
| 1 | 55509598 | rs376385276 | G | A | missense | 0 | 1 | Non synonymous | R97H |  |  | 0.000174 |
| 1 | 55509606 |  | G | C | missense | 0 | 1 | Non synonymous | A100P |  |  |  |
| 1 | 55509618 | rs369067856 | C | T | missense | 1 | 0 | Non synonymous | R104C | PCSK9_00021 | GoF | 2.48E-05 |
| 1 | 55509630 |  | C | T | missense | 1 | 0 | Non synonymous | L108F |  |  |  |
| 1 | 55509648 |  | G | A | missense | 0 | 1 | Non synonymous | V114I |  |  |  |
| 1 | 55509661 |  | T | C | missense | 1 | 1 | Non synonymous | L118P |  |  |  |
| 1 | 55509704 |  | G | C | missense | 2 | 0 | Non synonymous | E132D |  |  |  |
| 1 | 55512254 |  | G | A | missense | 0 | 1 | Non synonymous | S153N |  |  |  |
| 1 | 55512299 |  | C | T | missense | 5 | 9 | Non synonymous | A168V |  |  | 1.66E-05 |
| 1 | 55517990 |  | G | T | missense | 0 | 0 | Damaging | S188I |  |  |  |
| 1 | 55518006 |  | C | A | missense | 1 | 0 | Damaging | H193Q |  |  |  |
| 1 | 55518016 |  | G | A | missense | 0 | 1 | Non synonymous | E197K |  |  | 8.25E-06 |
| 1 | 55518029 |  | T | G | missense | 0 | 1 | Non synonymous | M201R |  |  |  |
| 1 | 55518070 |  | C | T | missense | 1 | 1 | Damaging | R215C |  |  | 1.66E-05 |
| 1 | 55518071 |  | G | A | missense | 2 | 0 | Non synonymous | R215H | PCSK9_00036 | GoF |  |
| 1 | 55518082 |  | C | G | missense | 3 | 5 | Non synonymous | Q219E | PCSK9_00039 | LoF | 2.49E-05 |
| 1 | 55518329 |  | A | G | missense | 0 | 1 | Non synonymous | K222E |  |  |  |
| 1 | 55518362 | rs150169598 | G | T | missense | 1 | 0 | Non synonymous | V233L |  |  | 8.4E-06 |
| 1 | 55518374 | rs148195424 | C | T | missense | 0 | 3 | Non synonymous | R237W | PCSK9_00047 |  | 0.000597 |
| 1 | 55518375 |  | G | T | missense | 0 | 1 | Non synonymous | R237L |  |  |  |
| 1 | 55518381 |  | C | A | missense | 0 | 1 | Damaging | A239D | PCSK9_00048 | LoF |  |
| 1 | 55518383 |  | G | A | missense | 0 | 1 | Damaging | G240S |  |  | 8.41E-06 |
| 1 | 55518386 |  | G | C | missense | 0 | 1 | Damaging | V241L |  |  |  |
| 1 | 55518407 |  | C | T | missense | 0 | 1 | Non synonymous | R248C |  |  | 5.9E-05 |
| 1 | 55518417 | rs376945520 | G | A | missense | 2 | 0 | Non synonymous | R251H |  |  | 4.21E-05 |
| 1 | 55518452 | rs200146448 | G | A | missense | 130 | 120 | Non synonymous | G263S | PCSK9_00051 |  | 2.53E-05 |
| 1 | 55518456 | rs201789841 | C | T | missense | 124 | 100 | Non synonymous | T264I |  |  | 0.000126 |
| 1 | 55521683 |  | A | G | missense | 0 | 1 | Non synonymous | K273E |  |  |  |
| 1 | 55521716 |  | G | A | missense | 1 | 0 | Non synonymous | V284M |  |  |  |
| 1 | 55521783 |  | G | T | missense | 0 | 1 | Non synonymous | R306M |  |  |  |
| 1 | 55521794 |  | G | A | missense | 1 | 1 | Non synonymous | V310M |  |  |  |
| 1 | 55523034 |  | G | A | missense | 0 | 1 | Damaging | D343N |  |  |  |
| 1 | 55523076 | rs148562777 | C | T | missense | 0 | 1 | Non synonymous | R357C |  |  | 0.000149 |
| 1 | 55523119 |  | C | T | missense | 0 | 0 | Non synonymous | A371V |  |  |  |
| 1 | 55523141 |  | C | G | missense | 0 | 1 | Damaging | C378W |  |  | 8.6E-06 |
| 1 | 55523188 |  | G | A | splice-site | 1 | 2 | Disruptive |  |  |  |  |
| 1 | 55523798 |  | A | G | missense | 111 | 129 | Non synonymous | I424V | PCSK9_00064 |  | 9.13E-05 |
| 1 | 55523808 |  | C | A | missense | 1 | 0 | Non synonymous | A427D |  |  |  |
| 1 | 55523812 |  | G | A | nonsense | 4 | 5 | Disruptive | W428* | PCSK9_00066 | LoF |  |
| 1 | 55523829 |  | G | A | missense | 1 | 0 | Non synonymous | R434Q |  |  |  |
| 1 | 55523873 |  | C | T | missense | 0 | 1 | Non synonymous | H449Y |  |  |  |
| 1 | 55524237 | rs562556 | A | G | missense | 625 | 511 | Non synonymous | V474I | PCSK9_00074 |  |  |
| 1 | 55524244 | rs376388695 | G | A | missense | 1 | 0 | Non synonymous | R476H |  |  | 2.49E-05 |
| 1 | 55524246 |  | T | A | missense | 1 | 0 | Damaging | C477S |  |  |  |
| 1 | 55524300 |  | C | T | missense | 0 | 2 | Non synonymous | R495W |  |  | 1.8E-05 |
| 1 | 55524303 | rs374603772 | C | T | missense | 1 | 2 | Non synonymous | R496W | PCSK9_00076 | GoF | 7.31E-05 |
| 1 | 55524304 | rs139669564 | G | A | missense | 1 | 0 | Non synonymous | R496Q | PCSK9_00077 |  | 0.000347 |
| 1 | 55524312 | rs201395805 | C | T | missense | 2 | 6 | Non synonymous | R499C |  |  | 0.000197 |
| 1 | 55525165 | rs374455190 | G | T | missense | 1 | 1 | Non synonymous | G504W |  |  | 4.47E-05 |
| 1 | 55525183 |  | C | T | missense | 1 | 0 | Non synonymous | R510W |  |  |  |
| 1 | 55525195 |  | G | A | missense | 6 | 3 | Non synonymous | A514T | PCSK9_00078 | GoF |  |
| 1 | 55525219 |  | G | A | missense | 4 | 2 | Damaging | A522T | PCSK9_00080 | GoF | 8.62E-05 |
| 1 | 55525241 |  | T | G | missense | 1 | 0 | Non synonymous | L529R |  |  |  |
| 1 | 55525261 |  | G | A | missense | 0 | 1 | Non synonymous | V536I |  |  | 0.000469 |
| 1 | 55525295 |  | G | T | missense | 0 | 1 | Non synonymous | G547V |  |  |  |
| 1 | 55527059 |  | C | T | missense | 1 | 1 | Non synonymous | H565Y |  |  |  |
| 1 | 55527062 |  | T | A | missense | 2 | 0 | Non synonymous | W566R |  |  |  |
| 1 | 55527093 | rs72646525 | C | T | missense | 4 | 4 | Non synonymous | P576L |  |  | 0.000163 |
| 1 | 55527110 | rs373323910 | C | T | nonsense | 1 | 0 | Disruptive | R582* |  |  | 2.4E-05 |
| 1 | 55527131 | rs372586224 | G | A | missense | 5 | 5 | Non synonymous | V589M |  |  | 1.28E-05 |
| 1 | 55527141 |  | G | C | missense | 2 | 1 | Non synonymous | R592T |  |  |  |
| 1 | 55527158 | rs367606156 | G | A | missense | 3 | 4 | Non synonymous | A598T |  |  | 0.000112 |
| 1 | 55527174 |  | C | G | missense | 1 | 1 | Non synonymous | A603G |  |  |  |
| 1 | 55527190 |  | CAA | C | indel frameshift | 0 | 1 | Disruptive | K609fs |  |  |  |
| 1 | 55527204 |  | A | C | missense | 0 | 1 | Non synonymous | H613P |  |  |  |
| 1 | 55529048 |  | G | A | missense | 1 | 1 | Non synonymous | V624M | PCSK9_00089 | GoF | 4.71E-05 |
| 1 | 55529064 |  | G | A | missense | 10 | 7 | Damaging | G629D |  |  |  |
| 1 | 55529108 | rs143291739 | G | A | missense | 65 | 66 | Non synonymous | V644I | PCSK9_00091 |  | 4.27E-05 |
| 1 | 55529132 | rs201280059 | A | G | missense | 2 | 1 | Non synonymous | N652D |  |  | 5.83E-05 |
| 1 | 55529153 |  | C | G | missense | 10 | 8 | Non synonymous | R659G |  |  | 3.32E-05 |
| 1 | 55529182 |  | C | A | missense | 15 | 8 | Non synonymous | S668R | PCSK9_00093 | LoF | 3.33E-05 |
| 1 | 55529187 | rs505151 | A | G | missense | 932 | 720 | Non synonymous | G670E | PCSK9_00094 |  |  |
| 1 | 55529217 |  | G | A | missense | 0 | 1 | Non synonymous | R680Q |  |  |  |
| 1 | 55529243 |  | C | T | nonsense | 0 | 2 | Disruptive | Q689* |  |  |  |

Abbreviations: Chr, chromosome; Ref, reference allele (hg19), Alt, alternative allele; Type, variant type; cases, number of carriers in cases; controls, number of carriers in controls; AA, amino acid; FH, Familial Hypercholesterolemia;

^*^Predicted effect is based on FH database^24^; LoF: predicted as a loss-of-function variant; GoF: predicted as a gain-of-function variant

**Table S10. The numbers of carriers after exclusion of known variants**.

| Gene | Category | Stage | Before | | After Exclusion | |
| --- | --- | --- | --- | --- | --- | --- |
|  |  |  | case | control | case | control |
| *LDLR* | Disruptive | Discovery | 10 | 1 | 2 | 1 |
|  |  | Replication | 26 | 1 | 3 | 0 |
|  |  | Combine | 36 | 2 | 5 | 1 |
| *LDLR* | Damaging | Discovery | 28 | 5 | 6 | 3 |
|  |  | Replication | 61 | 12 | 10 | 5 |
|  |  | Combine | 89 | 17 | 16 | 8 |
| *PCSK9* | All Non synonymous | Discovery | 732 | 754 | 40 | 45 |
|  |  | Replication | 1,874 | 1,388 | 104 | 88 |
|  |  | Combine | 2,606 | 2,142 | 144 | 133 |

To examine the effect of newly identified variants in this study, we subtracted these known sequencing variants from our data and explored how these associations changed among three categories which showed an exome-wide significant association with MI.

**Table S11. Effects of newly-identified *LDLR* and *PCSK9* rare variants on LDL-cholesterol levels.**

| Gene | Category | n | LDL-C (mg/dl) | Change from Non-Carriers of Rare *LDLR*/*PCSK9* Variants | | | |
| --- | --- | --- | --- | --- | --- | --- | --- |
|  |  |  | mean ± SD | Crude (95%CI) | *P* | Adjusted† (95%CI) | *P* |
| Non-Carriers of Rare *LDLR*/*PCSK9* Variants | | 8283 | 113.47 ± 31.42 |  |  |  |  |
| *LDLR* | Non synonymous | 224 | 117.27 ± 34.36 | +3.80 (-2.17, +9.76) | 0.21 | +3.16 (-2.94, +9.25) | 0.31 |
| *LDLR* | Damaging | 7 | 192.67 ± 76.11 | +79.19 (+43.57, +114.81) | 1.34 x 10^-5^ | +39.57 (-2.73, +81.86) | 0.067 |
| *LDLR* | Disruptive | 3 | 180.20 ± 13.01 | +66.73 (+23.16, +110.29) | 2.70 x 10^-3^ | +71.80 (+29.52, +114.09) | 8.80 x 10^-4^ |
| *PCSK9* | Disruptive | 6 | 56.40 ± NA | -57.07 (-118.68, + 4.53) | 0.069 | -59.58 (-119.32, +0.17) | 0.051 |

Changes from non-carriers and confidence intervals were calculated from linear regression models.

† Adjusted for age, gender, BMI, smoking status and cholesterol lowering medications.

Abbreviations: LDL-C, low-density lipoprotein cholesterol; SD, standard deviation; 95%CI, 95% confidence interval.

**Table S12. Effects of *LDLR* and *PCSK9* rare variants on LDL-cholesterol levels in the subjects without cholesterol-lowering drugs.**

| Gene | Category | N | LDL-C (mg/dl) | Change from Non-Carriers of Rare *LDLR*/*PCSK9* Variants | | | |
| --- | --- | --- | --- | --- | --- | --- | --- |
|  |  |  | mean ± SD | Crude (95%CI) | *P* | Adjusted† (95%CI) | *P* |
| Non-Carriers of  Rare *LDLR*/*PCSK9* Variants | | 2930 | 117.12 ± 31.21 |  |  |  |  |
| *LDLR* | non-synonymous | 140 | 120.00 ± 36.38 | +2.88 (-2.46, +8.22) | 0.29 | +2.72 (-2.53, +7.97) | 0.31 |
| *LDLR* | Damaging | 12 | 144.67 ± 42.45 | +27.55 (+9.81, +45.28) | 2.3 x 10^-3^ | +28.07 (+10.66, +45.48) | 1.6 x 10^-3^ |
| *LDLR* | Disruptive | 2 | 148.60 ± 13.86 | +31.48 (-11.81, +74.77) | 0.15 | +33.78 (-8.74, +76.29) | 0.12 |
| *PCSK9* | E32K | 73 | 134.27 ± 39.05 | +17.15 (+9.84, +24.45) | 4.3 x 10^-6^ | +16.66 (+9.48, +23.83) | 5.56 x 10^-6^ |
| *PCSK9* | R93C | 75 | 105.62 ± 33.63 | -11.50 (-18.67, -4.33) | 1.7 x 10^-3^ | -11.54 (-18.59, -4.49) | 1.3 x 10^-3^ |
| *PCSK9* | Disruptive | 4 | 80.70 ± 27.17 | -36.42 (-67.04, -5.80) | 0.02 | -38.74 (-68.82, -8.67) | 0.012 |

Changes from non-carriers and confidence intervals were calculated from linear regression models.

† Adjusted for age, gender, BMI, smoking status.

Abbreviations: LDL-C, low-density lipoprotein cholesterol; SD, standard deviation; 95%CI, 95% confidence interval.

**Table S13. Effects of *LDLR* and *PCSK9* rare variants on LDL-cholesterol levels in the subjects with cholesterol-lowering drugs.**

| Gene | Category | N | LDL-C (mg/dl) | Change from Non-Carriers of Rare *LDLR*/*PCSK9* Variants | | | |
| --- | --- | --- | --- | --- | --- | --- | --- |
|  |  |  | mean ± SD | Crude (95%CI) | *P* | Adjusted† (95%CI) | *P* |
| Non-Carriers of  Rare *LDLR*/*PCSK9* Variants | | 2150 | 106.09 ± 31.03 |  |  |  |  |
| *LDLR* | non-synonymous | 121 | 113.88 ± 39.66 | +7.79 (+2.01, +13.57) | 8.3 x 10^-3^ | +7.44 (+1.71, +13.18) | 0.011 |
| *LDLR* | Damaging | 22 | 160.76 ± 33.67 | +54.67 (+41.62, +67.73) | 3.61 x 10^-16^ | +53.52 (+40.57, +66.47) | 8.75 x 10^-16^ |
| *LDLR* | Disruptive | 19 | 156.53 ± 35.57 | +50.44 (+36.4, +64.48) | 2.50 x 10^-12^ | +49.93 (+35.96, +63.89) | 3.18 x 10^-12^ |
| *PCSK9* | E32K | 113 | 125.17 ± 44.5 | +19.08 (+13.05, +25.10) | 6.31 x 10^-10^ | +18.67 (+12.7, +24.64) | 1.00 x 10^-9^ |
| *PCSK9* | R93C | 27 | 92.61 ± 37.24 | -13.47 (-25.29, -1.66) | 0.025 | -14.5 (-26.23, -2.77) | 0.015 |
| *PCSK9* | Disruptive | 0 | NA | NA | NA | NA | NA |

Changes from non-carriers and confidence intervals were calculated from linear regression models.

† Adjusted for age, gender, BMI, smoking status.

Abbreviations: LDL-C, low-density lipoprotein cholesterol; SD, standard deviation; 95%CI, 95% confidence interval.

**Table S14. Effects of *LDLR* and *PCSK9* rare variants on LDL-cholesterol levels for male subjects.**

| Gene | Category | N | LDL-C (mg/dl) | Change from Non-Carriers of Rare *LDLR*/*PCSK9* Variants | | | |
| --- | --- | --- | --- | --- | --- | --- | --- |
|  |  |  | mean ± SD | Crude (95%CI) | *P* | Adjusted† (95%CI) | *P* |
| Non-Carriers of  Rare *LDLR*/*PCSK9* Variants | | 3901 | 110.01 ± 30.66 |  |  |  |  |
| *LDLR* | non-synonymous | 205 | 115.92 ± 38.45 | +5.92 (+1.55, +10.28) | 8.0 x 10^-3^ | +5.71 (+1.42, +10.01) | 9.1 x 10^-3^ |
| *LDLR* | Damaging | 26 | 146.91 ± 31.44 | +36.90 (+25.07, +48.73) | 1.06 x10^-9^ | +38.65 (+27.02, +50.28) | 8.19 x 10^-11^ |
| *LDLR* | Disruptive | 19 | 156.92 ± 35.48 | +46.91 (+33.08, +60.75) | 3.39 x 10^-11^ | +50.27 (+36.63, +63.90) | 5.96 x 10^-13^ |
| *PCSK9* | E32K | 144 | 123.79 ± 35.34 | +13.79 (+8.66, +18.92) | 1.45 x 10^-7^ | +15.54 (+10.49, +20.59) | 1.79 x 10^-9^ |
| *PCSK9* | R93C | 74 | 99.47 ± 34.54 | -10.53 (-17.61, -3.46) | 3.5 x 10^-3^ | -12.51 (-19.46, -5.56) | 4.2 x 10^-4^ |
| *PCSK9* | Disruptive | 2 | 75.30 ± 26.73 | -34.71 (-77.22, +7.81) | 0.11 | -39.83 (-81.57, +1.91) | 0.061 |

Changes from non-carriers and confidence intervals were calculated from linear regression models.

† Adjusted for age, BMI, smoking status and cholesterol lowering medications.

Abbreviations: LDL-C, low-density lipoprotein cholesterol; SD, standard deviation; 95%CI, 95% confidence interval.

**Table S15. Effects of *LDLR* and *PCSK9* rare variants on LDL-cholesterol levels for female subjects.**

| Gene | Category | N | LDL-C (mg/dl) | Change from Non-Carriers of Rare *LDLR*/*PCSK9* Variants | | | |
| --- | --- | --- | --- | --- | --- | --- | --- |
|  |  |  | mean ± SD | Crude (95%CI) | *P* | Adjusted† (95%CI) | *P* |
| Non-Carriers of  Rare *LDLR*/*PCSK9* Variants | | 1179 | 120.55 ± 33.32 |  |  |  |  |
| *LDLR* | non-synonymous | 56 | 121.70 ± 36.20 | +1.15 (-7.82, +10.13) | 0.8 | +2.45 (-6.42, +11.31) | 0.59 |
| *LDLR* | Damaging | 8 | 181.65 ± 43.97 | +61.10 (+37.86, +84.34) | 2.91 x10^-7^ | +63.48 (+40.59, +86.38) | 6.49 x 10^-8^ |
| *LDLR* | Disruptive | 2 | 144.90 ± 12.59 | +24.35 (-21.89, +70.59) | 0.3 | +31.63 (-13.96, +77.21) | 0.17 |
| *PCSK9* | E32K | 42 | 145.70 ± 58.66 | +25.15 (+14.53, +35.77) | 3.74 x 10^-6^ | +26.79 (+16.30, +37.29) | 6.33 x 10^-7^ |
| *PCSK9* | R93C | 28 | 109.34 ± 35.49 | -11.21 (-23.73, +1.31) | 0.079 | -12.13 (-24.50, +0.25) | 0.055 |
| *PCSK9* | Disruptive | 2 | 86.10 ± 37.19 | -34.45 (-80.71, +11.81) | 0.14 | -37.54 (-83.13, +8.05) | 0.11 |

Changes from non-carriers and confidence intervals were calculated from linear regression models.

† Adjusted for age, BMI, smoking status and cholesterol lowering medications.

Abbreviations: LDL-C, low-density lipoprotein cholesterol; SD, standard deviation; 95%CI, 95% confidence interval.

**Table S16. Effects of newly-identified *LDLR* and *PCSK9* rare variants on onset age of MI.**

| Gene | Category | n | Onset Age of MI  (year old) | Change from Non-Carriers of Rare *LDLR*/*PCSK9* Variants | | | |
| --- | --- | --- | --- | --- | --- | --- | --- |
|  |  |  | mean ± SD | Crude (95%CI) | *P* | Adjusted† (95%CI) | *P* |
| Non-Carriers of  Rare *LDLR*/*PCSK9* Variants | | 5375 | 61.01 ± 10.63 |  |  |  |  |
| *LDLR* | Non synonymous | 127 | 61.75 ± 10.21 | +0.74 (-1.17, +2.65) | 0.45 | +0.60 (-1.25, +2.46) | 0.52 |
| *LDLR* | Damaging | 4 | 52.00 ± 1.73 | -9.01 (-21.05, +3.03) | 0.14 | -9.09 (-22.76, +4.58) | 0.19 |
| *LDLR* | Disruptive | 3 | 41.50 ± 16.26 | -19.51 (-34.26, -4.76) | 9.50 x 10^-3^ | -16.37 (-30.05, -2.70) | 0.019 |
| *PCSK9* | Disruptive | 1 | 70 ± NA | +8.99 (-11.86, +29.84) | 0.40 | +3.89 (-15.45, +23.24) | 0.69 |

Changes from non-carriers and confidence intervals were calculated from linear regression models.

† Adjusted for gender, BMI, smoking status and cholesterol lowering medications.

Abbreviations: SD, standard deviation; 95%CI, 95% confidence interval.

**Table S17. Effects of *LDLR* and *PCSK9* rare variants on onset age of MI in the patients without cholesterol-lowering drugs.**

| Gene | Category | N | Onset Age of MI  (year old) | Change from Non-Carriers of Rare *LDLR*/*PCSK9* Variants | | | |
| --- | --- | --- | --- | --- | --- | --- | --- |
|  |  |  | mean ± SD | Crude (95%CI) | *P* | Adjusted† (95%CI) | *P* |
| Non-Carriers of  Rare *LDLR*/*PCSK9* Variants | | 3108 | 62.42 ± 10.62 |  |  |  |  |
| *LDLR* | non-synonymous | 148 | 61.17 ± 10.15 | -1.25 (-3.00, +0.50) | 0.16 | -0.9 (-2.57, +0.76) | 0.29 |
| *LDLR* | Damaging | 12 | 59.42 ± 11.77 | -3.00 (-9.02, +3.02) | 0.33 | -3.19 (-8.94, +2.56) | 0.28 |
| *LDLR* | Disruptive | 4 | 55.25 ± 13.50 | -7.17 (-17.59, +3.25) | 0.18 | -6.54 (-16.48, +3.40) | 0.2 |
| *PCSK9* | E32K | 96 | 61.44 ± 9.85 | -0.98 (-3.13, +1.17) | 0.37 | -1.43 (-3.48, +0.63) | 0.17 |
| *PCSK9* | R93C | 74 | 62.62 ± 10.95 | +0.20 (-2.25, +2.65) | 0.87 | +0.16 (-2.18, +2.50) | 0.9 |
| *PCSK9* | Disruptive | 5 | 73.20 ± 11.82 | +10.78 (+1.46, +20.10) | 0.023 | +7.97 (-0.93, +16.87) | 0.079 |

Changes from non-carriers and confidence intervals were calculated from linear regression models.

† Adjusted for gender, BMI and smoking status.

Abbreviations: SD, standard deviation; 95%CI, 95% confidence interval.

**Table S18. Effects of *LDLR* and *PCSK9* rare variants on onset age of MI in the patients with cholesterol-lowering drugs.**

| Gene | Category | N | Onset Age of MI  (year old) | Change from Non-Carriers of Rare *LDLR*/*PCSK9* Variants | | | |
| --- | --- | --- | --- | --- | --- | --- | --- |
|  |  |  | mean ± SD | Crude (95%CI) | *P* | Adjusted† (95%CI) | *P* |
| Non-Carriers of  Rare *LDLR*/*PCSK9* Variants | | 2983 | 59.73 ± 10.47 |  |  |  |  |
| *LDLR* | non-synonymous | 169 | 59.83 ± 10.94 | +0.11 (-1.52, +1.73) | 0.9 | -0.35 (-1.86, +1.15) | 0.64 |
| *LDLR* | Damaging | 31 | 54.84 ± 11.22 | -4.89 (-8.60, -1.18) | 9.8 x 10^-3^ | -5.65 (-9.08, -2.23) | 1.2 x 10^-3^ |
| *LDLR* | Disruptive | 27 | 47.59 ± 10.45 | -12.14 (-16.11, -8.17) | 2.28 x 10^-9^ | -11.92 (-15.58, -8.25) | 2.02 x 10^-10^ |
| *PCSK9* | E32K | 157 | 57.06 ± 11.08 | -2.66 (-4.35, -0.98) | 2.0 x 10^-3^ | -2.71 (-4.26, -1.15) | 6.7 x 10^-4^ |
| *PCSK9* | R93C | 38 | 59.16 ± 8.81 | -0.57 (-3.92, +2.78) | 0.74 | +0.31 (-2.78, +3.41) | 0.84 |
| *PCSK9* | Disruptive | 1 | 77 | +17.27 (-3.27, +37.81) | 0.099 | +9.85 (-9.13, +28.83) | 0.31 |

Changes from non-carriers and confidence intervals were calculated from linear regression models.

† Adjusted for gender, BMI and smoking status.

Abbreviations: SD, standard deviation; 95%CI, 95% confidence interval.

**Table S19. Effects of *LDLR* and *PCSK9* rare variants on onset age of MI for male patients.**

| Gene | Category | N | Onset Age of MI  (year old) | Change from Non-Carriers of Rare *LDLR*/*PCSK9* Variants | | | |
| --- | --- | --- | --- | --- | --- | --- | --- |
|  |  |  | mean ± SD | Crude (95%CI) | *P* | Adjusted† (95%CI) | *P* |
| Non-Carriers of  Rare *LDLR*/*PCSK9* Variants | | 5020 | 60.04 ± 10.48 |  |  |  |  |
| *LDLR* | non-synonymous | 254 | 59.09 ± 10.50 | -0.94 (-2.26, +0.38) | 0.16 | -0.79 (-2.05, +0.47) | 0.22 |
| *LDLR* | Damaging | 35 | 54.71 ± 11.27 | -5.32 (-8.81, -1.83) | 2.8 x 10^-3^ | -4.93 (-8.25, -1.60) | 3.7 x 10^-3^ |
| *LDLR* | Disruptive | 27 | 47.07 ± 10.54 | -12.96 (-16.93, -9.00) | 1.61 x 10^-10^ | -11.62 (-15.40, -7.84) | 1.80 x 10^-9^ |
| *PCSK9* | E32K | 202 | 57.40 ± 10.66 | -2.64 (-4.11, -1.16) | 4.7 x 10^-4^ | -2.40 (-3.81, -1.00) | 8.2 x 10^-4^ |
| *PCSK9* | R93C | 91 | 60.91 ± 10.08 | +0.88 (-1.30, +3.05) | 0.43 | +0.63 (-1.44, +2.70) | 0.55 |
| *PCSK9* | Disruptive | 2 | 62.50 ± 3.54 | +2.46 (-12.07, +17.00) | 0.74 | +2.01 (-11.83, +15.84) | 0.78 |

Changes from non-carriers and confidence intervals were calculated from linear regression models.

† Adjusted for BMI and smoking status and cholesterol lowering medications.

Abbreviations: SD, standard deviation; 95%CI, 95% confidence interval.

**Table S20. Effects of *LDLR* and *PCSK9* rare variants on onset age of MI for female patients.**

| Gene | Category | N | Onset Age of MI  (year old) | Change from Non-Carriers of Rare *LDLR*/*PCSK9* Variants | | | |
| --- | --- | --- | --- | --- | --- | --- | --- |
|  |  |  | mean ± SD | Crude (95%CI) | *P* | Adjusted† (95%CI) | *P* |
| Non-Carriers of  Rare *LDLR*/*PCSK9* Variants | | 1071 | 66.09 ± 9.88 |  |  |  |  |
| *LDLR* | non-synonymous | 63 | 65.95 ± 9.09 | -0.13 (-2.63, +2.37) | 0.92 | +0.11 (-2.33, +2.54) | 0.93 |
| *LDLR* | Damaging | 8 | 62.25 ± 10.66 | -3.84 (-10.72, +3.04) | 0.27 | -4.10 (-10.77, +2.56) | 0.23 |
| *LDLR* | Disruptive | 4 | 58.75 ± 8.77 | -7.34 (-17.04, +2.37) | 0.14 | -8.10 (-17.50, +1.30) | 0.091 |
| *PCSK9* | E32K | 51 | 63.96 ± 9.90 | -2.13 (-4.90, +0.65) | 0.13 | -1.83 (-4.52, +0.85) | 0.18 |
| *PCSK9* | R93C | 21 | 63.76 ± 11.49 | -2.32 (-6.61, +1.96) | 0.29 | -1.36 (-5.52, +2.80) | 0.52 |
| *PCSK9* | Disruptive | 4 | 79.50 ± 7.59 | +13.41 (+3.71, +23.11) | 6.8 x 10^-3^ | +12.76 (+3.36, +22.16) | 7.9 x 10^-3^ |

Changes from non-carriers and confidence intervals were calculated from linear regression models.

† Adjusted for BMI and smoking status and cholesterol lowering medications.

Abbreviations: SD, standard deviation; 95%CI, 95% confidence interval.
